# Revision of *Muhlenbergia* (Poaceae, Chloridoideae, Cynodonteae, Muhlenbergiinae) in Peru: classification, phylogeny, and a new species, *M.romaschenkoi*

**DOI:** 10.3897/phytokeys.114.28799

**Published:** 2018-12-31

**Authors:** Paul M. Peterson, Isidoro Sánchez Vega, Konstantin Romaschenko, Diego Giraldo-Cañas, Nancy F. Refulio Rodriguez

**Affiliations:** 1 Department of Botany MRC-166, National Museum of Natural History, Smithsonian Institution, Washington, DC 20013-7012, USA National Museum of Natural History, Smithsonian Institution Washington United States of America; 2 Departamento de Biología, Herbario CPUN, Universidad Nacional de Cajamarca Apartado 55, Cajamarca, Perú Universidad Nacional de Cajamarca Cajamarca Peru; 3 Herbario Nacional Colombiano, Instituto de Ciencias Naturales, Universidad Nacional de Colombia, Bogotá DC, Colombia Universidad Nacional de Colombia Bogotá Colombia; 4 ResearchGate, Invalidenstraße 115, 10115 Berlin, Germany ResearchGate Berlin Germany

**Keywords:** classification, ITS, lectotypification, *
Muhlenbergia
*, Peru, phylogeny, plastid DNA sequences, Poaceae, systematics, taxonomy

## Abstract

A taxonomic treatment, phylogeny based on analysis of six DNA sequence markers (ITS, *ndhA* intron, *rpl32-trnL*, *rps3*, *rps16* intron and *rps16-trnK*) and classification of *Muhlenbergia* for Peru is given. Seventeen species and one presumed hybrid are recognised. *Muhlenbergiaromaschenkoi***sp. nov.** is newly described from the Río Huallaga Valley, northeast of Huánuco. The type of *Podosemumangustatum* [≡ *Muhlenbergiaangustata*] clearly aligns with what we had been referring to as the hybrid between this species and *M.rigida*. Therefore, we adopt the next available heterotypic name, *Muhlenbergiacoerulea*, for what we had been calling *M.angustata* and change the hybrid designation to *M.coerulea* × *M.rigida*. Lectotypes are designated for *Epicampescoerulea* Griseb., *Muhlenbergiaaffinis* Trin., *Muhlenbergiaberlandieri* Trin., *Muhlenbergiabeyrichiana* Kunth, Muhlenbergiaelegansvar.atroviolacea Kuntze, Muhlenbergiaelegansvar.subviridis Kuntze and *Muhlenbergiaphragmitoides* Griseb.

## Abstract (es)

Brindamos un tratamiento taxonómico, una filogenia basado en el análisis de seis marcadores de secuencia de ADN (ITS, *intrón ndhA*, *rpl32-trnL, rps3, intrón rps16, rps16-trnK*) y la clasificación de *Muhlenbergia* para Perú. Se reconocen diecisiete especies y un supuesto híbrido. *Muhlenbergiaromaschenkoi***sp. nov.** es descrita como especie nueva procedente del valle de Huallaga, al noreste de Huánuco. El tipo de *Podosemumangustatum* [≡ *Muhlenbergiaangustata*] se alinea claramente con lo que nosotros habíamos referido como el híbrido entre esta especie y *M.rigida*. Por lo tanto, adoptamos el siguiente nombre heterotípico disponible, *Muhlenbergiacoerulea* para lo que habíamos estado llamando *M.angustata*, y cambiamos la designación híbrida a *M.coerulea* × *M.rigida*. Los lectotipos son designados para *Epicampescoerulea* Griseb., *Muhlenbergiaaffinis* Trin., *Muhlenbergiaberlandieri* Trin., *Muhlenbergiabeyrichiana* Kunth, Muhlenbergiaelegansvar.atroviolacea Kuntze, Muhlenbergiaelegansvar.subviridis Kuntze y *Muhlenbergiaphragmitoides* Griseb.

## Introduction

Peru is located in the central and western part of South America between 0° and 18° south latitude. The central Andes or backbone divides Peru into three great regions: coast, mountain, and forest (Weberbauer 1936). The coastal region next to the Pacific Ocean is an extremely xeric, narrow, sandy plain that rises abruptly to the east. This region is occasionally transected by river valleys and the climate is very mild and warm. The mountain region or ‘‘cordillera de Andes’’ consists of high peaks and extensive high plains (altiplano) that are sometimes dissected by deep, narrow valleys. The climate in this region can be very cold and it is not uncommon to have snow above 3800 m. The tropical forest region, east of the cordillera, extends into the Amazonian river basin where there are high levels of biodiversity associated with many different habitats. The climate here is very warm and humid with high levels of precipitation and there are large rivers including the Río Marañon and the Río Ucayali that feed into the Río Amazonas.

The Poaceae is a diverse family occupying a myriad of terrestrial habitats in Peru, with the exception of the permanent snow fields. The taxonomic knowledge of this family in Peru is incomplete and it is still possible to encounter novel species.

Hitchcock (1927), in his treatment of the grasses of Ecuador, Peru and Bolivia, recognised 12 species of *Muhlenbergia* Schreb., 10 of these being reported in Peru. Standley (1936), in his Flora of Peru, recorded 10 species. More recently, Tovar (1993) produced an integral study of the Peruvian grasses, including 14 species of *Muhlenbergia*. Brako and Zarucchi (1993) list 13 species as being present in Peru. All of these treatments are out of date and, subsequently, there have been new reports of grasses from Peru. As *Muhlenbergia* is a relatively large genus in North and Central America and is easily dispersed, a modern investigation was undertaken to more clearly understand its systematics and biogeography. In Peru, we report 17 species of *Muhlenbergia* (one of these is new) and one proposed hybrid.

The subtribe Muhlenbergiinae Pilg. (tribe Cynodonteae Dumort.) is a diverse assemblage of 182 species represented by a single, monophyletic genus, *Muhlenbergia* (Peterson et al. 2010a, b; 2016; Soreng et al. 2017; Peterson et al. 2018). Species within *Muhlenbergia* are morphologically highly variable and are characterised in having membranous ligules (rarely a line of hairs); paniculate inflorescences that are rebranched or composed only of primary branches; spikelets that are usually solitary but sometimes in pairs or triads, with cleistogenes (self-pollinated flowers that do not open at maturity) occasionally present in the leaf sheaths; one floret (rarely more) per spikelet that is perfect, staminate or sterile; glumes that are awned or unawned; lemmas 3-nerved, apex awned or unawned; and a base chromosome number of *x* = 8−10 (Peterson et al. 1995, 1997, 2007a, b; Peterson 2000, 2003). Two subtypes of C_4_ photosynthesis based on nicotinamide adenine dinucleotide cofactor malic enzyme (NAD-ME) and phosphoenolpyruvate carboxykinase (PCK) have been found in *Muhlenbergia*; subtypes in most species have been verified by anatomy and in a few species by biochemical assay (Gutierrez et al. 1974; Brown 1977; Hattersley and Watson 1992).

Based on seven molecular markers (nuclear ITS and plastid *ndhA* intron, *ndhF*, *rps16-trnK, rps16* intron, *rps3* and *rpl32-trnL*), Peterson et al. (2010b) provided a phylogeny and classification for 124 species (68%) of the Muhlenbergiinae. They recognised five subgenera within *Muhlenbergia*: M.subg.Bealia (Scribn.) P.M. Peterson, M.subg.Clomena (P. Beauv.) Hack., M.subg.Muhlenbergia, M.subg.Pseudosporobolus (Parodi) P.M. Peterson and M.subg.Trichochloa (P. Beauv.) A. Gray. Here we present an updated phylogeny of 146 species (80%) of *Muhlenbergia* with 13 new species based on six molecular markers (nuclear ITS and plastid *ndhA* intron, *rpl32-trnL*, *rps3, rps16* intron and *rps16-trnK*), classification and a taxonomic revision of the genus for Peru.

## Material and methods

### Phylogenetic analyses

Detailed methods for DNA extraction, amplification, sequencing and phylogenetic analysis are given in Peterson et al. (2010a, b, 2014a, 2015a, b, 2016). In brief, the phylogeny was estimated among members of *Muhlenbergia* based on the analysis of six molecular markers (nuclear ITS 1&2, and plastid *ndhA* intron, *rpl32-trnL*, *rps3, rps16* intron, *rps16-trnK*). We sampled most species within subtribe Muhlenbergiinae and included outgroups: *Distichlisscoparia* (Nees ex Kunth) Arechav. (Monanthochloinae Pilg. ex Potztal), *Willkommiasarmentosa* Hack. (Traginae P.M. Peterson & Columbus) and *Sporobolusindicus* L. (Zoysieae Benth., Sporobolinae Benth.) [Peterson et al. 2010a, 2015a, 2014b, 2016; Soreng et al. 2017]. Voucher information and GenBank numbers for all samples (including the new ones) are given in Table 1.

**Figure 1.**
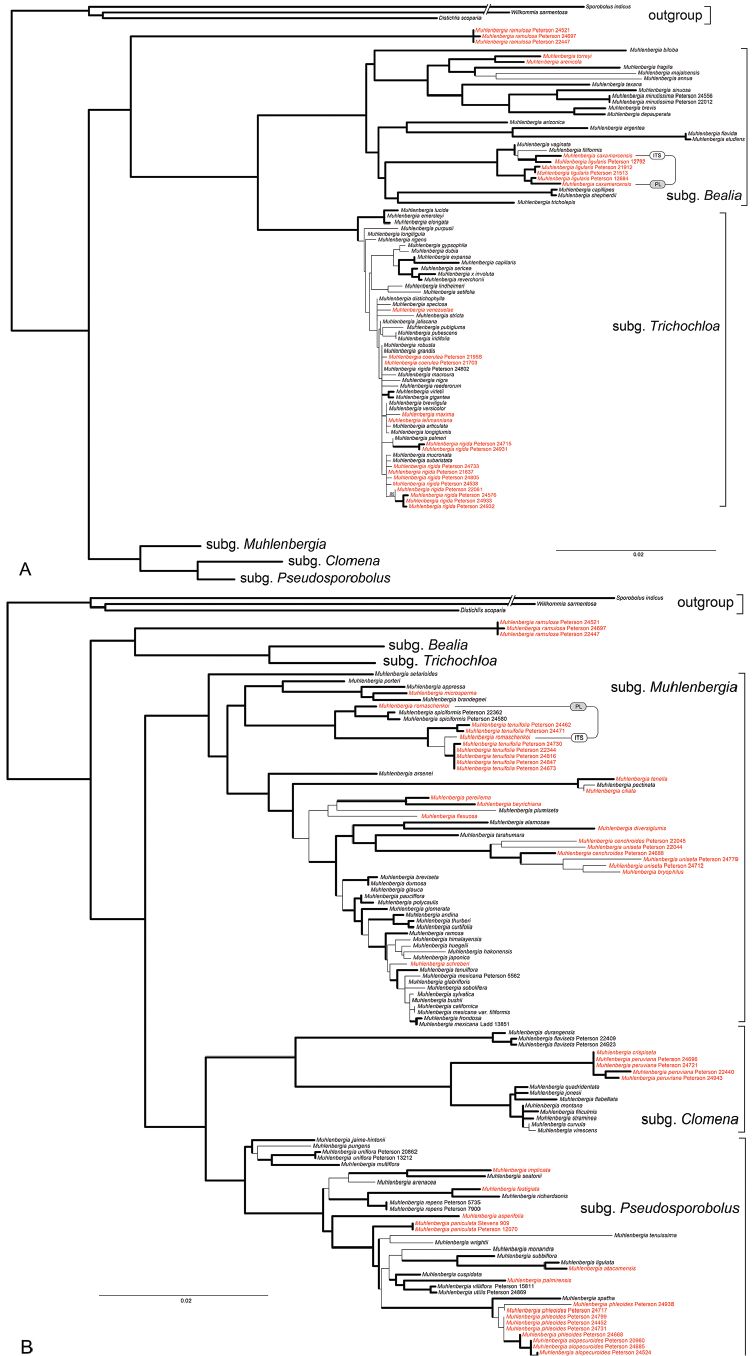
**A, B** Maximum-likelihood tree inferred from combined plastid (*ndhA* intron, *rps16-trnK, rps16* intron, *rps3*, and *rpl32-trnL*) and ITS sequences. Thick branches indicate posterior probabilities of 0.95−1; species in red occur in South America; scale bar = 2%.

The resulting plastid and ITS topologies were inspected for conflicting nodes with posterior probabilities (PP) ≥ 0.95. If no supported conflict was found, plastid and ITS sequences were combined (Figure 1). When conflicting topologies were present, the datasets for inconsistently placed taxa were duplicated in the matrix. One set of the taxon was represented by only the corresponding plastid sequences, the other taxon set by only ITS sequences. The remaining positions for the duplicated datasets were then coded as missing data. We use this “taxon duplication” approach (Pirie et al. 2008; Pelser et al. 2010; Peterson et al. 2015b, 2016) to resolve our phylogenetic tree, minimising the diffusing effects of taxa with strongly supported incongruence between the plastid and ITS data and to represent their alternative placements in relation to the remaining phylogenetic groups, among which relationships are congruent.

**Table 1.** Taxon voucher (collector, number, and where the specimen is housed), country of origin, and GenBank accession for DNA sequences of *rps3, rps16-trnK, rps16* intron, *ndhA* intron, *rpl32-trnL*, and ITS regions (**bold** indicates new accession); a dash (−) indicates missing data; an asterisk (*) indicates sequences not generated in our lab.

N	Taxon	Voucher	Country	rps3	rps16-trnK	rps16 intron	ndhA intron	rpl32-trnL	ITS
Outgroup									
1	Distichlisscopariavar.erinacea (Nees ex Kunth) Arechav.	Peterson 17475, Soreng & Refulio-Rodriguez (US)	Argentina, Neuquen	GU360034	GU360501	GU360477	GU359480	GU359803	GU359334
2	*Sporobolusindicus* (L.) R. Br.	Peterson 22025 & Saarela (US)	Mexico, Chihuahua	GU360161	GU360630	GU360355	GU359504	GU359913	GU359209
3	*Willkommiasarmentosa* Hack.	Schweickerdt 2181 (US)	South Africa	GU360252	GU360645	GU360343	GU359545	GU359924	GU359194
Muhlenbergia									
4	*Muhlenbergiaramulosa* (Kunth) Swallen	Peterson 22447 & Saarela (US)	Mexico, Durango	GU360254	GU360717	GU360406	GU359444	GU359953	GU359115
5	*Muhlenbergiaramulosa* (Kunth) Swallen	Peterson 24521 & Romaschenko (US)	Mexico, Coahuila	**MK090816**	**MK090876**	**MK090848**	**MK090728**	**MK090773**	**MK090686**
6	*Muhlenbergiaramulosa* (Kunth) Swallen	Peterson 24697, Romaschenko & Zamudio Ruíz (US)	Mexico, Queretaro	**MK090817**	**MK090877**	**MK090849**	**MK090729**	**MK090774**	**MK090687**
subg. Bealia									
7	*Muhlenbergiaannua* (Vasey) Swallen	Peterson 22022 & Saarela (US)	Mexico, Chihuahua	HM143247	HM143629	HM143534	HM143351	HM143144	HM143043
8	*Muhlenbergiaarenicola* Buckley	Peterson 19947 & Lara-Contreras (US)	Mexico, Coahuila	GU360209	GU360674	GU360413	GU359462	GU359960	GU359166
9	*Muhlenbergiaargentea* Vasey	Peterson 22095 & Saarela (US)	Mexico, Chihuahua	HM143248	HM143630	HM143535	HM143352	HM143145	HM143044
10	*Muhlenbergiaarizonica* Scribn.	Peterson 22173 & Saarela (US)	Mexico, Chihuahua	HM143249	HM143631	HM143536	HM143353	HM143146	HM143045
11	*Muhlenbergiabiloba* Hitchc.	Peterson 7946, Annable & Herrera (US)	Mexico, Chihuahua	GU360098	GU360550	GU360309	–	GU359859	GU359258
12	*Muhlenbergiabrevis* C.O. Goodd.	Peterson 22023 & Saarela (US)	Mexico, Chihuahua	HM143253	HM143635	HM143540	HM143357	HM143150	HM143049
13	*Muhlenbergiacapillipes* (M.E. Jones) P.M. Peterson & Annable	Peterson 20013 & Sánchez Alvarado (US)	Mexico, Durango	**MK090802**	**MK090862**	**MK090835**	**MK090714**	**MK090751**	**MK090664**
14	*Muhlenbergiacaxamarcensis* Lægaard & Sánchez Vega	Peterson 21965, Soreng & Montoya Quino (US)	Peru, La Libertad	HM143256	HM143639	HM143543	HM143360	HM143153	HM143052
15	*Muhlenbergiadepauperata* Scribn.	Peterson 21293, Saarela & Flores Villegas (US)	Mexico, Zacatecas	HM143261	HM143642	HM143547	HM143363	HM143157	HM143056
16	*Muhlenbergiaeludens* C. Reeder	Peterson 22188 & Saarela (US)	Mexico, Sinaloa	HM143268	HM143649	HM143554	HM143370	HM143164	HM143062
17	*Muhlenbergiafiliformis* (Thurb. ex S. Watson) Rydb.	Peterson 10433, Annable & Weinpahl (US)	USA, California	HM143272	HM143653	HM143558	HM143374	HM143168	HM143066
18	*Muhlenbergiaflavida* Vasey	Peterson 22237 & Saarela (US)	Mexico, Sinaloa	HM143273	HM143654	HM143559	HM143375	HM143169	HM143067
19	*Muhlenbergiafragilis* Swallen	Peterson 22194 & Saarela (US)	Mexico, Sinaloa	HM143275	HM143656	HM143561	HM143377	HM143171	HM143069
20	*Muhlenbergialigularis* (Hack.) Hitchc.	Peterson 12684, Annable, Lægaard & Soreng (US)	Bolivia, La Paz	**MK090808**	**MK090868**	**MK090842**	**MK090720**	**MK090760**	**MK090674**
21	*Muhlenbergialigularis* (Hack.) Hitchc.	Peterson 12792, Annable, Lægaard & Soreng (US)	Bolivia, Oruro	**MK090809**	**MK090869**	**MK090843**	**MK090721**	**MK090761**	**MK090675**
22	*Muhlenbergialigularis* (Hack.) Hitchc.	Peterson 21513, Soreng, Torre & Rojas Fox (US)	Peru, Ancash	**MK090810**	**MK090870**	**MK090844**	**MK090722**	**MK090762**	**MK090676**
23	*Muhlenbergialigularis* (Hack.) Hitchc.	Peterson 21912, Soreng & Montoya Quino (US)	Peru, Cajamarca	**MK090811**	**MK090871**	**MK090845**	**MK090723**	**MK090763**	**MK090677**
24	*Muhlenbergiamajalcensis* P.M. Peterson	Peterson 4519 & Annable (US)	Mexico, Chihuahua	HM143295	HM143675	HM143577	HM143394	HM143190	HM143088
25	*Muhlenbergiaminutissima* (Steud.) Swallen	Peterson 22012 & Saarela (US)	Mexico, Chihuahua	HM143300	HM143680	−	−	HM143195	HM143093
26	*Muhlenbergiaminutissima* (Steud.) Swallen	Peterson 24556 & Romaschenko (US)	Mexico, Coahuila	**MK090813**	**MK090873**	−	−	**MK090765**	**MK090679**
27	*Muhlenbergiashepherdii* (Vasey) Swallen	Peterson 22452 & Saarela (US)	Mexico, Durango	GU360102	GU360560	GU360320	GU359419	GU359854	GU359277
28	*Muhlenbergiasinuosa* Swallen	Peterson 7976, Annable & Herrera Arrieta (US)	Mexico, Chihuahua	HM143323	HM143704	HM143603	HM143419	HM143219	HM143117
29	*Muhlenbergiatexana* Buckley	Peterson 22016 & Saarela (US)	Mexico, Chihuahua	HM143334	HM143714	HM143614	−	HM143230	HM143128
30	*Muhlenbergiatorreyi* (Kunth) Hitchc. ex Bush	Peterson 19429, Soreng, Salariato & Panizza (US)	Argentina, Catamarca	GU360266	GU360720	GU360267	**MK090744**	GU359992	GU359118
31	*Muhlenbergiatricholepis* (Torr.) Columbus	Peterson 22099 & Saarela (US)	Mexico, Chihuahua	GU360103	GU360559	GU360305	GU359418	GU359853	GU359278
32	*Muhlenbergiavaginata* Swallen	Peterson 22417 & Saarela (US)	Mexico, Durango	−	HM143718	HM143618	**MK090745**	HM143234	**MK090708**
subg. Clomena									
33	*Muhlenbergiacrispiseta* Hitchc.	Peterson 10768, Annable & Valdes Reyna (US)		HM143258	−	−	−	−	−
34	*Muhlenbergiacurvula* Swallen	Peterson 24830, Romaschenko, Rodriguez Avalos, Herrera-Simoni & Garcia Rodriguez (US)	Mexico, Aguascalientes	−	−	−	−	**MK090754**	**MK090667**
35	*Muhlenbergiaflaviseta* Scribn.	Peterson 22409 & Saarela (US)	Mexico, Durango	GU360250	GU360685	GU360410	GU359448	GU359957	GU359127
36	*Muhlenbergiaflaviseta* Scribn.	Peterson 24923, Romaschenko & González-Elizondo (US)	Mexico, Durango	−	−	**MK090839**	−	**MK090757**	**MK090670**
37	*Muhlenbergiafiliculmis* Vasey	Peterson 11954 & Annable (US)	USA, Wyoming	HM143271	HM143652	HM143557	HM143373	HM143167	HM143065
38	*Muhlenbergiaflabellata* Mez	Senalobo 1985 (MO)	Costa Rica,	**MK090805**	**MK090865**	**MK090838**	**MK090717**	**MK090756**	**MK090669**
39	*Muhlenbergiajonesii* (Vasey) Hitchc.	Peterson 4861 & Annable (US)	USA, California	HM143289	HM143669	−	HM143390	HM143185	HM143083
40	*Muhlenbergiamontana* (Nutt.) Hitchc.	Peterson 22046 & Saarela (US)	Mexico, Chihuahua	HM143301	HM143681	HM143582	HM143398	HM143196	HM143094
41	*Muhlenbergiaperuviana* (P. Beauv.) Steud.	Peterson 22440 & Saarela (US)	Mexico, Durango	GU360221	GU360713	GU360408	GU359446	GU359955	GU359154
42	*Muhlenbergiaperuviana* (P. Beauv.) Steud.	Peterson 24696, Romaschenko & Zamudio Ruíz (US)	Mexico, Querétaro	−	−	−	−	**MK090767**	**MK090680**
43	*Muhlenbergiaperuviana* (P. Beauv.) Steud.	Peterson 24721, Romaschenko & Zamudio Ruíz (US)	Mexico, Querétaro	−	−	−	−	**MK090768**	**MK090681**
44	*Muhlenbergiaperuviana* (P. Beauv.) Steud.	Peterson 24943, Romaschenko & Zamudio Ruíz (US)	Mexico, Querétaro	−	−	−	−	**MK090769**	**MK090682**
45	*Muhlenbergiaquadridentata* (Kunth) Trin.	Peterson 16103 & Rosales (US)	Mexico, Jalisco	HM143313	HM143694	−	HM143410	HM143209	HM143107
46	*Muhlenbergiastraminea* Hitchc.	Peterson 15238 & Cayouette (US)	USA, Arizona	HM143327	HM143707	HM143607	**MK090737**	HM143223	HM143121
47	*Muhlenbergiavirescens* (Kunth) Trin.	Peterson 22412 & Saarela (US)	Mexico, Durango	HM143342	HM143722	HM143621	HM143432	HM143238	HM143134
subg. Muhlenbergia									
48	*Muhlenbergiaalamosae* Vasey	Peterson 22104 & Saarela (US)	Mexico, Chihuahua	HM143243	HM143626	HM143530	HM143347	HM143140	HM143039
49	*Muhlenbergiaandina* (Nutt.) Hitchc.	Peterson 10432, Annable & Weinpahl (US)	USA, California	HM143244	−	HM143531	HM143348	HM143141	HM143040
50	*Muhlenbergiaappressa* C.O. Goodd.	Peterson 4183 & Annable (US)	USA, Arizona	GU360211	GU360676	GU360415	GU359443	GU359962	GU359164
51	*Muhlenbergiaarsenei* Hitchc.	Peterson 15208 & Cayouette (US)	Mexico, Baja California Norte	HM143250	HM143632	HM143537	HM143354	HM143147	HM143046
52	*Muhlenbergiabeyrichiana* Kunth	Peterson 20366, Soreng & Romaschenko (US)	Peru, Huánuco	GU360247	GU360712	GU360280	GU359493	GU359995	GU359129
53	*Muhlenbergiabrandegeei* C. Reeder	Peterson 4760 & Annable (US)	Mexico, Baja California Sur	GU360208	GU360711	GU360412	GU359450	GU359959	GU359167
54	*Muhlenbergiabreviseta* Griseb. ex E. Fourn.	McVaugh 22930 (US)	Mexico	−	HM143636	−	−	−	−
55	*Muhlenbergiabryophilus* (Döll) P.M. Peterson	Columbus 3565 (RSA)	Peru, Cusco	−	−	−	−	−	GQ397862*
56	*Muhlenbergiabushii* R.W. Pohl	Brant 4542 & O’Donnell (MO)	USA, Missouri	**MK090801**	**MK090861**	**MK090834**	**MK090713**	**MK090750**	**MK090663**
57	*Muhlenbergiacalifornica* Vasey	Peterson 5013 & Barron (US)	USA, California	HM143254	HM143637	HM143541	HM143358	HM143151	HM143050
58	*Muhlenbergiacenchroides* (Humb. & Bonpl. ex Willd.) P.M. Peterson	Peterson 22045 & Saarela (US)	Mexico, Chihuahua	GU360143	GU360578	GU360274	GU359403	GU360011	GU359259
59	*Muhlenbergiacenchroides* (Humb. & Bonpl. ex Willd.) P.M. Peterson	Peterson 24688, Romaschenko & Zamudio Ruíz (US)	Mexico, Querétaro	−	−	−	−	**MK090752**	**MK090665**
60	*Muhlenbergiaciliata* (Kunth) Trin.	Peterson 22193 & Saarela (US)	Mexico, Sinaloa	HM143257	HM143640	HM143544	HM143361	HM143154	HM143053
61	*Muhlenbergiacurtifolia* Scribn.	Peterson 5631 & Annable (US)	USA, Arizona	HM143259	HM143641	HM143545	HM143362	HM143155	HM143054
62	*Muhlenbergiadiversiglumis* Trin.	Peterson 4611 & Annable (US)	Mexico, Chihuahua	**MK090804**	**MK090864**	**MK090837**	**MK090716**	**MK090755**	**MK090668**
63	*Muhlenbergiadumosa* Scribn. ex Vasey	Peterson 13438, Knowles, Dietrich & Braxton (US)	Mexico, Durango	HM143265	HM143646	HM143551	HM143367	HM143161	HM143059
64	*Muhlenbergiaflexuosa* Hitchc.	Peterson 20373, Soreng & Romaschenko (US)	Peru, Huánuco	HM143274	HM143655	HM143560	HM143376	HM143170	HM143068
65	*Muhlenbergiafrondosa* (Poir.) Fernald	Yatskievych 04-224, Smith, McKenzie & Nagel (MO)	USA, Missouri	**MK090806**	**MK090866**	**MK090840**	**MK090718**	**MK090758**	**MK090671**
66	*Muhlenbergiaglabrifloris* Scribn.	Brant 2659 (MO)	USA, Missouri	**MK090807**	**MK090867**	**MK090841**	**MK090719**	**MK090759**	**MK090672**
67	*Muhlenbergiaglauca* (Nees) B.D. Jacks.	Peterson 21180, Saarela, Gonzalez Elizondo, Rosen & Reid (US)	Mexico, Durango	HM143277	HM143659	HM143564	HM143380	HM143174	HM143071
68	*Muhlenbergiaglomerata* (Willd.) Trin.	Peterson 20924, Saarela & Howard (US)	USA, New York	GU360253	GU360716	GU360407	GU359445	GU359954	GU359114
69	*Muhlenbergiahakonensis* (Hack. ex Matsum.) Makino	Kim 2009858	Korea (South)	−	−	−	−	−	HQ600507*
70	*Muhlenbergiahimalayensis* Hack. ex Hook. f.	Soreng 5666, Peterson & Sun Hang (US)	China, Xizang	HM143281	HM143662	HM143566	HM143383	HM143177	HM143075
71	*Muhlenbergiahuegelii* Trin.	Soreng 5344, Peterson & Sun Hang (US)	China, Sichuan	HM143282	HM143663	HM143567	HM143384	HM143178	HM143076
72	*Muhlenbergiajaponica* Steud.	Soreng 5240, Peterson & Sun Hang (US)	China, Yunnan	HM143287	HM143667	HM143571	HM143388	HM143183	HM143081
73	*Muhlenbergiamexicana* (L.) Trin.	Ladd 13851 (US)	USA, Missouri	**MK090812**	**MK090872**	**MK090846**	−	**MK090764**	**MK090678**
74	*Muhlenbergiamexicana* (L.) Trin.	Peterson 5562 & Annable (US)	USA, New Mexico	HM143297	HM143677	HM143580	HM143396	HM143193	HM143090
75	Muhlenbergiamexicanavar.filiformis (Torr.) Scribn.	Peterson 20861 & Saarela (US)	USA, New York.	HM143298	HM143678	HM143579	HM143395	HM143192	HM143091
76	*Muhlenbergiamicrosperma* (DC.) Kunth	Peterson 21855 & Soreng (US)	Peru, Cajamarca	HM143299	HM143679	HM143581	HM143397	HM143194	HM143092
77	*Muhlenbergiapauciflora* Buckley	Peterson 22048 & Saarela (US)	Mexico, Chihuahua	−	HM143686	HM143587	HM143403	HM143201	HM143099
78	*Muhlenbergiapectinata* C.O. Goodd.	Peterson 22108 & Saarela (US)	Mexico, Sonora	HM143306	HM143687	HM143588	HM143404	HM143202	HM143100
79	*Muhlenbergiapereilema* P.M. Peterson	Peterson 22191 & Saarela (US)	Mexico, Sinaloa	GU360245	GU360710	GU360282	GU359519	GU359993	GU359131
80	*Muhlenbergiaplumiseta* Columbus	Peterson 20106, Hall, Alvarez Marvan & Alvarez Jimenez (US)	Mexico, Guerrero	GU360246	GU360719	GU360281	GU359516	GU359979	GU359130
81	*Muhlenbergiapolycaulis* Scribn.	Peterson 22092 & Saarela (US)	Mexico, Chihuahua	HM143307	HM143688	HM143589	**MK090727**	HM143203	HM143101
82	*Muhlenbergiaporteri* Scribn. ex Beal	Peterson 19846 & Lara Contreras (US)	Mexico, Coahuila	HM143308	HM143689	HM143590	HM143405	HM143204	HM143102
83	*Muhlenbergiaramosa* (Hack. ex Matsum.) Makino	Soreng 5302, Peterson & Sun Hang (US)	China, Yunnan	HM143314	HM143695	HM143595	HM143411	HM143210	HM143108
84	*Muhlenbergiaromaschenkoi* P.M. Peterson	Peterson 20331, Soreng & Romashchenko (US)	Peru, Huánuco	–	–	−	−	**MK090784**	**MK096270**
85	*Muhlenbergiaschreberi* J.F. Gmel.	Peterson 19443, Soreng, Salariato & Panizza (US)	Argentina, Tucuman	GU360214	GU360679	GU360404	GU359456	GU359950	GU359161
86	*Muhlenbergiasetarioides* E. Fourn.	Peterson 9897 & Annable (US)	Mexico, Oaxaca	**MK090823**	**MK090883**	**MK090853**	**MK090736**	**MK090785**	**MK090697**
87	*Muhlenbergiasobolifera* (Muhl. ex Willd.) Trin.	Peterson 20834 & Saarela (US)	USA, Virginia	HM143324	**MK090884**	HM143604	HM143420	HM143220	HM143118
88	*Muhlenbergiaspiciformis* Trin.	Peterson 22362 & Saarela (US)	Mexico, Oaxaca	HM143326	HM143706	HM143606	HM143422	HM143222	HM143120
89	*Muhlenbergiaspiciformis* Trin.	Peterson 24580, Romaschenko & Valdés-Reyna (US)	Mexico, Coahuila	−	−	−	−	**MK090786**	**MK090698**
90	*Muhlenbergiasylvatica* (Torr.) Torr. ex A. Gray	Brant 4783 (MO)	USA, Missouri	**MK090824**	**MK090885**	**MK090854**	**MK090738**	**MK090787**	**MK090699**
91	*Muhlenbergiatarahumara* P.M. Peterson & Columbus	Peterson 22053 & Saarela (US)	Mexico, Chihuahua	HM143330	HM143710	HM143610	HM143425	HM143226	HM143124
92	*Muhlenbergiatenella* (Kunth) Trin.	Peterson 22141 & Saarela (US)	Mexico, Chihuahua	HM143331	HM143711	HM143611	−	HM143227	HM143125
93	*Muhlenbergiatenuiflora* (Willd.) Britton, Sterns & Poggenb.	Peterson 15778 & Saarela (US)	USA, Virginia.	HM143332	HM143712	HM143612	HM143426	HM143228	HM143126
94	*Muhlenbergiatenuifolia* (Kunth) Kunth	Peterson 22344 & Saarela (US)	Mexico, Oaxaca	HM143333	HM143713	HM143613	HM143427	HM143229	HM143127
95	*Muhlenbergiatenuifolia* (Kunth) Kunth	Peterson 24462, Romaschenko & Valdés-Reyna (US)	Mexico, Coahuila	−	−	−	−	**MK090788**	**MK090700**
96	*Muhlenbergiatenuifolia* (Kunth) Kunth	Peterson 24471 & Romaschenko (US)	Mexico, Coahuila	−	−	−	−	**MK090789**	**MK090701**
97	*Muhlenbergiatenuifolia* (Kunth) Kunth	Peterson 24673 & Romaschenko (US)	Mexico, San Luis Potosí	**MK090825**	**MK090886**	−	**MK090739**	**MK090790**	**MK090702**
98	*Muhlenbergiatenuifolia* (Kunth) Kunth	Peterson 24730, Romaschenko & Zamudio Ruíz (US)	Mexico, Queretaro	**MK090826**	**MK090887**	−	**MK090740**	**MK090791**	**MK090703**
99	*Muhlenbergiatenuifolia* (Kunth) Kunth	Peterson 24816, Romaschenko, Rodriguez Avalos, Herrera-Simoni & Garcia Rodriguez (US)	Mexico, Aguascalientes	**MK090827**	**MK090888**	−	**MK090741**	**MK090792**	**MK090704**
100	*Muhlenbergiatenuifolia* (Kunth) Kunth	Peterson 24847 & Romaschenko (US)	Mexico, Zacatecas	**MK090828**	**MK090889**	**MK090855**	**MK090742**	**MK090793**	**MK090705**
101	*Muhlenbergiathurberi* (Scribn.) Rydb.	Peterson 5619 & Annable (US)	USA, Arizona	HM143335	HM143715	HM143615	HM143428	HM143231	HM143129
102	*Muhlenbergiauniseta* (Lag.) Columbus	Peterson 22044 & Saarela (US)	Mexico, Chihuahua	GU360128	GU360577	GU360278	GU359392	GU360012	GU359260
103	*Muhlenbergiauniseta* (Lag.) Columbus	Peterson 24712, Romaschenko & Zamudio Ruíz (US)	Mexico, Queretaro	−	−	−	−	**MK090794**	**MK090706**
104	*Muhlenbergiauniseta* (Lag.) Columbus	Peterson 24779, Romaschenko & Zamudio Ruíz (US)	Mexico, Queretaro	−	−	−	−	**MK090795**	**MK090707**
subg. Pseudosporobolus									
105	*Muhlenbergiaalopecuroides* (Griseb.) P.M. Peterson & Columbus	Peterson 20960, Saarela, Lara Contreras & Reyna Alvarez (US)	Mexico, Coahuila	GU360224	GU360688	GU360426	GU359425	GU359976	GU359152
106	*Muhlenbergiaalopecuroides* (Griseb.) P.M. Peterson & Columbus	Peterson 22008 & Saarela (US)	Mexico, Chihuahua	GU360223	GU360687	GU360425	GU359451	GU359975	GU359153
107	*Muhlenbergiaalopecuroides* (Griseb.) P.M. Peterson & Columbus	Peterson 24524 & Romaschenko (US)	Mexico, Coahuila	**MK090798**	**MK090858**	**MK090831**	**MK090711**	**MK090747**	**MK090660**
108	*Muhlenbergiaalopecuroides* (Griseb.) P.M. Peterson & Columbus	Peterson 24885 & Romaschenko (US)	Mexico, San Luis Potosí	**MK090799**	**MK090859**	**MK090832**	**MK090712**	**MK090748**	**MK090661**
109	*Muhlenbergiaarenacea* (Buckley) Hitchc.	Peterson 10624 & Annable (US)	Mexico, Coahuila	GU360210	GU360675	GU360414	GU359452	GU359961	GU359165
110	*Muhlenbergiaasperifolia* (Nees & Meyen ex Trin.) Parodi	Peterson 15452, Soreng, Finot & Judziewicz (US)	Chile, Region III (Atacama)	HM143252	HM143634	HM143539	HM143356	HM143149	HM143048
111	*Muhlenbergiaatacamensis* Parodi	Peterson 19626, Soreng, Salariato, & Panizza, (US)	Argentina, Jujuy	GU360115	GU360595	GU360489	GU359382	GU359879	GU359344
112	*Muhlenbergiacuspidata* (Torr. ex Hook.) Rydb.	Hill 35331 (US)	USA	HM143260	−	HM143546	−	HM143156	HM143055
113	*Muhlenbergiafastigiata* (J. Presl) Henrard	Peterson 21512, Soreng, LaTorre & Rojas Fox (US)	Peru, Ancash	HM143270	HM143651	HM143556	HM143372	HM143166	HM143064
114	*Muhlenbergiaimplicata* (Kunth) Trin.	Peterson 22266, Saarela (US)	Mexico, Oaxaca	HM143283	HM143664	HM143568	HM143385	HM143179	HM143077
115	*Muhlenbergiajaime-hintonii* P.M. Peterson & Valdés-Reyna	Peterson 15841 & Valdes Reyna (US)	Mexico, Nuevo León	HM143285	HM143665	HM143569	HM143386	HM143181	HM143079
116	*Muhlenbergialigulata* (E. Fourn.) Scribn. & Merr.	Peterson 22416 & Saarela (US)	Mexico, Durango	GU360069	GU360551	GU360440	GU359381	GU359863	GU359273
117	*Muhlenbergiamonandra* Alegría & Rúgolo	Peterson 17990 & Refulio Rodriguez (US)	Peru, Lima	−	−	−	−	−	GQ397891*
118	*Muhlenbergiamultiflora* Columbus	Peterson 7845 & Annable (US)	USA, Colorado	GU360191	GU360702	GU360289	GU359525	GU359985	GU359138
119	*Muhlenbergiapalmirensis* Grignon & Lægaard	Peterson 9317 & Judziewicz (US)	Ecuador, Chimborazo	HM143305	HM143685	HM143586	HM143402	HM143200	HM143098
120	*Muhlenbergiapaniculata* (Nutt.) Columbus	Peterson 12070 & Annable (US)	USA, Colorado	GU360170	GU360673	GU360375	GU359529	GU359936	GU359201
121	*Muhlenbergiapaniculata* (Nutt.) Columbus	Stevens 909 (US)	USA	**MK090814**	**MK090874**	**MK090847**	**MK090726**	**MK090766**	−
122	*Muhlenbergiaphleoides* (Kunth) Columbus	Peterson 24452, Romaschenko & Valdés-Reyna (US)	Mexico, Nuevo León	−	−	−	−	MH400231	MH400228
123	*Muhlenbergiaphleoides* (Kunth) Columbus	Peterson 24668 & Romaschenko (US)	Mexico, San Luis Potosí	−	−	−	−	**MK090770**	**MK090683**
124	*Muhlenbergiaphleoides* (Kunth) Columbus	Peterson 24717, Romaschenko & Zamudio Ruíz (US)	Mexico, Querétaro	−	−	−	−	**MK090771**	**MK090684**
125	*Muhlenbergiaphleoides* (Kunth) Columbus	Peterson 24731, Romaschenko & Zamudio Ruíz (US)	Mexico, Querétaro	−	−	−	−	**MK090772**	**MK090685**
126	*Muhlenbergiaphleoides* (Kunth) Columbus	Peterson 24799, Romaschenko, Rodriguez Avalos, Herrera-Simoni, & Garcia Rodriguez (US)	Mexico, Aguascalientes	−	−	−	−	MH400232	MH400229
127	*Muhlenbergiaphleoides* (Kunth) Columbus	Peterson 24938, Romaschenko & González-Elizondo (US)	Mexico, Durango	−	−	−	−	KX582659	KX582383
128	*Muhlenbergiapungens* Thurb. ex A. Gray	Ricketson 4642 (MO)	USA, Arizona	**MK090815**	**MK090875**	MH508106	−	MH508102	MH508098
129	*Muhlenbergiarepens* (J. Presl) Hitchc.	Peterson 7900 & Annable (US)	USA, New Mexico	HM143316	HM143697	HM143596	HM143413	HM143212	HM143110
130	*Muhlenbergiarepens* (J. Presl) Hitchc.	Peterson 5735 & Annable (US)	USA, Texas	HM143338	HM143717	HM143617	HM143430	HM143233	HM143131
131	*Muhlenbergiarichardsonis* (Trin.) Rydb.	Peterson 19817, Saarela & Sears (US)	USA, California	GU360212	GU360677	GU360431	GU359454	GU359978	GU359163
132	*Muhlenbergiaseatonii* Scribn.	Peterson 9946 & Annable (US)	Mexico, Puebla	**MK090822**	**MK090882**	MH508107	**MK090735**	MH508103	MH508099
133	*Muhlenbergiaspatha* Columbus	Schaffner 134 (US)	Mexico	−	−	−	−	GU359981	MH400230
134	*Muhlenbergiasubbiflora* Hitchc.	Peterson 21158, Saarela, Rosen & Reid (US)	Mexico, Durango	GU360036	GU360518	GU360439	GU359428	GU359877	GU359318
135	*Muhlenbergiatenuissima* (J. Presl) Kunth	Peterson 4751 & Annable (US)	Mexico, Jalisco	**MK090829**	**MK090890**	MH508108	**MK090743**	MH508104	MH508100
136	*Muhlenbergiauniflora* (Muhl.) Fernald	Peterson 13212, Annable, Pizzolato, Gordon, Frett, Frick, Morrone & Griner (US)	USA, New Jersey	HM143337	HM143716	HM143616	HM143429	HM143232	HM143130
137	*Muhlenbergiauniflora* (Muhl.) Fernald	Peterson 20862 & Saarela (US)	USA, New York	GU360258	GU360715	GU360275	GU359463	GU359994	GU359119
138	*Muhlenbergiautilis* (Torr.) Hitchc.	Peterson 24869 & Romaschenko (US)	Mexico, San Luis Potosí	−	−	−	−	MH508105	MH508101
139	*Muhlenbergiavilliflora* Hitchc.	Peterson 15811 & Valdes Reyna (US)	Mexico, Nuevo León	HM143340	HM143720	HM143620	HM143431	HM143236	HM143133
140	*Muhlenbergiawrightii* Vasey ex J.M. Coult.	Peterson 20964, Saarela, Lara Contreras & Reyna Alvarez (US)	Mexico, Coahuila	HM143344	HM143723	HM143623	HM143434	HM143240	HM143137
subg. Trichochloa									
141	*Muhlenbergiaarticulata* Scribn.	Peterson 13386 & Knowles (US)	Mexico, San Luis Potosi	HM143251	HM143633	HM143538	HM143355	HM143148	HM143047
142	*Muhlenbergiabreviligula* Hitchc.	Pohl 13392 & Gabel (MO)	Honduras, Santa Bárbara	**MK090800**	**MK090860**	**MK090833**	−	**MK090749**	**MK090662**
143	*Muhlenbergiacapillaris* (Lam.) Trin.	Peterson 14236, Weakley & LeBlond (US)	USA, North Carolina	HM143255	HM143638	HM143542	HM143359	HM143152	HM143051
144	*Muhlenbergiadistichophylla* (J. Presl) Kunth	Peterson 15913 & Valdes Reyna (US)	Mexico, Tamaulipas	HM143262	HM143643	HM143548	HM143364	HM143158	−
145	*Muhlenbergiacoerulea* (Griseb.) Mez	Peterson 21703, Soreng, LaTorre & Rojas Fox (US)	Peru, Ancash	HM143245	HM143627	HM143532	HM143349	HM143142	HM143041
146	*Muhlenbergiacoerulea* (Griseb.) Mez	Peterson 21958, Soreng & Montoya Quino (US)	Peru, La Libertad	HM143246	HM143628	HM143533	HM143350	HM143143	HM143042
147	*Muhlenbergiadubia* E. Fourn.	Peterson 21105 & Saarela (US)	Mexico, Nuevo León	HM143263	HM143644	HM143550	HM143365	HM143160	HM143057
148	*Muhlenbergiadurangensis* Y. Herrera	Peterson 13644, Knowles, Dietrich, Braxton & Gonzalez-Elizondo (US)	Mexico, Durango	HM143266	HM143647	HM143552	HM143368	HM143162	HM143060
149	*Muhlenbergiaelongata* Scribn. ex Beal	Peterson 22164 & Saarela (US)	Mexico, Chihuahua	HM143267	HM143648	HM143553	HM143369	HM143163	HM143061
150	*Muhlenbergiaemersleyi* Vasey	Peterson 22096 & Saarela (US)	Mexico, Chihuahua	GU360207	GU360672	GU360411	GU359449	GU359958	GU359168
151	*Muhlenbergiaexpansa* (Poir.) Trin.	Peterson 14234, Weakley & LeBlond (US)	USA, North Carolina	HM143269	HM143650	HM143555	HM143371	HM143165	HM143063
152	*Muhlenbergiagigantea* (E. Fourn.) Hitchc.	Peterson 22260 & Saarela (US)	Mexico, Oaxaca	GU360215	GU360680	GU360419	GU359457	GU359966	GU359160
153	*Muhlenbergiagrandis* Vasey	Peterson 13413, Knowles, Dietrich & Braxton (US)	Mexico, Sinaloa	HM143279	HM143660	HM143565	HM143381	HM143175	HM143073
154	*Muhlenbergiagypsophila* Reeder & C. Reeder	Peterson 15840 & Valdes Reyna (US)	Mexico, Nuevo León	HM143280	HM143661	−	HM143382	HM143176	HM143074
155	*Muhlenbergiairidifolia* Soderstr.	Peterson 6133 & Annable (US)	Mexico, Jalisco	HM143284	−	−	−	HM143180	HM143078
156	*Muhlenbergiajaliscana* Swallen	Peterson 6149 & Annable (US)	Mexico, Jalisco	HM143286	HM143666	HM143570	HM143387	HM143182	HM143080
157	*Muhlenbergialehmanniana* Henrard	Santamaria 3760 (MO)	Colombia	−	−	−	−	−	**MK090673**
158	*Muhlenbergialindheimeri* Hitchc.	Peterson 6280 & Annable (US)	USA, Texas	HM143290	HM143670	HM143573	HM143391	HM143186	HM143084
159	*Muhlenbergialongiglumis* Vasey	Peterson 13666, Knowles, Dietrich, Braxton & Gonzalez Elizondo (US)	Mexico, Durango	HM143291	HM143671	−	−	−	−
160	*Muhlenbergialongiligula* Hitchc.	Peterson 15224 & Cayouette (US)	USA, Arizona	HM143292	HM143672	HM143574	**MK090724**	HM143187	HM143085
161	*Muhlenbergialucida* Swallen	Peterson 22134 & Saarela (US)	Mexico, Chihuahua	HM143294	HM143674	HM143575	HM143392	HM143188	HM143086
162	*Muhlenbergiamacroura* (Kunth) Hitchc.	Peterson 22062 & Saarela (US)	Mexico, Chihuahua	GU360265	GU360683	GU360409	GU359447	GU359956	GU359125
163	*Muhlenbergiamaxima* Lægaard & Sánchez Vega	Peterson 21884, Soreng & Sanchez Vega (US)	Peru, Cajamarca	HM143296	HM143676	HM143578	**MK090725**	HM143191	HM143089
164	*Muhlenbergiamucronata* (Kunth) Trin.	Peterson 22038 & Saarela (US)	Mexico, Chihuahua	HM143302	HM143682	HM143583	HM143399	HM143197	HM143095
165	*Muhlenbergianigra* Hitchc.	Peterson 16097 & Rosales (US)	Mexico, Jalisco	HM143303	HM143683	HM143584	HM143400	HM143198	HM143096
166	*Muhlenbergiapalmeri* Vasey	Peterson 5478 & Annable (US)	USA, Arizona	HM143304	HM143684	HM143585	HM143401	HM143199	HM143097
167	*Muhlenbergiapubescens* (Kunth) Hitchc.	Peterson 21250 & Saarela (US)	Mexico, Durango	HM143310	HM143691	HM143592	HM143406	HM143205	HM143103
168	*Muhlenbergiapubigluma* Swallen	Peterson 15838 & Valdes Reyna (US)	Mexico, Nuevo León	HM143311	HM143692	HM143593	HM143408	HM143207	HM143105
169	*Muhlenbergiapurpusii* Mez	Peterson 6227 & Annable (US)	Mexico, San Luis Potosi	HM143312	HM143693	HM143594	HM143409	HM143208	HM143106
170	*Muhlenbergiareederorum* Soderstr.	Peterson 21262 & Saarela (US)	Mexico, Durango	HM143315	HM143696	−	HM143412	HM143211	HM143109
171	*Muhlenbergiareverchonii* Vasey & Scribn.	Peterson 6285 & Annable (US)	USA, Texas	HM143317	HM143698	HM143597	HM143414	HM143213	HM143111
172	*Muhlenbergiarigens* (Benth.) Hitchc.	Peterson 22129 & Saarela (US)	Mexico, Chihuahua	GU360256	GU360729	GU360357	GU359481	GU359951	GU359117
173	*Muhlenbergiarigida* (Kunth) Kunth	Peterson 21637, Soreng, LaTorre & Rojas Fox (US)	Peru, Ancash	GU360255	GU360718	GU360405	GU359380	GU359952	GU359116
174	*Muhlenbergiarigida* (Kunth) Kunth	Peterson 22061 & Saarela (US)	Mexico, Chihuahua	HM143319	HM143700	HM143599	HM143416	HM143215	HM143113
175	*Muhlenbergiarigida* (Kunth) Kunth	Peterson 24538 & Romaschenko (US)	Mexico, Coahuila	**MK090818**	**MK090878**	**MK090850**	**MK090730**	**MK090775**	**MK090688**
176	*Muhlenbergiarigida* (Kunth) Kunth	Peterson 24576, Romaschenko & Valdés-Reyna (US)	Mexico, Coahuila	**MK090819**	**MK090879**	**MK090851**	**MK090731**	**MK090776**	**MK090689**
177	*Muhlenbergiarigida* (Kunth) Kunth	Peterson 24715, Romaschenko & Zamudio Ruíz (US)	Mexico, Queretaro	−	−	−	−	**MK090777**	**MK090690**
178	*Muhlenbergiarigida* (Kunth) Kunth	Peterson 24733, Romaschenko & Zamudio Ruíz (US)	Mexico, Queretaro	−	−	−	−	**MK090778**	**MK090691**
179	*Muhlenbergiarigida* (Kunth) Kunth	Peterson 24802, Romaschenko, Rodriguez Avalos, Herrera-Simoni & Garcia Rodriguez (US)	Mexico, Aguascalientes	**MK090820**	**MK090880**	**MK090852**	**MK090732**	**MK090779**	**MK090692**
180	*Muhlenbergiarigida* (Kunth) Kunth	Peterson 24805, Romaschenko, Rodriguez Avalos, Herrera-Simoni & Garcia Rodriguez (US)	Mexico, Aguascalientes	−	−	−	−	**MK090780**	**MK090693**
181	*Muhlenbergiarigida* (Kunth) Kunth	Peterson 24931, Romaschenko & González-Elizondo (US)	Mexico, Durango	−	−	−	−	**MK090781**	**MK090694**
182	*Muhlenbergiarigida* (Kunth) Kunth	Peterson 24932, Romaschenko & González-Elizondo (US)	Mexico, Durango	−	−	−	−	**MK090782**	**MK090695**
183	*Muhlenbergiarigida* (Kunth) Kunth	Peterson 24933, Romaschenko & González-Elizondo (US)	Mexico, Durango	**MK090821**	**MK090881**	−	**MK090733**	**MK090783**	**MK090696**
184	*Muhlenbergiarobusta* (E. Fourn.) Hitchc.	Peterson 15928 & Valdes Reyna (US)	Mexico, Nuevo Leon	HM143320	HM143701	HM143600	**MK090734**	HM143216	HM143114
185	*Muhlenbergiasericea* (Michx.) P.M. Peterson	Peterson 14843, Blackburn & Peterson (US)	USA, South Carolina	HM143321	HM143702	HM143601	HM143417	HM143217	HM143115
186	*Muhlenbergiasetifolia* Vasey	Peterson 20942, Saarela, Lara Contreras & Reyna Alvarez (US)	Mexico, Coahuila	HM143322	HM143703	HM143602	HM143418	HM143218	HM143116
187	*Muhlenbergiaspeciosa* Vasey	Peterson 13616, Knowles, Dietrich, Braxton, & Gonzalez Elizondo (US)	Mexico, Durango	HM143325	HM143705	HM143605	HM143421	HM143221	HM143119
188	*Muhlenbergiastricta* (J. Presl) Kunth	Peterson 13709 (US)	Mexico, Jalisco	HM143328	HM143708	HM143608	HM143423	HM143224	HM143122
189	*Muhlenbergiasubaristata* Swallen	Peterson 21243 & Saarela (US)	Mexico, Durango	HM143329	HM143709	HM143609	HM143424	HM143225	HM143123
190	*Muhlenbergiavenezuelae* Luces	Briceño 266 & Adamo (MO)	Venezuela, Merida	**MK090830**	**MK090891**	**MK090856**	**MK090746**	**MK090796**	**MK090709**
191	*Muhlenbergiaversicolor* Swallen	Peterson 9913 & Annable (US)	Mexico, Oaxaca	HM143339	HM143719	HM143619	−	HM143235	HM143132
192	*Muhlenbergiavirletii* (E. Fourn.) Soderstr.	Peterson 9724 & Campos Villanueva (US)	Mexico, Oaxaca	HM143343	**MK090892**	**MK090857**	−	HM143239	HM143136
193	*Muhlenbergiaxinvoluta* Swallen	Peterson 6284 & Annable (US)	USA, Texas	HM143345	HM143724	HM143624	HM143435	HM143241	HM143138

### Taxonomy

Specimens of *Muhlenbergia* from Peru were reviewed in the following herbaria: AAU, COL, CPUN, CUZ, F, HAO, K, LIL, LPB, MO, MOL, NY, US and USM. The first author (PMP) travelled and collected 3042 numbers (primarily grasses) while on seven expeditions to Peru between 1997 and 2008. Of these, 258 are collections of *Muhlenbergia* and all are accounted for here. Synonymy is mainly limited to names of taxa described from South America. Additional synonyms accepted by us can be found in the Catalogue of New World Grasses, vol. II (Peterson et al. 2001) and on the Catalogue of New World Grasses web site (http://www.tropicos.org/Project/CNWG) that is continually updated within TROPICOS (http://www.tropicos.org). When counting culm nodes, it is best to start counting 1 cm above the base. Blade width is measured from margin to margin on a flat blade but not when the blade is tightly involute. Glabrous refers to without pubescence. Smooth indicates no prickle-hairs with broad bases and/or hooked or pointed apices (i.e. pubescence can occur on a smooth surface and a scabrous surface can be glabrous). Excluded species and an infrageneric classification of the accepted species of *Muhlenbergia* in Peru are presented at the end of the taxonomic treatment.

## Results and discussion

### Phylogeny

A total of 226 new sequences from 146 species of *Muhlenbergia* are newly reported in GenBank (Table 1). The following 13 species are new in the phylogeny of *Muhlenbergia*: *M.capillipes* (M.E. Jones) P.M. Peterson & Annable, *M.ligularis* (Hack.) Hitchc., *M.flabellata* Mez, *M.bushii* R.W. Pohl, *M.diversiglumis* Trin., *M.frondosa* (Poir.) Fernald, *M.glabrifloris* Scribn., *M.romaschenkoi* P.M. Peterson, *M.setaroides* E. Fourn., *M.sylvatica* (Torr.) Torr. ex A Gray, *M.breviligula* Hitchc., *M.lehmanniana* Henrard and *M.venezuelae* Luces (Peterson et al. 2010b; 2018). Total aligned characters for individual regions and other parameters are noted in Table 2. The plastid−ITS sequences were combined in the analysis except where incongruence between datasets was detected (Fig. 1).

**Table 2.** Characteristics of the six regions, *rps3*, *rps16-trnK*, *rps16 intron*, *ndhA intron*, *rpl32-trnL*, *and ITS*, and parameters used in Bayesian analyses indicated by Akaike Information Criterion (AIC).

	***rps3***	***rps16-trnK***	***rps16 intron***	***ndhA intron***	***rpl32-trnL***	***ITS***	**Overall**
Total aligned characters	615	1000	1088	1189	996	761	5649
Number of new sequences	31 (19.5%)	33 (20.8%)	26 (17.2%)	34 (23%)	48 (25.9%)	50 (26.7%)	222 (22.4%)
Likelihood score (-lnL)	2342.85	3659.76	3429.94	5167.05	4762.52	9632.65	
Number of substitution types	6	6	6	6	6	6	−
Model for among-site rate variation	gamma	gamma	gamma	gamma	gamma	gamma	−
Substitution rates							
rAC	0.90029	1.07678	1.23238	0.75118	1.09619	1.43396	−
rAG	1.92233	3.01634	1.29662	2.20104	1.87400	2.69229	
rAT	0.56014	0.53859	0.46844	0.62199	0.45930	1.81818	
rCG	1.67415	1.17251	1.02428	1.66881	1.25539	0.71134	
rCT	3.50831	2.22649	2.45667	2.58721	1.47927	5.08729	
rGT	1.00000	1.00000	1.00000	1.00000	1.00000	1.00000	
Character state frequencies							
fA	0.43511	0.31626	0.38612	0.38183	0.38425	0.26145	−
fC	0.14841	0.12963	0.11052	0.11648	0.11939	0.19766	
fG	0.16534	0.13166	0.17725	0.13533	0.12321	0.24978	
fT	0.25114	0.42244	0.32611	0.36637	0.37315	0.29111	
Proportion of invariable sites	0.19372	0.10382	0.38367	0.07818	0.1631	0.38392	−
Gamma shape parameter (α)	0.74961	1.26136	0.92705	0.96285	0.93242	0.80871	−

The Bayesian tree from the combined analysis of ITS and five plastid regions (*ndhA* intron, *rpl32-trnL, rps3, rps16* intron and *rps16-trnK*) is well resolved with strong support for the monophyly of *Muhlenbergia*, including *M.ramulosa* (Kunth) Swallen, sister to M.subg.Bealia−M.subg.Trichochloa, these all being in one clade that is sister to M.subg.Clomena−M.subg.Pseudosporobolus and sister to M.subg.Muhlenbergia. (Fig. 1; posterior probability, PP = 0.95−1, shown with thick branches). Each of the five major clades, corresponding to the five subgeneric divisions that have been recognised previously (Peterson et al. 2010b), include species that occur in Peru and/or South America (red) or as wide ranging species from the Americas. The species in each of these five subgenera share salient morphological characteristics or trends.

Species of M.subg.Bealia are strongly caespitose, never rhizomatous, annuals or perennials with pubescent margins or midnerves at least on the lower ½ of the lemma (only *M.ligularis* is without pubescence) and round, equal primary, secondary and tertiary vascular bundles without well-developed schlerenchyma (Peterson and Herrera Arrieta 2001; Peterson 2003; Peterson et al. 2010b). The Peruvian endemic, *M.caxamarcensis* Lægaard & Sánchez Vega and the wide-ranging, *M.ligularis* (Central and South America), are members of this subgenus.

Although the clade of species representing the M.subg.Trichochloa is strongly supported in our analyses (Fig. 1), there is little resolution among members, suggesting very low levels of genetic divergence among the species in this subgenus. The low level of divergence may be a consequence of rapid speciation events. Within *Muhlenbergia*, this group is by far the most difficult to determine because there are very few morphological differences among the taxa and discrete (nonplastic) characteristics are few. The species of M.subg.Trichochloa consist of robust perennials up to 3 metres tall with compressed-keeled or rounded basal sheaths, 1-veined glumes and unequal rectangular or obovate/elliptic secondary and tertiary vascular bundles with well-developed sclerenchyma girders, these usually with sclerosed phloem (Peterson and Herrera Arrieta 2001; Herrera Arrieta and Peterson 2007, 2017, 2018; Peterson et al. 2010b). In Peru, *M.coerulea* (Griseb.) Mez, *M.coerulea×M.rigida, M.maxima* Lægaard & Sánchez Vega (endemic) and *M.rigida* (Kunth) Kunth are placed in M.subg.Trichochloa.

Species of M.subg.Clomena have 3-veined upper glumes that are often 3-toothed, densely caespitose non-rhizomatous culms with lower leaf sheaths that are often flat and somewhat papery at maturity and lemmas with flexuous awns [only *M.jonesii* (Vasey) Hitchc. lacks an awn, apex is mucronate] (Reeder and Reeder 1995; Herrera Arrieta 1998; Peterson 2003; Peterson et al. 2010b). The wide-ranging *M.peruviana* (P. Beauv.) Steud. is the only species of this subgenus that reaches South America, including Peru.

Members of M.subg.Pseudosporobolus usually have plumbeous spikelets, well-developed adaxial and abaxial sclerenchyma in their primary vascular bundles, narrow to loosely open *panicles*, unawned, mucronate or short-awned lemmas [long-awned in *M.implicata* (Kunth) Trin. and *M.seatonii* Scribn.] and the plants are rhizomatous when perennial (Peterson and Herrera Arrieta 2001; Peterson 2003; Peterson et al. 2010b). In Peru, *M.fastigiata* (J. Presl) Henrard, *M.monandra* Alegría & Rúgolo (endemic) and *M.phalaroides* (Kunth) P.M. Peterson are placed in M.subg.Pseudosporobolus.

Morphologically, species of the M.subg.Muhlenbergia clade have broad, flat leaf blades, most have well-developed, scaly and creeping rhizomes and *panicles* that are usually narrow at maturity (Peterson et al. 2010b). This is the only subgenus where the PCK subtype of C_4_ photosynthesis has been found. PCK species contain chlorenchyma composed of tabular cells that are indistinctly radiate and continuous between bundles [PCK type, defined as centrifugal/evenly distributed photosynthetic carbon reduction (PCRD) cell chloroplasts (with grana). The major veins are surrounded by two bundle sheaths, an inner mestome sheath of elongate non-chlorenchymatous cells and an outer chlorenchymatous sheath of shorter PCRD cells (designated XyMS+structural type; Hattersley and Watson 1976, 1992; Dengler et al. 1986). In addition, the leaf blades of these species contain fan- to shield-shaped bulliform cells that do not form a column of colourless cells from the adaxial to the abaxial surface and they generally have four or more secondary and/or tertiary vascular bundles between consecutive primary vascular bundles (Gutierrez et al. 1974; Brown 1977; Peterson and Herrera Arrieta 2001). Anatomically, *Muhlenbergiatarahumara* P.M. Peterson & Columbus is an exception to the standard PKC-type leaf structure in having non-contiguous chlorenchyma separated by columns of colourless cells between adjacent vascular bundles (Peterson and Columbus 2009; Peterson et al. 2010b). In Peru, *M.beyrichiana* Kunth, *M.bryophilus* (Döll) P.M. Peterson, *M.cenchroides* (Humb. & Bonpl. ex Willd.) P.M. Peterson, *M.ciliata* (Kunth) Trin., *M.diversiglumis, M.flexuosa* Hitchc. (endemic), *M.microsperma* (DC.) Kunth and *M.romaschenkoi* (endemic) are placed in M.subg.Muhlenbergia.

### Taxonomic treatment

#### Muhlenbergia

Taxon classificationPlantaePoalesPoaceae

Schreb., Gen. Pl. 1: 44. 1789.

Dilepyrum Michx., Fl. Bor.-Amer. 1: 40. 1803. Type: Dilepyrumminutiflorum Michx. (= Muhlenbergiaschreberi J. F. Gmel.).
Aegopogon Humb. & Bonpl. ex Willd., Sp. Pl. 4 (2): 899. 1805 [1806] Type: Aegopogoncenchroides Humb. & Bonpl. ex Willd. [≡ Muhlenbergiacenchroides (Humb. & Bonpl. ex Willd.) P.M. Peterson].
Podosemum Desv., Nouv. Bull. Sci. Soc. Philom. Paris 2: 188. 1810. Type: Podosemumcapillare (Lam.) Desv. [= Muhlenbergiacapillaris (Lam.) Trin.].
Clomena P. Beauv., Ess. Agrostogr. 28. 1812. Type: Clomenaperuviana P. Beauv. [≡ Muhlenbergiaperuviana (P. Beauv.) Steud.].
Tosagris P. Beauv., Ess. Agrostogr. 29. 1812. Type: Tosagrisagrostidea P. Beauv. [= Muhlenbergiacapillaris (Lam.) Trin.].
Trichochloa P. Beauv., Ess. Agrostogr. 29. 1812. Type: Trichochloapurpurea P. Beauv. [= Muhlenbergiaexpansa (Poir.) Trin.].
Podosaemum Kunth, Mem. Mus. Hist. Nat. 2: 72. 1815; orth. var. *Podosemum*.
Hymenothecium Lag., Gen. Sp. Pl. 4. 1816. Lectotype: Cynosurustenellus Cav. ex DC., designated by Hitchcock 1920: 169 [≡ Lamarckiatenella DC. ≡ Hymenotheciumtenellum (Cav. ex DC.) Lag. = Muhlenbergiauniseta (Lag.) Columbus].
Lycurus Kunth, Nov. Gen. Sp. 1: 141. 1815 [1816]. Lectotype: Lycurusphleoides Kunth, designated by Hitchcock 1920: 139 [≡ Muhlenbergiaphleoides (Kunth) Columbus].
Anthipsimus Raf., J. Phys. Chim. Hist. Nat. Arts 89: 105. 1819. Type: Anthipsimusgonopodus Raf. (= Muhlenbergiaschreberi J.F. Gmel.).
Sericrostis Raf., Neogenyton 4. 1825. Lectotype: Stipasericea Michx, designated by Pfeiffer 1874: 1142 [≡ Muhlenbergiasericea (Michx.) P.M. Peterson].
Pereilema J. Presl, Reliq. Haenk. 1 (4–5): 233.1830. Type: Pereilemacrinitum J. Presl (≡ Muhlenbergiapereilema P.M.Peterson).
Epicampes J. Presl, Reliq. Haenk. 1 (4–5): 235. 1830. Type: Epicampesstricta J. Presl [= Muhlenbergiarobusta (E. Fourn.) Hitchc.].
Dactylogramma Link, Hort. Berol. 2: 248. 1833. Type: Dactylogrammacinnoides Link [= Muhlenbergiaglomerata (Willd.) Trin.].
Calycodon Nutt., Proc. Acad. Nat. Sci. Philadelphia 4: 23. 1848. Type: Calycodonmontanum Nutt. [≡ Muhlenbergiamontana (Nutt.) Hitchc.].
Pleopogon Nutt., Proc. Acad. Nat. Sci. Philadelphia 4: 25. 1848. Type: Pleopogonsetosum Nutt. [≡ Lycurussetosus (Nutt.) C.G. Reeder = Muhlenbergiaalopecuroides (Griseb.) P.M. Peterson & Columbus].
Schedonnardus Steud., Syn. Pl. Glumac. 1: 146. 1854. Type: Schedonnardustexanus Steud. [Spirochloepaniculata Nutt. = Schedonnarduspaniculatus (Nutt.) Branner & Coville = Muhlenbergiapaniculata (Nutt.) Columbus].
Vaseya Thurb., Proc. Acad. Nat. Sci. Philadelphia 15: 79. 1863. Type: Vaseyacomata Thurb. [= Muhlenbergiaandina (Nutt.) Hitchc.].
Chaboissaea E. Fourn., Mexic. Pl. 2: 112. 1886. Type: Chaboissaealigulata E. Fourn. [≡ Muhlenbergialigulata (E. Fourn.) Scribn. & Merr.].
Crypsinna E. Fourn., Mexic. Pl. 2: 90. 1886. Lectotype: Crypsismacroura Kunth, designated by Hitchcock 1920: 144. [≡ Crypsinnamacroura (Kunth) E. Fourn.≡ Muhlenbergiamacroura (Kunth) Hitchc.].
Redfieldia Vasey, Bull. Torrey Bot. Club 14: 133. 1887. Type: Graphephorumflexuosum Thurb. ex A. Gray [≡ Redfieldiaflexuosa (Thurb. ex A. Gray) Vasey ≡ Muhlenbergiamultiflora Columbus].
Bealia Scribn., True Grasses 104, f. 45a. 1890. Type: Bealiamexicana Scribn. (≡ Muhlenbergiabiloba Hitchc.).
Blepharoneuron Nash, Bull. Torrey Bot. Club 25(2): 88. 1898. Lectotype: Vilfatricholepis Torr., designated by Peterson and Annable 1990: 522 [≡ Blepharoneurontricholepis (Torr.) Nash ≡ Muhlenbergiatricholepis (Torr.) Columbus].
Schaffnerella Nash, N. Amer. Fl. 17(2): 141. 1912. Type: Schaffneragracilis Benth. [≡ Schaffnerellagracilis (Benth.) Nash ≡ Muhlenbergiaspatha Columbus].

##### Description.

Plants *annual* or *perennial*; synoecious sometimes andromonoecious; sometimes rhizomatous, often cespitose, sometimes mat-forming, rarely stoloniferous. *Culms* 2–300 cm, erect, geniculate or decumbent, usually herbaceous, sometimes becoming woody. *Sheaths* open, overlapping below; *ligules* membranous or hyaline (rarely firm or coriaceous), acuminate to truncate, sometimes minutely ciliolate, sometimes with lateral lobes longer than the central portion; *blades* narrow, flat, folded, or involute, sometimes arcuate. *Inflorescences* terminal, sometimes also axillary, open to contracted, raceme-like or spike-like *panicles*; *cleistogamous panicles* sometimes present in the axils of the lower cauline leaves, enclosed by a tightly rolled, somewhat indurate sheath; *disarticulation* usually above the glumes, occasionally below the pedicels. *Spikelets* mostly perfect with 1 (2–6) florets, sometimes staminate or sterile, occasionally paired or in groups of threes then the central spikelet perfect and the lateral ones staminate or sterile; chasmogamous, rarely cleistogamous; *glumes* usually (0)1(2–3)-veined, apices entire, erose or toothed, truncate to acuminate, sometimes mucronate or awned from the midvein, occasionally awned from the lateral veins; *lower glumes* sometimes rudimentary or absent, occasionally bifid; *upper glumes* shorter than to longer than the florets; *calluses* poorly developed, glabrous or with a few hairs; *lemmas* glabrous, scabrous or with short hairs, 3-veined (rarely appearing 5-veined), apices awned from the midvein, mucronate or unawned; *awns*, if present, straight, flexuous, sinuous or curled, sometimes borne between 2 minute teeth, lateral veins occasionally extended into awns; *paleas* shorter than or equal to the lemmas, 2-veined, apices; *anthers* (1–2)3, purple, orange, yellow, olivaceous or whitish; *ovary* with 2 styles, stigmas plumose. *Caryopses* elongate, fusiform or elliptic, slightly dorsally compressed, rarely laterally compressed, glabrous; *hilum* short; *pericarp* fused.

Chromosome base number is × = (8 or 9) 10 and these are relatively small in size.

##### Distribution.

The genus is primarily distributed in the Western Hemisphere in North Central and South America. There are also seven species known to occur in south-eastern Asia, six of these are found in China (Wu and Peterson 2006).

##### Ecology.

The species occur in open habitats in deserts, grasslands, sclerophllous scrubland and margins of forests often in xeric to meso-xeric habitats from near sea level to more than 4000 m.

##### Etymology.

Named for Gotthilf Heinrich Ernst Muhlenberg (1753–1815), a Lutheran minister and pioneering botanist of Pennsylvania, USA.

#### Key to the species of *Muhlenbergia* in Peru

**Table:** 

1	Spikelets grouped in fascicles of 2−4 spikelets	**2**
−	Spikelets not grouped in fascicles	**5**
2	Each fascicle subtended by 10−15 bristles, the bristles 1−5 mm long; ligules auriculate, the auricles 1−2 mm long, ciliate, sometimes cauducous at maturity, the cilia about 1 mm long	**1. *M.beyrichiana***
−	Fascicles never subtended by bristles; ligules not auriculate and not ciliate or hairy	**3**
3	Anthers 0.5−0.7 mm long; sprawling, slender annuals (4–) 6–30 cm long; sessile or inconspicuously pedicelled perfect spikelet associated with one (rarely two) staminate or sterile pedicelled spikelets	**2. *M.bryophilus***
−	Anthers 1.6−2 mm long; sprawling perennials, 10–55 cm long; sessile or inconspicuously pedicelled spikelet perfect and associated with two staminate or sterile pedicelled spikelets or the lower short-pedicelled spikelet perfect, staminate or sterile and the upper longer-pedicelled spikelet perfect, staminate or sterile	**4**
4	Apex of lemmas 3-awned, the central awns 5−13 mm long, lateral awns 2–3 mm long; lemmas 2.5–3 mm long with glabrous margins; lower glumes 1-veined, apex deeply notched with triangular lobes	**4. *M.cenchroides***
−	Apex of lemmas 1-awned, sometimes mucronate or unawned, the awns 1−3 mm long; lemmas 3−4 mm long with hirsute to lanate margins, the hairs 0.1−0.3 mm long; lower glumes commonly 2 or 3-veined, usually 2-awned (from lateral veins), apex not deeply notched and without triangular lobes	**16. *M.phalaroides***
5	Plants annual	**6**
−	Plants perennial	**13**
6	Upper glumes 3-veined, the apex broad and truncate, usually 2- or 3-toothed	**15. *M.peruviana***
−	Upper glumes 1-veined, the apex acute, acuminate, or subulate, not 2- or 3-toothed	**7**
7	Lemmas mucronate, the mucro usually less than 1 mm long or occasionally short-awned with the awn up to 1.5 mm long	**8**
−	Lemmas awned, the awns 1.2−30 mm long, at least some of the spikelets with awns much longer than 1.2 mm	**9**
8	Lemmas sericeous, lower ½−¾ with scattered hairs, the hairs 0.3−0.5 mm long; paleas hairy between the nerves	**3. *M.caxamarcensis***
−	Lemmas glabrous; paleas glabrous between the nerves	**11. *M.ligularis***
9	Florets with a single stamen; *panicles* 2−9 mm wide, tightly spiciform; lemma awns 1.2−5 mm long; both glumes as long or longer than the floret	**14. *M.monandra***
−	Florets with 3 stamens; *panicles* 1.8−13.5 cm wide, sometimes narrow or contracted when immature but with spreading or reflexed branches at maturity; lemma awns 5−30 mm long; both glumes not longer than the floret (lower glume longer in *M.diversiglumis*)	**10**
10	Cleistogamouspanicles with 1–3 spikelets present in the axils of the lower sheaths; glume apices obtuse, unawned	**13. *M.microsperma***
−	Cleistogamouspanicles not present in the axils of the lower sheaths; glume apices acute to acuminate and usually awned	**11**
11	Panicles secund; primary branches with 2–5 spikelets; secondary branches not developed; spikelets dimorphic with respect to the glumes, the glumes of the proximal spikelet on each branch subequal, to 0.7 mm long, orbicular and unawned, those of the distal spikelets evidently unequal, the lower glumes to 8 mm long and usually awned, the upper glumes orbicular, sometimes awned	**8. *M.diversiglumis***
−	Panicles not secund; primary branches always with more than 2 spikelets, usually with more than 5; secondary branches well-developed; spikelets monomorphic with respect to the glumes	**12**
12	Ligules 0.2−0.8 mm long, apex truncate, entire; paleas glabrous between the nerves on the proximal ½; 6−13 nodes along the panicle; *panicles* 4−12 cm long	**5. *M.ciliata***
−	Ligules 1.2−3.0 (−5.0) mm long, acute to obtuse, lacerate; paleas sparsely appressed pubescent between the nerves on the proximal ½; 15−23 nodes along the panicle axis; *panicles* 7−15 cm long	**18. *M.romaschenkoi***
13	Culms 40−140 cm tall; leaf blades 12−45 cm long	**14**
−	Culms 2−40 cm tall; leaf blades 0.3−12 cm long	**15**
14	Panicles contracted and spike-like, 0.6−2 cm wide, usually plumbeous to reddish-purple; panicle branches tightly ascending from the rachises at maturity	**6**
−	Panicles loosely contracted to loosely spreading, (2−) 3−6 (−15) cm wide; panicle branches ascending and/or spreading up to 80° from the rachises	**17**
16	Glumes (4−) 5−7 (−8) mm long, usually as long or longer than the floret; lemmas unawned, mucronate or short-awned, the awns 1−3(−4) mm long	**6. *M.coerulea***
−	Glumes 2.5−3.5 (−4) mm long, 1/2−3/4 as long as the floret; lemmas awned, the awns generally 3−8(−10) mm long	**7. *M.coerulea* × *M.rigida***
17	Lemmas 2.5−2.8 mm long with scattered sericeous hairs along the veins above the hairy callus; caryopses 1.4−1.7 mm long	**12. *M.maxima***
−	Lemmas 3.5−6 mm long, glabrous along the veins above the hairy callus; glumes caryopses 2−3.5 mm long	**18**
18	Lemmatal awns 3−8 (−10) mm long; glumes ½−¾ as long as the floret, 2.5−3.5 (−4) mm long; lemmas 4−6 mm long; spikelets plumbeous to reddish-purple	**7. *M.coerulea × M.rigida***
−	Lemmatal awns (8−) 10−22 mm long; glumes less than ½ as long as the floret, 1−1.7 (−2) mm long; lemmas 3.5−5 mm long; spikelets reddish-purple	**17. *M.rigida***
15	Lemmas awned, the awns 14−30 mm long	**19**
−	Lemmas unawned, mucronate, the mucro usually less than 1 mm long or occasionally short-awned with an awn up to 3 mm long	**20**
19	Lower glumes 2.8−4 mm long including the mucro or short-awn if present; ligules 0.3−0.5 mm long, apex truncate; anthers 1.3−1.6 mm long	**10. *M.flexuosa***
−	Lower glumes 1−2.5 mm long including the mucro if present; ligules 1.2−3 (−5.0) mm long, apex acute; anthers 0.3−0.4 mm long	**18. *M.romaschenkoi***
20	Lower glumes commonly 2 or 3-veined and awned from the lateral veins, the 2 awns 1−3 mm long; spikelets paired on a branch, the lower spikelet staminate or sterile and the upper perfect	**16. *M.phalaroides***
−	Lower glumes 1-veined and unawned; spikelets borne singly on a branch or, if in pairs, the upper and lower both perfect	**21**
21	Plants rhizomatous, the main root or rhizome 1−2 (−2.5) mm wide; leaf blades tightly involute, arcuate, apex often pungent, the blades 2−8.5 mm long long	**9. *M.fastigiata***
−	Plants not rhizomatous, the roots less than 0.8 mm wide; leaf blades usually flat, straight, not pungent, the blades 3−22 mm long	**22**
22	Lemmas sericeous, lower ½−¾ with scattered hairs, the hairs 0.3−0.5 mm long; paleas hairy between the veins	**3. *M.caxamarcensis***
−	Lemmas glabrous; paleas glabrous between the veins	**11. *M.ligularis***

#### 1. Muhlenbergia beyrichiana

Taxon classificationPlantaePoalesPoaceae

Kunth, Enum. Pl. 1: 200. 1833.

Fig. 2A−F

Pereilema beyrichianum (Kunth) Hitchc., Contr. U.S. Natl. Herb. 24(8): 385. 1927. Type: Brazil, Province de Saint-Paul, Voyage d’Auguste de Saint-Hilaire de 1816 a 1821, *Saint-Hilare Cat. B #1028* (lectotype, designated here: P-00751689!; isolectotype: US-1645654! ex P).

##### Description.

Caespitose *annuals. Culms* 30−80 cm tall, slender, terete, glabrous, often branching from the aereal nodes below, usually with stilt-roots from lower nodes. *Leaf sheaths* usually shorter than the internodes, furrowed, scabrous; *ligules* 0.5−0.7 mm long, membranous; *auricles* 1−2 mm long, ciliate, sometimes cauducous at maturity, the cilia about 1 mm long; *blades* 8−20 cm long, 3−6 (−8) mm wide, flat, scabrous, apex attenuate. *Panicles* 5−16 (−20) cm long, 1−2.5 cm wide, contracted, spike-like, interrupted, the branches widely spaced along the rachis; *primary branches* 1−3 cm long, appressed, ascending or divergent and spreading. *Spikelets* in dense clusters of 2−4 subtended by 10−15 bristles, the bristles 1−5 mm long; *glumes* 0.7−1 mm long, ovate, membranous, 1-veined, apex awned, bidentate, the awns 3.5−5 mm long; *lemmas* (1.2−) 2−2.2 mm long, lanceolate, 3-veined, cartilaginous, scaberulous, awned, the awns 10−16 (−20) mm long, straight; *callus* short, the hairs 0.2−0.4 mm long; *paleas* as long as the lemmas, 2-veined, these often extending into mucros 0.1−0.3 mm long; *stamens* 3, *anthers* 0.7−1 mm long, purplish. *Caryopses* 1−1.3 mm long, ellipsoid.

**Figure 2.**
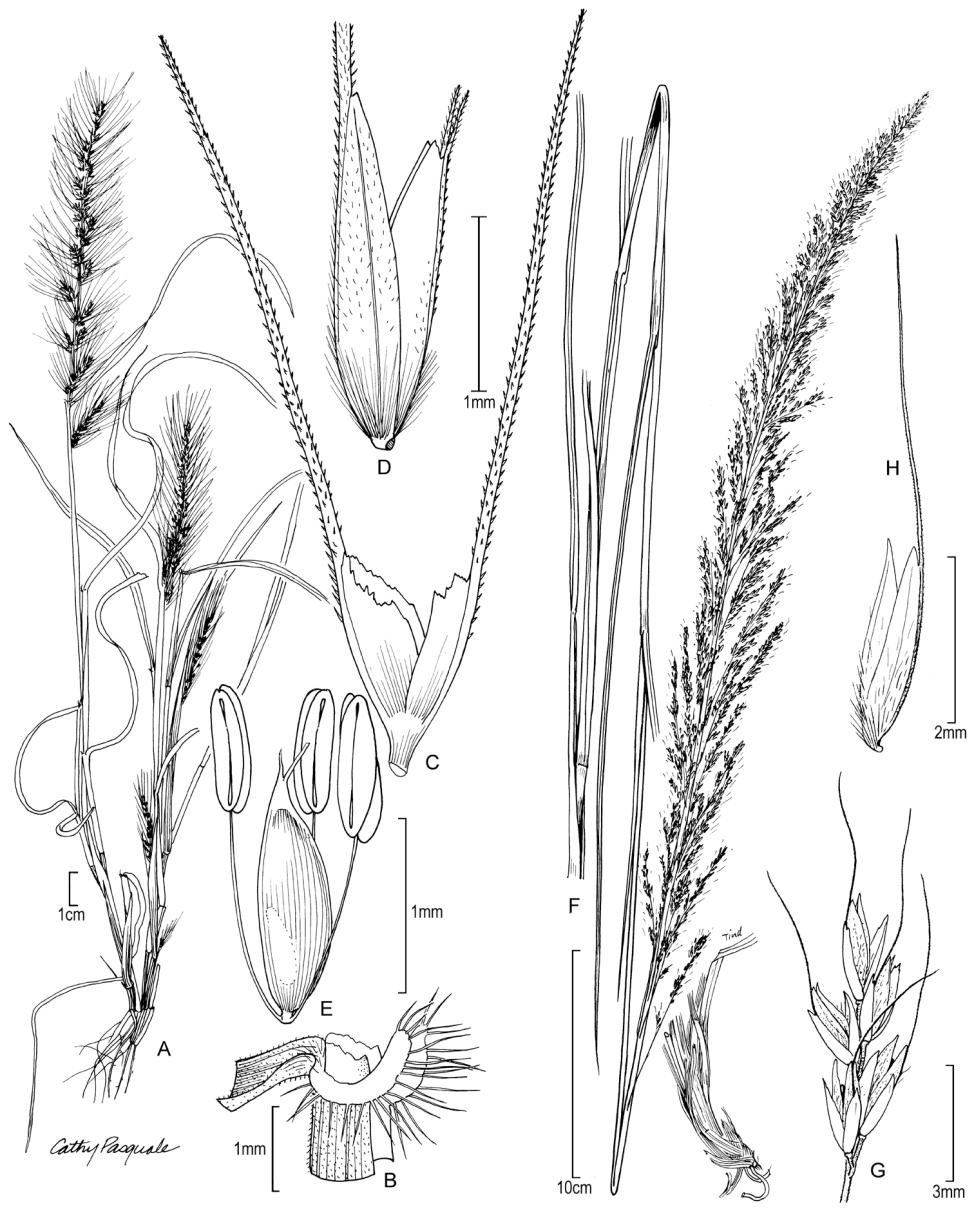
**A−E***Muhlenbergiabeyrichiana* Kunth **A** habit **B** ligule with auricles **C** glumes **D** floret **E** stamens and pistil **F−H***Muhlenbergiamaxima* Lægaard & Sánchez Vega **F** habit **G** end of panicle branch **H** floret. **A−E** drawn from *A.S. Hitchcock 20635* (US) **F−H** drawings from Lægaard & Sánchez Vega (1990) drawn from the holotype collection *Sánchez Vega & Ruíz Vigo 3561*.

##### Distribution.

*Muhlenbergiabeyrichiana* is known to occur in Brazil, Ecuador and Peru. Earlier, it was reported in Mexico and Central America but these are probably an error for *Muhlenbergiadiandra* (R.W. Pohl) Columbus, a more recently described species that superficially resembles *M.beyrichiana* (Espejo Serna et al. 2000; Lægaard and Peterson 2001; Peterson et al. 2001).

##### Ecology.

This species occurs in xerophilic savannahs with Cactaceae, roadsides and along moist ravines; 150−2000 m (Lægaard and Peterson 2001).

##### Comments.

*Muhlenbergiabeyrichiana* is morphologically similar to *M.pereilema* P.M. Peterson, which does not occur in Peru, but can be separated from the latter in having interrupted *panicles* with divergent primary branches (versus uninterruped *panicles* with tightly appressed primary branches in *M.pereilema*), straight lemma awns (versus flexuous or wavy), wide leaf blades 3−6 (−8) mm (versus 2−3 mm) and ciliate auricles (versus not ciliate) [Lægaard and Peterson 2001].

*Muhlenbergiabeyrichiana* is in a clade with *M.pereilema* and *M.plumiseta* and is sister to *M.flexuosa* Hitchc. in M.subg.Muhlenbergia (Fig. 1B; Peterson et al. 2010b).

##### Specimens examined.

Peru. **Junín**: Huancayo, abajo de Pariahuanca, 2000 m, 5 May 1979, Tovar 7880 (US, USM), Tovar 7901 (USM).

#### 2. Muhlenbergia bryophilus

Taxon classificationPlantaePoalesPoaceae

(Döll) P.M. Peterson, Caldasia 31(2): 279, f. 2 A–B, 2009.

Fig. 3A, B

Aegopogon bryophilus Döll, Fl. Bras. 2(3):239. 1880. Type: BRAZIL, Minas Gerais, 10 Apr 1879, *A.F.M. Glaziou 11661* (holotype: P-01863266 [image!]; isotypes: C-10016716 [image!], K-000309079 [image!], US-1280026!).
Aegopogon geminiflorus var. muticus Pilg., Bot. Jahrb. Syst. 27(1–2):25. 1899. Type: Bolivia, La Paz, near Sorata, 1 May 1892, *M. Bang 1307* (holotype: unknown; isotypes: CM-0179 [image!], K-000309080 [image!], NDG-07662 [image!], PUL-00000320 [image!], US-2473254!; W-18930005256 [image!]).
Aegopogon argentinus Mez, Repert. Spec. Nov. Regni Veg. 17(8−12):145. 1921. Type: ARGENTINA. Salta: Sierra Nevada del Castillo, *P.G. Lorentz & G. Hieronymus 182* (holotype: B?; isotypes: BAA-00001256 [image!], US-75037 fragm. ex B!).
Aegopogon fiebrigii Mez, Repert. Spec. Nov. Regni Veg. 17(8−12):145. 1921. Type: BOLIVIA. Camacho, *K. Fiebrig 2865* (holotype: B?; isotypes: MVFA-0000127 [image!], US-75308 fragm. ex B!).

##### Description.

Slender often sprawling, caespitose *annuals*. *Culms* (4–) 6–30 cm long, glabrous below the nodes; *internodes* 0.6–6 cm long, glabrous to pilose. *Leaf sheaths* mostly 0.5–4.8 cm long, shorter than the internodes, glabrous to sparingly pilose; *ligules* 0.6–1.5 mm long, glabrous, apex mostly truncate, lacerate, auricles absent; *blades* 1.5–6 cm long, 0.5–1.5(–1.7) mm wide, flat, scaberulent and pubescent above, mostly smooth beneath. *Panicles* 2–6 cm long, 0.5–1.2 cm wide, open, loosely-flowered; *primary branches* 0.2–0.6 mm long, excluding the awns, one per node, racemosely inserted. *Fertilespikelets* 2.4–3 mm long, in fascicles of two, rarely three per branch, often greenish or purplish, the clusters with one short-pedicelled spikelet (bisexual); *pedicels* (0.0−) 0.2–0.5 mm long (fertile spikelets) and the other two spikelets (sterile or staminate) longer pedicelled; *pedicels* about 0.7–1 mm long (sterile or staminate); *glumes* 1–1.3 mm long, narrowly acuminate, apex prolonged, aristate; *lemmas* 2.4–3 mm long, 3-awned, the central awns (3–) 5–8 (–12) mm long, lateral awns usually 0.8–1.4 mm long; *paleas* 2.2–2.8 mm long, puberulent, apex aristate, the awns usually 1–1.2 mm long; *anthers* 0.5–0.7 mm long, yellowish. *Caryopses* about 1 mm long, obovoid, light brownish.

**Figure 3.**
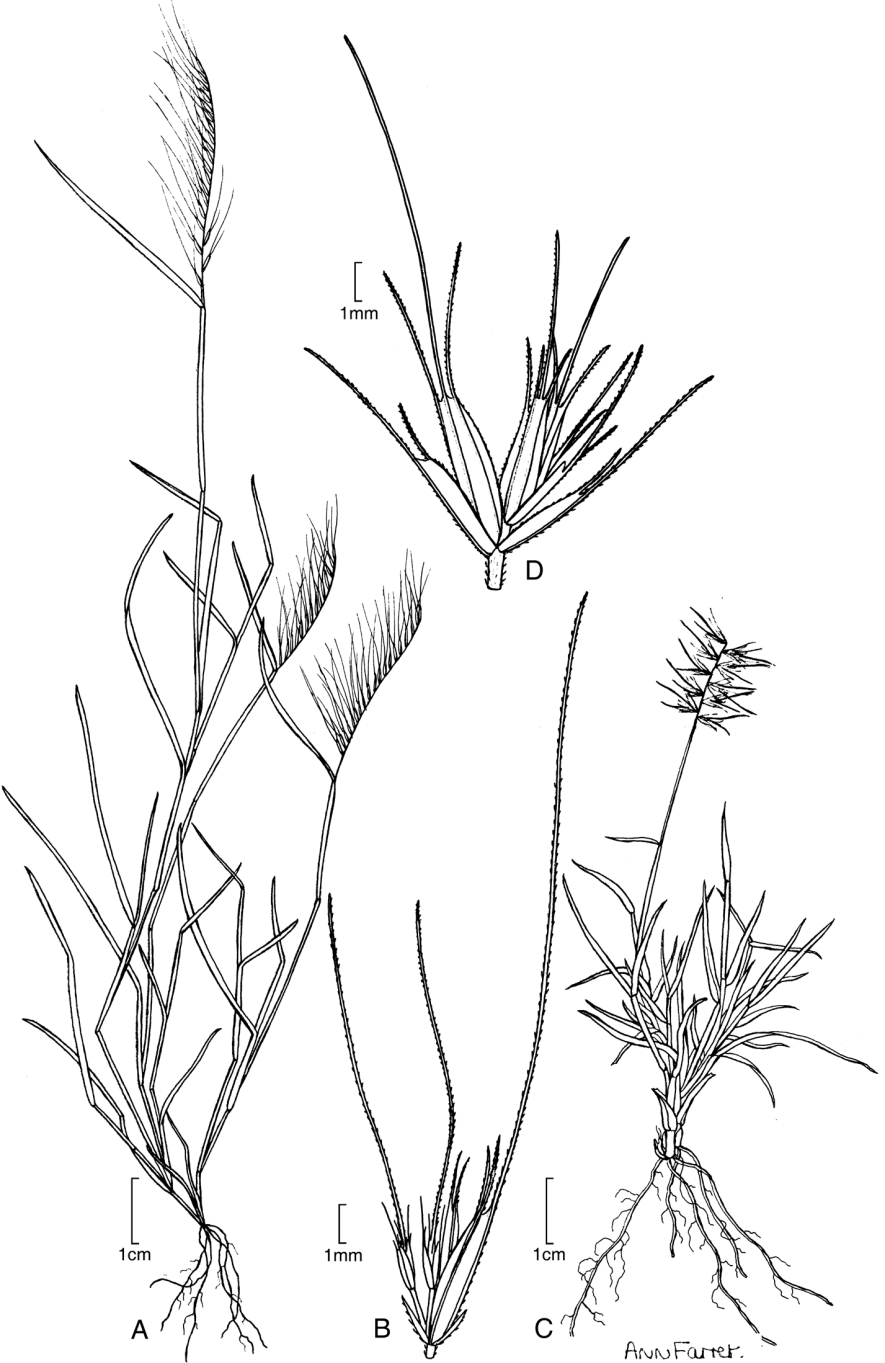
**A, B***Muhlenbergiabryophilus* (Döll) P. M. Peterson **A** habit **B** spikelet **C, D***Muhlenbergiacenchroides* (Humb. & Bonpl. ex Willd.) P. M. Peterson **C** habit **D** spikelet. Drawings from Giraldo-Cañas and Peterson (2009)**A, B** drawn from *S.G. Beck 818* (LPB) **C, D** drawn from *S.G. Beck 7464* (LPB).

##### Distribution.

*Muhlenbergiabryophilus* is found in South America, occuring in Argentina, Bolivia, Brazil, Chile, Colombia, Ecuador and Peru.

##### Ecology.

This species occurs on moist slopes, cliffs, barrancas, canyons, roadsides and along or near springs usually in shaded areas associated with *Cenchrusclandestinus*(Hochst. ex Chiov.) Morrone, *Boutelouasimplex* Lag., *Festucamyruos* L., *Urochloa*, *Veronica*, *Eragrostis*, *Baccharis*, *Salvia*, *Agave* and *Erodium*; 1500–3700 m. Flowering December through May.

##### Comments.

*Muhlenbergiabryophilus* is morphologically similar to *M.cenchroides* and can be separated from the latter in having anthers 0.5−0.7 mm long, the annual habit with culms (4–) 6–30 cm tall and sessile or inconspicuously pedicelled perfect spikelet associated with one (rarely two) staminate or sterile pedicelled spikelet (Tovar 1993; Giraldo-Cañas and Peterson 2009). The central lemmatal awn length of the sessile or inconspicuously pedicelled spikelet was used by Beetle (1948) to differentiate between these two species but we found this measure to be completely overlapping. *Muhlenbergiabryophilus* is sister to a single entry of *M.uniseta* (Peterson 24712) in our new analysis (Fig. 1B). We have only a single marker (ITS) for *M.bryophilus*, so its alignment may change with additional plastid markers.

##### Specimens examined.

Peru. **Cusco**: Prov. Anta, Mollepata−Takawana, C. Vargas C. 19050 (USM); Prov. Urubamba, SW facing slope of Machu Picchu Mountain, V. Ugent 5324 (USM); **Junín**: Prov. Huancayo, Abajo de Pariahuanca, O. Tovar 7895, 7904 (USM); Cerro E of Huancayo, O. Tovar 2137 (USM); **La Libertad**: Prov. Bolivar, W of Longotea, 0.5 km towards San Vicente, 7°2'35.6"S, 77°52'39.8"W, 3202 m, 31 Mar 2008, P.M. Peterson 21966, R.J. Soreng & J. Montoya Quino (US, USM); **Lima**: Prov. Canta, 8 km SW of San Jose Canta towards Huamantango, 11°31'10"S, 76°42'16.3"W, 2770 m, 28 Mar 2004, P.M. Peterson & N. Refulio Rodríguez 17995 (US, USM); **Piura**: Prov. Huancabamba, La Beatita, S. Llatas Quiroz 1850, 1851 (CPUN); **Puno**: Prov. Sandia, Debajo de Cuyocuyo, R. Ferreyra & A. Vera Beuner 16636 (USM).

#### 3. Muhlenbergia caxamarcensis

Taxon classificationPlantaePoalesPoaceae

Lægaard & Sánchez Vega, Nordic J. Bot. 10:437. 1990.

Fig. 4A−C

##### Type.

Peru, Cajamarca, Micuypampa, 62 km from Cajamarca towards Celendín, 3600 m, 26 Mar 1988, *S.A. Renvoize & S. Lægaard 4962* (holotype: CPUN!; isotypes: AAU!, K!, MO-3712393!, US-3185350!).

##### Description.

Loosely caespitose *perennials*. *Culms* 8−12 cm tall, 0.2−0.4 mm diameter just below the panicle, erect to decumbent near base, slender, scaberulous to glabrous, profusely branched below; *lower internodes* 5−10 mm long with repeated intravaginal branching. *Leaf sheaths* 4−27 mm long, glabrous, generally longer than the internodes, rounded near base; *ligules* (1.5−) 1.8−2.5 mm long, hyaline, often lacerate, margins decurrent, apex obtuse; *blades* 0.5−1.5 cm long, 0.6−1.2 mm wide, flat or folded, prominently veined, sometimes conspicuously crystalline or spiculate on both surfaces, otherwise glabrous below, sparsely scaberulous above and along margins, tapering to a boat shaped tip. *Panicles* (1.0−)1.5−4.0 cm long, 1−3 mm wide, exserted or included in the uppermost sheath, loosely contracted, narrow; *primary branches* 0.5−2 cm long, appressed to the culm axis, one per node.*pedicels* 1−5 mm long, stiff, densely scabrous, spiculate, erect; *nodes* 4−6 per inflorescence. *Spikelets* 2.4−2.8 mm long (excluding the mucro or awn), plumbeous to reddish-purple; *glumes* 1.2−1.6 mm long, shorter than the floret, subequal to equal, 1-nerved, glabrous, often reddish-purple near apex and greenish-grey below, apex obtuse; *lemmas* 2.4−2.8 mm long, lanceolate, keeled, prominently 3-nerved, dark reddish-purple to plumbeous above greenish-grey below, sericeous, lower ½ to ¾ with scattered appressed hairs, the hairs 0.3−0.5 mm long, apex acuminate, mucronate or short-awned, the awn up to 1.5 mm long, scabrous; *paleas* 2.4−2.6 mm long, as long as the lemma, lanceolate, hairy between the nerves; *anthers* 1−1.3 mm long, purple or yellow. *Caryopses* 0.9−1.1 mm long, elliptic to fusiform, terete, yellowish-brown.

**Figure 4.**
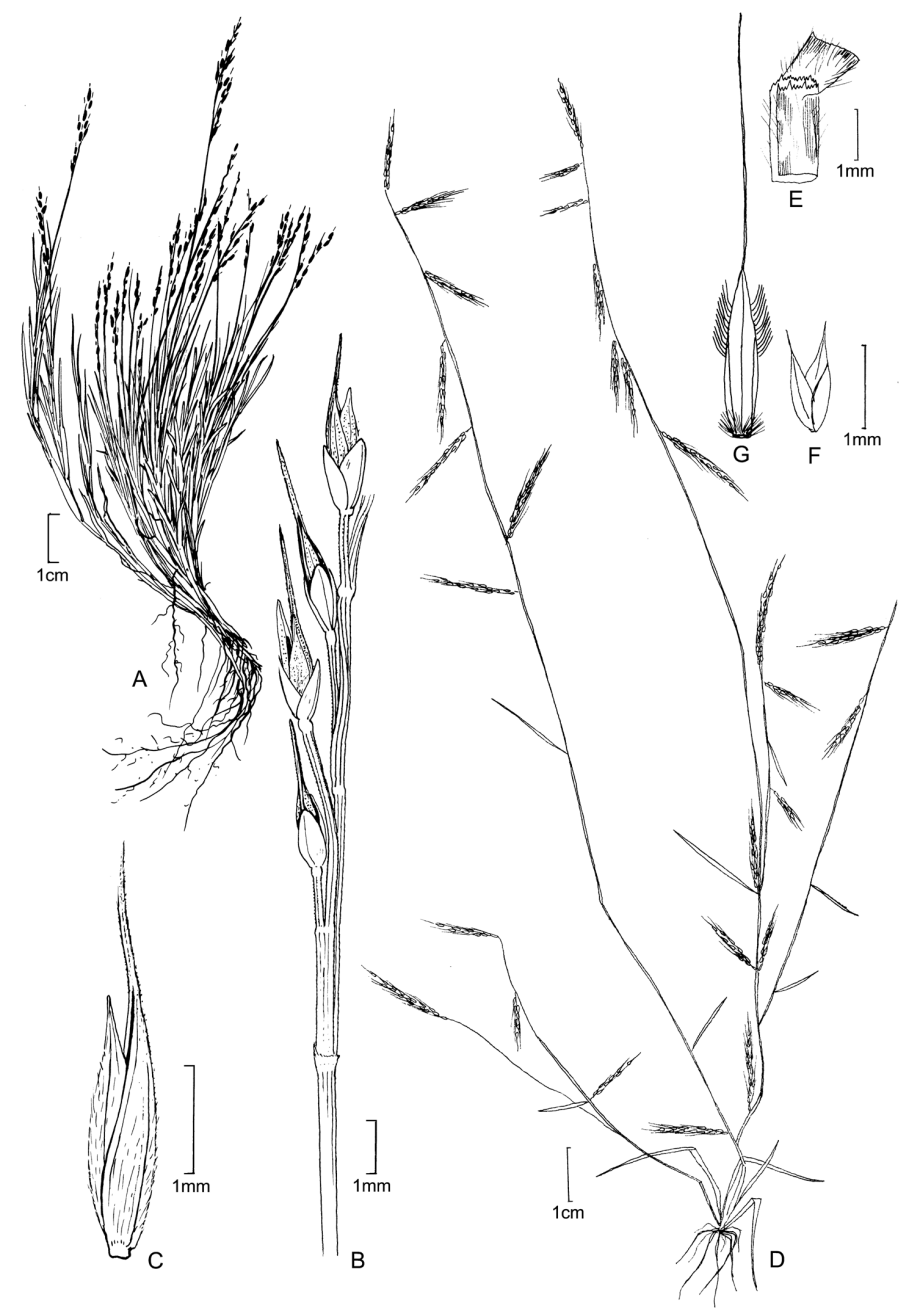
**A−C***Muhlenbergiacaxamarcensis* Lægaard & Sánchez Vega **A** habit **B** panicle branch **C** floret **D−G***Muhlenbergiaciliata* (Kunth) Trin **D** habit **E** ligule **F** glumes **G** floret. Drawings **A−C** from Lægaard and Sánchez Vega (1990) from holotype collection (*S.A. Renvoize & S. Lægaard 4962*) **D−G** from Peterson and Annable (1991) drawn from *P.M. Peterson & C.R. Annable 4541* (WS).

##### Distribution.

This species is endemic to Peru, known only from Cajamarca and La Libertad departments.

##### Ecology.

*Muhlenbergiacaxamarcensis* occurs on shallow soils on rock shelves and rocky outcrops often of calcareous origins, mud flats, open grassy meadows and slopes associated with *Festuca, Carex, Calamagrostis* (probably better treated as *Cinnagrostis* Griseb. but combinations are not yet made; Saarela et al. 2017, Soreng et al. 2017), *Polylepis*, *Muhlenbergialigularis*, *M.peruviana* and *M.cenchroides*; 3000−3600 m. Flowering March through May.

##### Comments.

*Muhlenbergiacaxamarcensis* is morphologically similar to *M.ligularis* and *M.fastigiata* and can be separated from both of these species in possessing sericeous florets (lemma and palea) with hairs readily visible under 10× magnification.

*Muhlenbergiacaxamarcensis* is a member of M.subg.Bealia and is sister to the North American *M.filiformis* (Thurb. ex S. Watson) Rydb.– *M.vaginata* Swallen pair (Peterson et al. 2010b). In our new analysis, *M.caxamarcensis* appears to have evolved within *M.ligularis* (Fig. 1A, plastid only). However, the ITS marker clearly aligns *M.caxamarcensis* with a single accession of *M.ligularis* (Fig. 1A). This pair is sister to *M.filiformis* and all three accessions are sister to *M.vaginata*. These results suggest multiple origins for *M.caxamarcensis* from North and South American progenitors.

##### Specimens examined.

PERU. **Cajamarca**: Prov. Cajamarca, 16 km W of Cajamarca up road (Avenida Peru) towards Cumbemayo, 3440 m, 31 Mar 1997, P.M. Peterson & N. Refulio Rodriguez 14010 (US, USM); 18 km W of Cajamarca up road (Avenida Peru) towards Cumbe Mayo, 3600 m, 31 Mar 1997, P.M. Peterson & N. Refulio Rodriguez 14013 (US, USM); 29 km from Cajamarca on road to Celendín, S.A. Renvoize & S. Lægaard 4974 (AAU, CPUN, K, US); Cumbe Mayo, I. Sánchez Vega & W. Ruiz Vigo 598 (CPUN), I. Sánchez Vega 4690 (CPUN); Cumbe Mayo, W of Cajamarca, S.A. Renvoize 5000, 5004 & 5005 S. Lægaard & I. Sánchez Vega (AAU, CPUN, K, US); Micuypampa, 62 km from Cajamarca towards Celendín, S.A. Renvoize & S. Lægaard 4961 (AAU, CPUN, K, US); Prov. San Miguel, 61 km N of Cajamarca on hwy 3N towards Bambamarca, 3640 m, 16 Mar 2000, P.M. Peterson & N. Refulio Rodriguez 14916 (CPUN, US, USM); Cajamarca to Celendín, 3 km NE of Encañada, I. Sánchez Vega 2777, V. Torrel & E. Medina (CPUN); S.A. Renvoize & S. Lægaard 4849 (CPUN). **La Libertad**: Prov. Bolivar, 3 air km ESE of Longotea on road to Bolivar, 3202 m, 31 Mar 2008, P.M. Peterson 21965, R.J. Soreng & J. Montoyo Quino (US, USM); Nevado Cajamarquilla, 3500 m, 9 Sep 1946, J. Infantes Vera 937 (MOL).

#### 4. Muhlenbergia cenchroides

Taxon classificationPlantaePoalesPoaceae

(Humb. & Bonpl. ex Willd.) P.M. Peterson, Caldasia 31(2): 280, f. 2 C–D. 2009.

Fig. 3C, D

Aegopogon cenchroides Humb. & Bonpl. ex Willd., Sp. Pl. 4(2):899. 1806. Type: Venezuela, Sucre, Cumaná, *F.W.H.A. Humboldt & A.J.A. Bonpland s.n.* (holotype: B-W-01637-020 [image!]; isotypes: P!, US-75957 fragm. ex P!).
Aegopogon geminiflorus Kunth, Nov. Gen. Sp. (quarto ed.) 1:133, t. 43. 1815 (1816). Aegopogoncenchroidesvar.geminiflorus (Kunth) Griseb., Abh. Königl. Ges. Wiss. Göttingen 24:301. 1879. Type: Venezuela, Amazonas, inter Cerro Duida et Rio Tamatama, juxta Esmeralda, May, F.W.H.A. *Humboldt & A.J.A. Bonpland s.n.* (holotype: P; isotype: US-75956 fragm. ex P!).
Hymenothecium quinquesetum Lag., Gen. Sp. Pl. 4. 1816. Aegopogonquinquesetus (Lag.) Roem. & Schult., Syst. Veg. 1:805. 1817. Type: Mexico, México Iperio, *Ludovicus Nee* (holotype: MA; isotype: BAA-00002156 [image!]).
Hymenothecium trisetum Lag., Gen. Sp. Pl. 4. 1816. Aegopogontrisetus (Lag.) Roem. & Schult., Syst. Veg. 2:805. 1817. Aegopogoncenchroidesvar.trisetus (Lag.) E. Fourn., Mexic. Pl. 2:72. 1886. Type: Mexico, México Imperio (holotype: MA; isotype: BAA-00002158 [image!]).
Aegopogon setifer Nees, Linnaea 19(6):691. 1847. Type: Mexico, *A. Aschenborn 132* (holotype: B; isotypes: FR-0036375 [image!], FR-0036376 [image!], US-75953 fragm. ex B!).
Aegopogon cenchroides var. multisetus E. Fourn., Mexic. Pl. 2: 72. 1886. Type: Mexico, Moran. in rupibus, 1840, *H. Galeotti 5808* (lectotoype: BR, designated by P.M. Peterson, Contr. U.S. Natl. Herb. 41: 10. 2001; isolectotypes: P!, US-75958 fragm. ex P!).

##### Description.

Caespitose *perennials* often sprawling, occasionally with stolons. *Culms* (10–) 25–55 cm long, glabrous below the nodes; *internodes* glabrous. Leaf sheaths mostly 0.8–8 cm long, shorter than the internodes, glabrous; *ligules* 1–2 mm long, apex acute, lacerate; *blades* 1.5–6 cm long, 0.5–2 mm wide, flat, scaberulous above, smooth beneath. *Panicles* 2–8 cm long, 0.5–1.2 cm wide, open, loosely-flowered with recemosely arranged branches; *primary branches* 2–4 mm long, excluding the awns, one per node, often purplish. *Spikelet* fasciles of three with one sessile or subsessile perfect spikelet and two short-pedicelled lateral spikelets staminate or sterile, the *pedicels* less than 0.2–0.5 mm long and the other two spikelets short-pedicelled, the *pedicels* about 0.7–1.2 mm long; *glumes* (1−)1.5–2.8 mm long, oblong and wider distally, 1-veined, apex deeply notched, awned, the awns 2–4 mm long, lobes triangular, acute; *lemmas* 2.5–3 mm long, fusiform, 3-awned, the central awns 5–13 mm long, lateral awns 2–3 mm long; *paleas* 2.5–3 mm long, puberulent, apex awned, the awns 1–2 mm long; *anthers* 1.6–1.8 mm long, yellowish to purplish. *Caryopses* 1−1.4 mm long, fusiform. 2*n* = 40, 60, 80.

**Figure 5.**
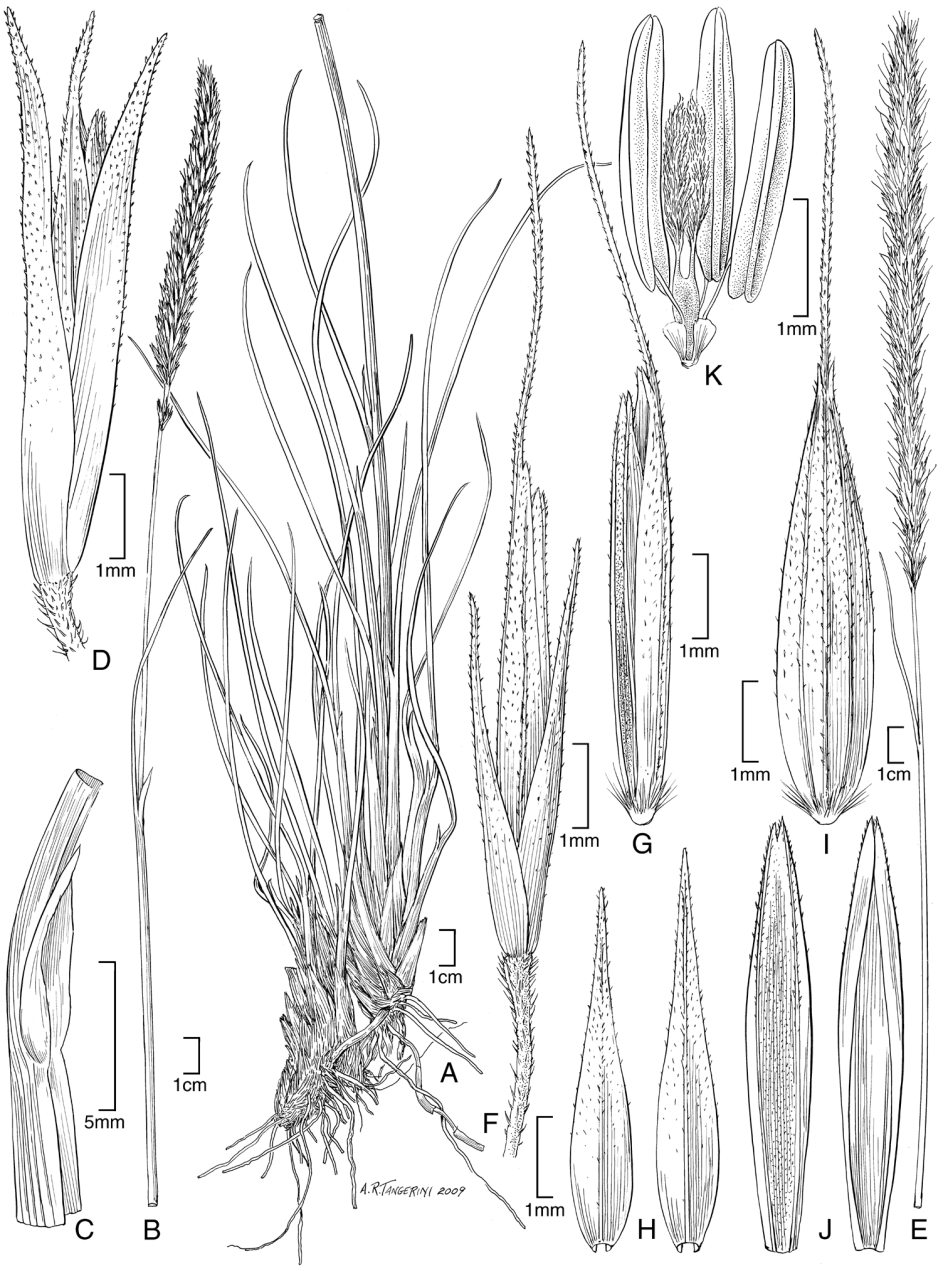
**A−D***Muhlenbergiacoerulea* (Griseb.) Mez **A** habit **B** culm and panicle **C** ligule **D** spikelet **E−K***Muhlenbergiacoerulea* (Griseb.) Mez × *Muhlenbergiarigida* (Kunth) Kunth **E** culm and panicle **F** spikelet **G** floret **H** glumes **I** lemma **J** paleas, dorsal and ventral view **K** lodicules, stamens, and pistil. **A−D** drawn from *P.M. Peterson, R.J. Soreng & J. Montoya Quino 21957* (US) **E−K** drawn *from P.M. Peterson & N. Refulio Rodriguez 14860* (US).

##### Distribution.

*Muhlenbergiacenchroides* ranges from throughout México, Central America to South America in Bolivia, Brazil, Colombia, Ecuador, Peru, Guyana and Venezuela.

##### Ecology.

*Muhlenbergiacenchroides* occurs on rocky slopes, canyons, cliffs, roadcuts, arroyos, seeps and meadows associated *Baccharis* spp., *Festucamyuros*, *Alnus*, *Polylepis*, *Fucaria*, *Salvia* spp., *Urochloa, Salvia* spp., *Calceolaria* spp., *Eupatorium*, *Festuca*, *Puya*, *Rubus*, *Schizachyrium*, *Cenchrusclandestinus*, *Calamagrostis* spp., *Muhlenbergia* spp., *Hyptis*, *Oxalis*, *Aristida, Begonia*, *Adiantum, Bidens, Lepechinia, Oreocallisgrandiflora* (Lam.) R. Br., *Desmodium*, *Cortaderiabifida* Pilg., *Cortaderiajubata* (Lemoine ex Carriere) Stapf, *Sporobolus*, *Monnina*, *Carex*, *Cheilanthes*, *Eragrostis*, *Lupinus*, *Lycopodium*, *Jarava*, *Nassella*, *Werneria*, *Agave*, *Eucalyptus*, *Thalictrum* and *Chusquea*; 1430–3850 m. Flowering December through September.

##### Comments.

*Muhlenbergiacenchroides* can be separated from *M.bryophilus* in having anthers 1.6−1.8 mm long, the perennial habit with (10–) 25–55 cm tall culms and sessile or inconspicuously pedicelled perfect spikelets usually associated with two staminate or sterile pedicelled spikelets (Tovar 1993; Giraldo-Cañas and Peterson 2009).

*Muhlenbergiacenchroides* lies in a clade with *M.tarahumara, M.uniseta* and *M.bryophilus* in M.subg.Muhlenbergia (Fig. 1B; Peterson et al. 2010b). The multiple sampled individuals of *M.cenchroides* and *M.uniseta* in the current analyses do not resolve into reciprocally monophyletic lineages, but one sublineage, including accessions of each species, is strongly supported. This strongly suggests gene flow among individuals of these two species.

##### Specimens examined.

Peru. **Ancash**: Prov. Bolognesi, Paramarca, cerros al E de Chiquián, 3680 m, 8 Apr 1952, E. Cerrate 1396 (MOL, USM); Prov. Carhuaz, Cordillera Negra, 22 km SW of Shupluy and Rio Santa, 9°17'19.7"S, 77°44'39.3"W, 3770 m, 13 Mar 2008, P.M. Peterson 21652, R.J. Soreng, M.I. La Torre & J.V. Rojas Fox (US, USM); Prov. Corongo, Cordillera Blanca, 3 km E of Yanac, 8°35'55.6"S, 77°50'4.8"W, 2849 m, 18 Mar 2008, P.M. Peterson & R.J. Soreng 21786 (US, USM); Prov. Corongo, Cordillera Blanca, E of Yungay, 3400 m, 5 Apr 1988, S.A. Renvoize & S. Lægaard 5055 (CPUN, K); Prov. Huaraz, W side of Cordillera Blanca, S of Quebrada Ishinca, 9°24'55.8"S, 77°32'45.8"W, 2885 m, 12 Mar 2008, P.M. Peterson 21614, R.J. Soreng, M.I. La Torre & J.V. Rojas Fox (US, USM); Prov. Huaraz−Recuay border, W side of Rio Santa Canyon, 19 km S of Huaraz, 9°40'58.1"S, 77°28'29.7"W, 3202 m, 11 Mar 2008, P.M. Peterson 21600, R.J. Soreng, M.I. La Torre & J.V. Rojas Fox (US, USM); Prov Huaylas, Cordillera Blanca, encima de Caraz, 3200 m, A Weberbauer 3234 (MOL); Prov. Huaylas, Huata, M.I. La Torre 2793 (USM); Prov. Pallasca, 17 km N of Huandoval and 6 km S of Pallasca, 3360 m, 27 Mar 1997, P.M. Peterson & N. Refulio Rodríguez 13927 (US, USM); 3.4 km N of Huandoval towards Huascaschuque, 8°18'59.5"S, 77°58'9.5"W, 2949 m, 21 Mar 2008, P.M. Peterson & R.J. Soreng 21817 (US, USM); Prov. Huari, near Laguna de Puruhay, M.I. La Torre 3692 (USM); San Marcos, A. Cano 13505 (USM); Huascarán National Park, Quebrada Rurichinchay, D. Smith 12659, A. Gonzales & D. Maldonado (USM); Prov. Recuay, Cordillera Blanca, 16 km E of Catac on road towards Chavin de Huantar, 3630 m, 21 Mar 1997, P.M. Peterson & N. Refulio Rodríguez 13833 (US, USM); Prov. Yungay, Cordillera Blanca, 20 km ENE of Yungay, 9°6'26.2"S, 77°41'8.2"W 3247 m, 15 Mar 2008, P.M. Peterson 21712, R.J. Soreng, M.I. La Torre & J.V. Rojas Fox (US, USM); Quebrada Llauganuco, 24 km ENE of Yungay, 9°5'46.3"S, 77°40'19.3"W, 3309 m, 16 Mar 2008, P.M. Peterson 21739, R.J. Soreng, M.I. La Torre & J.V. Rojas Fox (US, USM); Prov. Yungay, Huascarán National Park, Llanganuco sector, María Josefa trail between Chinancocha and Pucayacu, 77°39'W, 9°05'S, 3700−3850 m, 7 May 1985, D.N. Smith 10512 (CPUN, MO). **Ayacucho**: Ayna, L. Aucasime 258 (USM); Seqseqa, L. Aucasime 81 (USM); Prov. Huanca Sancos, 27 km NW of Putajasa and 3 km S of Sacsamarca, 13°57'51.1"S, 74°18'41.5"W, 3650 m, 25 Feb 2002, P.M. Peterson 16278, A. Cano, M. I. La Torre, A. Ramirez & D. Susanibar Cruz (US, USM). **Cajamarca**: near Cajamarca, 2700 m, 11 Mar 1986, B, Becker & F.M. Terrones H. 601 (LPB); Cajamarca to Celendín, 95 km from Cajamarca, above Quillimbas, 3000 m, 22 Mar 1988, S.A. Renvoize & S. Lægaard 4868 (CPUN, K); Prov. Cajamarca, 16 km W of Central Plaza of Cajamarca up road towards Cumbemayo on Avenida Peru, 3440 m, 31 Mar 1997, P.M. Peterson & N. Refulio Rodríguez 14012 (US, USM); Prov. Cajamarca, Dist. Baños del Inca, Tres Tingos, margen Oeste del río Chonta, 2900 m, 2 Mar 2002, I. Sánchez Vega 11261 (CPUN); Prov. Cajamarca, Cerro Huairapongo–Baños del Inca, 19 Nov 1965, I. Sánchez Vega 126 & Julio Mercado (CPUN); Prov. Cajamarca, Fundo Aylambo, a 5 km antes de la ciudad, 8 May 1969, I. Sánchez Vega 385 & W. Ruiz Vigo (CPUN); Prov. Cajamarca, cerca al Paso El Gavilán, 3100 m, 19 Apr 1975, I. Sánchez Vega 1407, P. Brandelard & J. Sanabria (CPUN); Prov. Cajamarca, Laguna de Moyococha, 2550 m, 29 Aug 1965, I. Sánchez Vega 92 (CPUN); Prov. Cajamarca, Dist. San Juan, lugar Huacraruco, 2350 m, 19 Jun 1993, J. Cabanillas S. & J. Guevara B. 615 (CPUN); Chotén, en el Arboretum de CICAFOR, a 4 km de la carretera Pacasmayo–Cajamarca, 2990 m, 23 May 1981, I. Sánchez Vega 2562, V. Torrel & E. Medina (CPUN); Prov. Cajamarca, Dist. Cajamarca. Paso El Gavilán, 3100 m, 26 Jun 1966, I. Sánchez Vega 239 (CPUN); Prov. Cajamarca, ladera que converge al Valle Cajamarca, Lla Colpa, 2900 m, 22 Apr 1975, I. Sánchez Vega 1416, P. Brandelard, J, Sanabria & W. Ruiz Vigo (CPUN); Prov. Cajamarca, Sais Atahualpa de Porcón, en el Arboretum de CICAFOR, 3400 m, 16 May 1981, I. Sánchez Vega 2512, V. Torrel P. & E. Medina (CPUN); Prov. Cajamarca, Dist. Namora, Hacienda Polloquito, 3000 m, 6 May 1967, I. Sánchez Vega 321 (CPUN);12 km N of Cajamarca on road towards Bambamarca, 3020 m, 14 Mar 2000, P.M. Peterson & N. Refulio Rodríguez 14855 (CPUN, US, USM); S of Paso El Gavelan, 10 air km S of Cajamarca, 7°15'37.2"S, 78°30'34.5"W, 2544 m, 26 Mar 2008, P.M. Peterson 21875, R.J. Soreng & I. Sánchez Vega (US, USM); cumbre El Gavilán, R. Ferreyra 3255 (USM); Prov. Celendin, 4 air km W of Celendin, 6°54'13.5"S, 78°10'8.4"W, 2687 m, 29 Mar 2008, P.M. Peterson 21925, R. J. Soreng & J. Montoya Quino (US, USM); Prov. San Ignacio, 31 km E of Sondor on road towards Tabaconas, 2490 m, 31 Mar 2000, P.M. Peterson & N. Refulio Rodríguez 15128 (CPUN, US, USM); Prov. San Miguel, Niepos, 2200 m, 30 Oct 1985, S. Llatas 1503 (CPUN); Prov. San Pablo, Dist. San Pablo, lugar El Molino, 2200 m, 12 Jun 1993, J. Sánchez Vega 663 (CPUN). **Cusco**: Prov. Calca, Manto a Amparaes, 2650 m, 18 Apr 1966, C. Vargas C. 17393 (LPB); Prov. Cusco, Socorro−K’inka, C. Vargas C. 13161 (USM); Ruinas de Sacsahuamán, 3500 m, 6 May 1983, S.G. Beck 8365 (LPB); Mandor, abajo de Machupicchu, 2000 m, 13 Apr 1957, H. Ellenberg 988 (LPB); Cusco−Huancaro hwy, P. Gutte & G. Muller 9425a (USM); Machupicchu, camino entre la ciudad inca de Machupicchu y el Puente del Inca, 2000 m, 18 Sep 2004, D. Giraldo-Cañas 3759 (COL). **Huancavelica**: Prov. Huancavelica, Huando, O. Tovar 1254 (USM). **Huánuco**: Prov. Huánuco, Carpish, cumbre entre Huánuco y Tingo María, 2700 m, 1 Oct 1950, R. Ferreyra 8070 (MOL); Prov. Huánuco, Carpish, cumbre entre Huánuco y Tingo María, R. Ferreyra 10022 (USM); 48.6 km NE of Huánuco towards Tingo María, T. Plowman & P.M. Rury s.n. (USM); Prov. Pachitea, 21 air km ENE of Huánuco, 9°51'53.1"S, 76°2'55.1"W, 2498 m, 6 Mar 2007, P.M. Peterson 20341, R.J. Soreng & K. Romaschenko (US, USM); Canyon of the Rio Grande, 44 air km E of Huánuco, P.M. Peterson 20363, R.J. Soreng & K. Romaschenko (US, USM). **Junín**: Prov. Huancayo, near Paián, O. Tovar 9311 (USM); Cerro al E de Huancayo, 3900 m, 2 May 1954, O. Tovar 2173 (US, USM); Prov. Tarma, entre Huancapistana, 2200 m, A. Weberbauer 2042 (MOL). **La Libertad**: Prov. Bolivar, entre el desvío a Uchumarca y Santa Luisa, 7°04'S, 77°49'W, 3700 m, 11 Nov 2001, I. Sánchez Vega 11194, M. Dillon & G. Iberico (CPUN); Prov. Bolivar, E side of Cerro Salumpuy, 8 air km NW of Bolivar, 7°7'39.2"S, 77°46'9.9"W, 3390 m, 30 Mar 2008, P.M. Peterson 21942, R. J. Soreng & J. Montoya Quino (US, USM); 3 air km ESE of Longotea on road to Bolivar, 7°3'27.9"S, 77°51'2.2"W, 3202 m, 31 Mar 2008, P.M. Peterson 21961, R. J. Soreng & J. Montoya Quino (US, USM). **Lambayeque**: Prov. Ferreñafe, Inkawasi, S. Llatas Quiroz 4122 (USM). **Pasco**: Prov. Oxapampa, Río Boqueria, 26 km from Oxapampa via Río Yamaquizu, D. Smith 1807, A. Pretel & L. Acosta (USM). **Piura**: Prov. Huancabamba, Porculla, 2200 m, 10 May 1992, S. Llatas Quiroz & H. De la Cruz S. 3098 (CPUN). **San Martín**: Prov. Mariscal Cáceres, puerto de Norte de Río Abiseo, A. Cano 7466, B. Young & J. Roque (USM).

#### 5. Muhlenbergia ciliata

Taxon classificationPlantaePoalesPoaceae

(Kunth) Trin., Gram. Unifl. Sesquifl. 193. t.5, f.16. 1824.

Fig. 4D−G

Podosemum ciliatum Kunth, Nov. Gen. Sp. (quarto ed.) 1:128−129. 1816. Trichochloaciliata (Kunth) Roem. & Schult., Syst. Veg. 2:386. 1817. Polypogonciliatus (Kunth) Spreng., Syst. Veg. 1:243. 1825. Type: Mexico, Michoacán, Volcán de Jorullo, Sep, *F.W.H.A. Humboldt & A.J.A. Bonpland s.n.* (holotype: P-HBK!; isotypes: BAA-1619 ex P!, BM!, P-Bonpl!, US-91918 fragm. ex P!).
Muhlenbergia adspersa Trin., Mém. Acad. Imp. Sci. Saint-Pétersbourg, Sér. 6, Sci. Math., Seconde Pt. Sci. Nat. 4(3–4):291. 1841. Type: Peru, Lima, *ex herb. C.H. Mertens s.n.* (holotype: LE?; isotypes: LE-TRIN-1486.01 fragm. ex LE herb. Mertens!, US-87236 fragm. ex LE herb. Mertens!).

##### Description.

Sprawling, slender *annuals*. *Culms* 8–30 (−50) cm tall, glabrous, filiform, often tufted, freely branching at lower nodes; 0.2–0.5 mm diameter just below the inflorescence; *internodes* 6–42 mm long. *Leaf sheaths* (8−) 20–44 mm long, glabrous or sparsely pilose along the margins, shorter than the internodes; *ligules* 0.2–0.8 mm long, a ciliate membrane; apex truncate; margin with a tuft of hairs up to 1 mm long; *blades* 1–4 cm long, 0.6–1.4 mm wide, flat or loosely involute, often sparsely pilose above, glabrous below. *Panicles* 4–12 cm long, 1.8–5.0 cm wide, terminal, densely flowered; *primary branches* 1.5–3.7 cm long spreading and reflexed at maturity up to 90° from the rachises, one per node; *pedicels* 0.5–3 mm long, glabrous, appressed, erect; nodes 6–13 per panicle; *Spikelets* appressed to the branches, overlapping; *glumes* 0.7–1.7 mm long, subequal, glabrous, 1-nerved; apex acuminate, often mucronate; the mucro up to 0.5 mm long; *lower glumes* 0.7–1.5 mm long; *upper glumes* 0.8–1.7 mm long; *lemmas* 1.8–2.5 mm long, lanceolate, slender, awned, strongly 3-nerved but appearing five-nerved, the intermediate “nerves” actually rows of short barbs on top of folded epidermal ridges, sometimes with prominent short hairs (scabers) along the lateral nerves, often appearing glabrous without magnification, awns 5–11(−18) mm long, flexuous; *callus* minutely short pubescent; *paleas* 1.6–2.4 mm long, narrowly lanceolate, glabrous; *anthers* 0.3–0.5 mm long, yellowish. *Caryopses* 0.8–1.8 mm long, narrowly fusiform, brownish. 2*n* = 20.

##### Distribution.

*Muhlenbergiaciliata* is found throughout México and Central America in Ecuador, Peru, Brazil, Bolivia, Brazil and Argentina.

##### Ecology.

*Muhlenbergiaciliata* is found on moist to dry soils usually beneath taller vegetation, sandy drainages, steep rocky slopes, rock outcrops and disturbed roadsides in woodlands with *Acacia*, *Agave*, *Andropogon*, *Bidens*, *Baccharis* spp., *Bothriochloa*, *Eupatorium*, *Melinusminutiflora* P. Beauv., *Muhlenbergiabryophilus*, *M.flexuosa*, *M.rigida*, *Puya* and *Salvia*; 1000–2400 m.

##### Comments.

*Muhlenbergiaciliata* is morphologically similar to *M.romaschenkoi* and can be differentiated from the latter in having short ligules 0.2−0.8 mm long [1.2−3.0 (−5.0) mm long in *M.romaschenkoi*], paleas that are glabrous between the veins on the proximal ½ (versus appressed pubescent), 6−13 nodes along the panicle (versus 15−23) and *panicles* that are 4−12 cm long (versus 7−15 cm long) [Peterson and Giraldo-Cañas 2011, 2012]. Some individuals of *M.ciliata* from Peru do not have prominent cilia along the lateral veins that are present in most individuals from North America.

*Muhlenbergiaciliata* is closely related to *M.pectinata* C.O. Goodd. (North America) and *M.tenella* (Kunth) Trin. (North America, Central America and Colombia) [Peterson and Annable 1991]. These three species form a clade in Muhlenbergiasubg.Muhlenbergia (Peterson et al. 2010b; Fig. 1B).

##### Specimens examined.

Peru. **Amazonas**: Piedra Grande, near Rio Santo Domingo, 5000 ft alt., 14−19 May 1923, J.F. Macbride 3684 (F, US). **Cusco**: Urubamba, Ruinas de Macchu Picchu, 2500 m, 27 May 1963, D. Ugent 5356 (US), 2300 m, 11 Apr 1963, C. Vargas C. 14354 (US), Mar 1987, I. Grignon 2306 (AAU, US); Quebrada Termas Macchu Picchu, 25 Mar 1966, C. Vargas C. 17145 (USM). **Huánuco**: 21 air km NE of Pachitea, 7 km E of Puerto Rancho junction, 9°49'45.9"S, 76°3'12.9"W, 2094 m, 6 Mar 2007, P.M. Peterson, R.J. Soreng & K. Romaschenko 20336 (US); Pachitea, canyon of the Rio Grande, along trail 1 air km NW of Estación Huacachay, 9°50'44.7"S, 75°50'5.2"W, 2323 m, 8 Mar 2007, P.M. Peterson, R.J. Soreng & K. Romaschenko 20378 (US); 2 air km ESE of Eastación Huacachay, 9°51'34.7"S, 75°50'48.1"W, 1867 m, 7 Mar 2007, P.M. Peterson, R.J. Soreng & K. Romaschenko 20359 (US). **Junín**: between Tarma and La Merced, 3000 m, 14 Oct 1923, A.S. Hitchcock 22154 (US); Mito, 9000 ft alt., 8−18 Apr 1923, J.F. Macbride 3376 (F, US); Province Huancayo, abajo de Pariabuena, 2000 m, 6 May 1969, O. Tovar 7936 (USM). **La Libertad**: Bolivar, W of Longotea ca. 0.5 km towards San Vicente on Balsas−Longotea road, 7°2'35.6"S, 77°52'39.8"W, 2487 m, 31 Mar 2008, P.M. Peterson, R.J. Soreng & J. Montoya Quino 21967 (US).

#### 6. Muhlenbergia coerulea

Taxon classificationPlantaePoalesPoaceae

(Griseb.) Mez, Repert. Spec. Nov. Regni Veg. 17:213. 1921.

Figs 5A−D , 6A−C

Epicampes coerulea Griseb., Abh. Königl. Ges. Wiss. Göttingen 19:256. 1874. Type: Sierra de Tucumán, Den grossen isolierten Buescheln an der Cuesta de Juntas, 23 Mar 1872, *P.G. Lorentz 86* (lectotype, designated here: GOET-006633 [image!]; isolectotypes: CORD-00004624!, CORD-00004625!, US-865981!).
Crypsis phleoides Kunth, Nov. Gen. Sp. (quarto ed.) 1:140. 1816, non Muhlenbergiaphleoides (Kunth) Columbus. Cinnaphleoides (Kunth) Kunth, Révis. Gramin. 1:67. 1829. Epicampesphleoides (Kunth) Griseb., Abh. Königl. Ges. Wiss. Göttingen 19: 256. 1874. Type: Venezuela, Sucre, Sep, *F.W.H.A. von Humboldt & A.J.A. Bonpland s.n.* (holotype: P!; isotype: US-A865652 fragm. ex P!).
Crypsis stricta Kunth, Nov. Gen. Sp. (quarto ed.) 1:140. 1816, non Muhlenbergiastricta (J. Presl) Kunth. Cinnastricta (Kunth) Kunth, Révis. Gramin. 1:67. 1829. Crypsinnastricta (Kunth) E. Fourn., Mexic. Pl. 2:90. 1886. Type: Peru, Prov. de los Pastos, Dec, *F.W.H.A. von Humboldt & A.J.A. Bonpland s.n.* (holotype: B-W; isotype: US-A865653 fragm. ex B-W!).
Epicampes coerulea var. submutica Hack., Anales Mus. Nac. Buenos Aires 13:471. 1906. Type: Argentina, Tucumán, Dep. Tafí, Cuesta de Malamala, 2500 m, 4 Feb 1904, *Stuckert herb. Arg. 14900 ex Lillo 3402* (holotype: W-1916-0026677 [image!]; isotypes: CORD-00001624 [image!], LIL-000053 [image!], US3168604!).

##### Description.

Densely caespitose *perennials*. *Culms* 50−100(−120) cm tall, erect, rigid, hirsute below the basal, terete nodes; usually 1 node per culm; *internodes* mostly glabrous and scabrous. *Leaf sheaths* mostly 5−18 cm long, glabrous to scaberulous above, rounded near base, lower sheaths often becoming fibrous with age; *ligules* 8−15 mm long, firm below, strongly decurrent, often lacerate, apex obtuse to acute; *blades* 15−35(−45) cm long, 2−5 mm wide, tightly involute, glabrous to scaberulous below mostly scaberulous near above. *Panicles* (6−)10−24(−30) cm long, 6−15 mm wide, narrow, spike-like, usually plumbeous; *primary branches* 0.2−2.5 cm long, erect and tightly ascending appressed, floriferous to base; *pedicels* 0.5−4 mm long, usually shorter than the spikelets, hispid. *Spikelets* 5−7(−8) mm long, plumbeous, rarely 2-flowered; *glumes* (4−)5−7(−8) mm long, usually as long or longer than the floret, sometimes a little shorter, lanceolate, about equal, 1-nerved, scabrous, apex acuminate, unawned, mucronate, or short-awned; *lemmas* (4.6−)5−6.7(−7.1) mm long, lanceolate, plumbeous, scabrous, apex acuminate, unawned, mucronate or short-awned, the awn 1−3(−4) mm long, inserted just below the apex; *callus* sparsely short bearded, the hairs 0.1−0.2 mm long; *paleas* (4.6−)5−6.5 mm long, about as long as the lemma, narrowly lanceolate, scaberulous, apex acuminate; *anthers* 2−3 mm long, purplish or yellowish. *Caryopses* 2.5−3.5 mm long, fusiform, dark reddish-brown.

**Figure 6.**
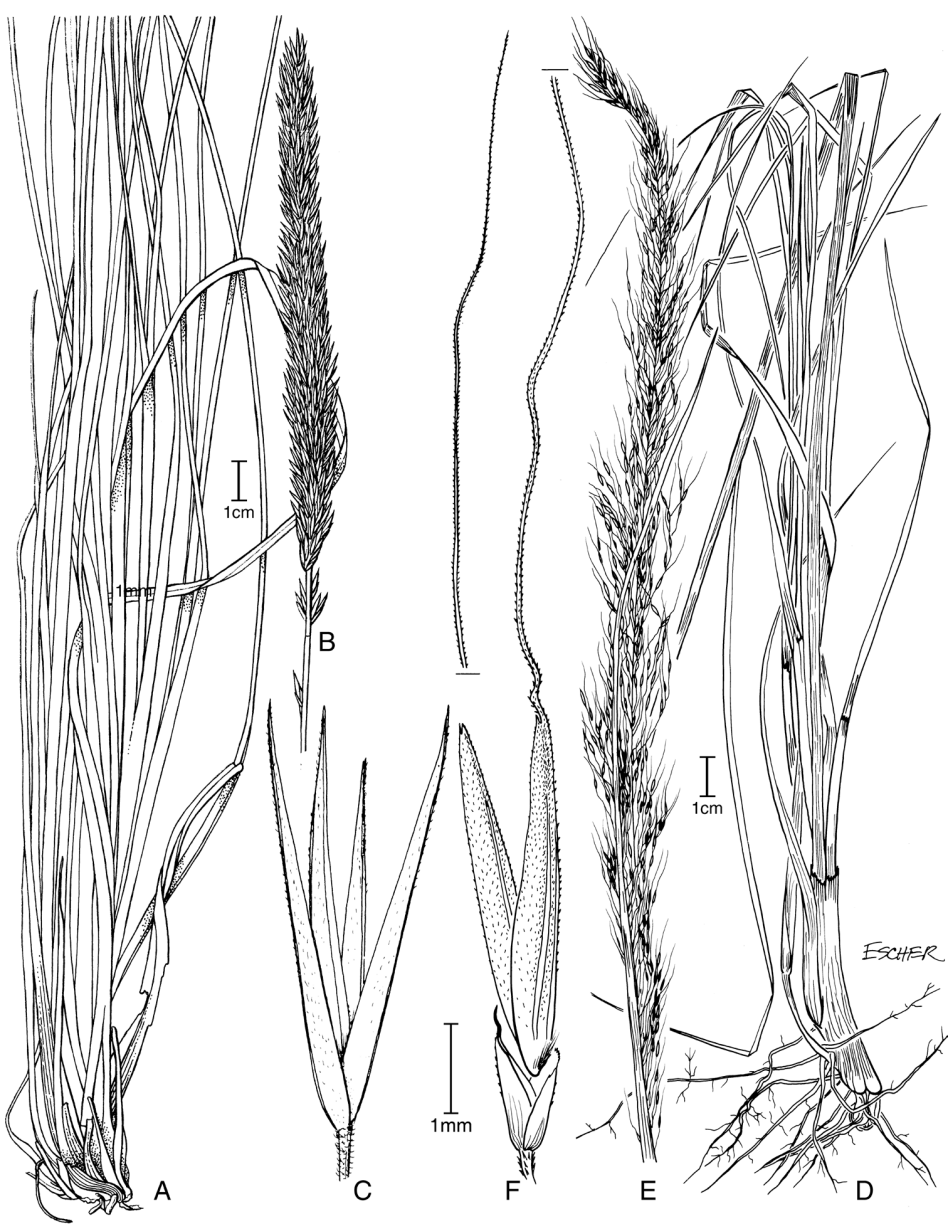
**A−C***Muhlenbergiacoerulea* (Griseb.) Mez **A** habit **B** panicle **C** spikelet **D−F***Muhlenbergiarigida* (Kunth) Kunth **D** habit **E** panicle **F** spikelet. Drawings from Giraldo-Cañas and Peterson (2009), Peterson and Giraldo-Cañas (2011). **A−C** drawn from *S.G. Beck 7700* (LPB) **D** drawn from *P.M. Peterson 9659* (US) **E, F** drawn from *P.M. Peterson, C.R. Annable & J. Valdés-Reyna 10876* (US).

##### Distribution.

*Muhlenbergiacoerulea* ranges throughout the Andean Cordillera from Colombia, Venezuela, Ecuador to Argentina, Peru and Bolivia.

##### Ecology.

*Muhlenbergiacoerulea* grows on rocky, often sandy slopes, derived from volcanic origins usually near arroyos associated with of *Alnus*, *Anatherostipaobtusa* (Nees & Meyen) Peñail., *Aristida*, *Calamagrostis*, *Calceolaria*, *Festuca*, *Hypericum*, *Jaravaichu* Ruiz & Pav., *Lupinus*, *Monnina*, *Nassella*, *Oreocallisgrandiflora* (Lam.) R. Br., *Polylepis*, *Puya*, *Rubus*, *Schizachyrium* and cultivated *Pinus*; 2450−4900 m. Flowering February through August.

##### Comments.

In recent literature (Giraldo-Cañas and Peterson 2009; Peterson and Giraldo-Cañas 2011, 2012), this species was referred to as *Muhlenbergiaangustata* (J. Presl) Kunth but the type [*Podosemumangustatum* J. Presl] clearly aligns with what we had been referring to as the hybrid between this species and *M.rigida*. Therefore, we adopt the next available name, *Muhlenbergiacoerulea* and change the hybrid designation to *M.coerulea* × *M.rigida*. Many specimens studied are intermediate between *M.coerulea* and *M.rigida*. These are treated as hybrids [*M.coerulea* (Griseb.) Mez × *M.rigida* (Kunth) Kunth; Lægaard and Peterson 2001; Peterson and Giraldo-Cañas 2011, 2012] since the size, width and colour of the *panicles* and the surface structure and length of the glumes and lemmas, are intermediate between these two recognised species.

*Muhlenbergiacoerulea* is deeply embedded in the M.subg.Trichochloa clade, a group of tall, rather robust perennials historically placed in *Podosemum* or *Epicampes* (Soderstrom 1967; Peterson et al. 2010b; Fig. 1A).

##### Specimens examined.

Peru. **Ancash**: Prov. Huaylas, Pueblo Libre, Antircán, A. Cano 11714 (USM); Prov. Huari, Huascarán National Park, Quebrada Pachachaca, D.N. Smith 12619, A. Gonzalez & D. Maldonado (USM); Quebrada Los Cedros, D.N. Smith 9892, R. Valencia & L. Minaya (CPUN); Prov. Huari, Huascarán National Park, Alpamayo−Cashampampa trail, 3950 m, 13 Mar 1985, D.N. Smith 10054 & R. Valencia (MO, USM); Prov. Recuay, between Pequipalpa and Rachacoco, L.I. Masías 44 (USM); Huascarán National Park, Auqispuquipio, D.N. Smith 12115, R. Valencia & M. Buddensiek (MO, USM); Prov. Yungay, Cordillera Blanca, 20 km ENE of Yungay, 9°6'26.2"S, 77°41'8.2"W, 3247 m, 15 Mar 2008, P.M. Peterson 21703, R.J. Soreng, M. La Torre & J.V. Rojas Fox (US, USM); quebrada Llauganuco, 24 km ENE of Yungay, 9°5'46.3"S, 77°40'19.3"W, 3309 m, 16 Mar 2008, P.M. Peterson 21720, R.J. Soreng, M. La Torre & J.V. Rojas Fox (US, USM). **Arequipa**: Prov. Caraveli, Quicacha, 5 May 1955, A. Guevara s.n. (USM). **Ayacucho**: Prov. Huanca Sancos, 19 km NW of Putajasa on road towards Huanca Sancos, 14°00'2.6"S, 74°17'14.1"W, 3900 m, 25 Feb 2002, P.M. Peterson 16268, A. Cano, M. La Torre, A Ramirez & D. Susanibar (US, USM). **Cajamarca**: Prov. Cajabamba, ca. 20 km WSW of Cajabamba, 7°42'18.0"S, 78°14'1.5"W, 3263 m, 23 Mar 2008, P.M. Peterson & R.J. Soreng 21868 (US, USM); Prov. Cajamarca, La Encañada, Cerro Negro, M. Cabanillas Medina & M. Sánchez Montoya 1530 (CPUN); La Encañada, ruta Combayo−Yanacocha, I. Sánchez Vega 10574 (CPUN); N del canal Cumbe Mayo, I Sánchez –Vega U. Molau 3733 (CPUN); Cumbe Mayo, W of Cajamarca, S.A. Renvoize 4990, S. Lægaard & I. Sánchez Vega (CPUN, K, US); Pampas de Guanico, ruta a Guagal, I. Sánchez Vega 1141 (CPUN); Sais Atahualpa, de Porcón, I Sánchez Vega 2528, V. Torrel & E. Medina (CPUN); Quebrada de la Esperanza along road to Cumbe Mayo, I. Sánchez Vega & V. Torre 3289 (CPUN); Cerro Campanario, I. Sánchez Vega 6605 (CPUN); Prov. Celendin, Celendin, 3600 m, 24 Jun 1961, R. Ferreyra 15117 (USM); Prov. San Miguel, 64 km N of Cajamarca and 3 km SW of El Cobro, 3530 m, 16 Mar 2000, P.M. Peterson & N. Refulio Rodriguez 14926 (CPUN, US, USM); Prov. San Pablo, 32 km NW of Cajamarca towards San Pablo, 3470 m, 15 Mar 2000, P.M. Peterson & N. Refulio Rodriguez 14882 (US, USM); Cajamarca−Chota road, km 37−40 marker, A. Gentry 61575, C. Diaz & C. Blaney (MO, USM). **Huancavelica**: Prov. Huancavelica, Canyon of Rio Ichu, SW of Huancavelica, 12°48'48.2"S, 75°3'24.5"W, 4894 m, 14 Mar 2007, P.M. Peterson 20462, R.J. Soreng, K. Romaschenko & D. Susanibar (US, USM); Tayacaja, Hacienda Tocas, between Colcabamba and Paucarbamba, 3400 m, 20 Apr 1954, O. Tovar 1981 (US, USM). **Huánuco**: Prov. de Huánuco, Mitotambo, above Mito, R. Ferreyra 6895 (MOL, US, USM); above Mitotambo, R. Ferreyra 9434 (USM); 3−6 mi NW of Mito, J.F. Macbride and Featherstone 1929 (F, US), Mito, J.F. Macbride and Featherstone 1721 (F, US); Prov. Ambo, Huarmiragra, A. Granda 1676 (USM). **Junín**: Pachacaya−Huari, 3600 m, 25 Mar 1979, O. Tovar 7838 (USM); Prov. Huancayo, Cerros E of Huancayo, O. Tovar 3259 (US); above Ocopilla, near Huancayo, O. Tovar 4506 (US, USM); above Huancayo, O. Tovar 2795 (US, USM); Palián, O. Tovar 9339 (USM); Cerros E of Huancayo, O. Tovar 3259 (USM); Oroya, A.S. Hitchcock 22182 (US). **La Libertad**: Prov. Huancayo, Acopalca, 4000 m, 20 Jul 1945, J. Infantes Vera 406 (LIL); Prov. Bolivar, 3 km ESE of Longotea and 9 km W of Uchumarca, 7°3'27.9"S, 77°51'2.2"W, 3202 m, 31 Mar 2008, P.M. Peterson 21957, R.J. Soreng & J. Montoya Quino (US, USM); Prov. Otuzco, Trujillo−Huamachuco road, 10−15 km before Shorey, D.N. Smith & R. Vasquez 3289 (MO, USM); Larbimbambu, 2450 m, 5 Feb 1949, J. Infantes Vera 1654 (LIL); Cerro Sango, A. Sagástegui 14428 (HAO); Prov. Santiago de Chuco, Shoreyo−Truillo road, 5 km from Shoreyo, D. Smith 2339 (MO, US, USM). **Lambayeque**: Prov. Ferreñafe, Incahuasi, S. Llatas Q. 1934 (CPUN, HAO, USM). **Piura**: Prov. Huancabamba, 14 km E of Sondor on road towards Tabaconas, 2540 m, 31 Mar 2000, P.M. Peterson & N. Refulio Rodriguez 15153 (CPUN, US, USM). **Puno**: Chijnaya, Pueblo de Pucará, 3900 m, Mar 1964, A. Vera 107 (COL); Hacienda Caracara, entre Llave y Pucará, 3850 m, 4 Mar 1966, O. Tovar 5162 (US); Hacienda Sollocota, 3850 m, 7 Mar 1966, O. Tovar 5229 (USM); Camacani, 9 Mar 1966, 3850 m, O. Tovar 5263 (USM); Prov. Carabaya, 10 km S of Ollachea, J.D. Boeke & S. Boeke 2974 (NY, US); Prov. Chucuito, 3 km S of Pizacoma, 4050 m, 7 Mar 1999, P.M. Peterson & N. Refulio Rodriguez 14694 (US, USM).

#### 7. Muhlenbergia coerulea

Taxon classificationPlantaePoalesPoaceae

(Griseb.) Mez × M. rigida (Kunth) Kunth.

Fig. 5E−K

Podosemum angustatum J. Presl, Reliq. Haenk. 1(4–5):229. 1830. Muhlenbergiaangustata (J. Presl) Kunth, Enum. Pl. 1: 202. 1833. Type: Peru, *Haenke s.n.* (holotype: PR-4657!; isotype: PRC-450958 [image!]).
Muhlenbergia phragmitoides Griseb. var. breviaristata Hack., Annuaire Conserv. Jard. Bot. Genève 17: 291. 1914. Muhlenbergiabreviaristata (Hack.) Parodi, Physis (Buenos Aires) 9: 219. 1928. Type: Argentina, Tucumán, Lara, 3200 m s.m., 28 Mar 1912, *M. Lillo 11322, Herb. T. Stuckert 22470* (holotype: W-1916-0039607!; isotypes: LIL-40186!, US-3412353 fragm. ex W!).
Muhlenbergia holwayorum Hitchc., Contr. U.S. Natl. Herb. 24(8):389. 1927. Type: Bolivia, Sorata, 16 Apr *1920, E.W.D. Holway & M. M. Holway 530* (holotype: US-1108445!).

##### Description.

*Panicles* (−0.6)1−4 cm wide, spike-like with lower branches slightly prolonged, erect, plumbeous to reddish-purple. *Spikelets* plumbeous to reddish-purple, scabrous; *glumes* 2.5−3.5(−4) mm long, ½ to ¾ as long as the floret; *lemmas* 4−6 mm long, awned, the awns generally 3−8(−10) mm long, straight.

##### Distribution.

This presumed hybrid is known to occur in Argentina, Chile, Colombia, Ecuador, Peru and Venezuela.

##### Ecology.

The hybrid occurs on gravelly slopes and near cultivated fields in many different plant communities between 2200−3800 m in association with *Alnus*, *Aristidaadscensionis* L. *Baccharis*, *Berberis*, *Calceolaria*, *Cenchrusclandestinus*, *Eragrostispastoensis* (Kunth) Trin., *Festuca*, *Jarava*, *Lantana*, *Lupinus*, *Muhlenbergiacenchroides*, *M.phalaroides*, *M.rigida*, *Oxalis*, *Polygala*, *Puya*, *Schinusmolle* L., *Schizochyrium* and *Solanum*.

##### Comments.

As expected, individuals of *M.coerulea* × *M.rigida* have an intermediate morphology, such as: lemma awn length [3−8 (−10) mm], glume length (1/2−3/4 as long as the lemma) and spikelet colouration (plumbeous to reddish-purple). In the Flora of Ecuador, Lægaard and Peterson (2001) recognised two intermediate forms (“intermediate” and “subangustata”) based on lemma awn length, glume length, colour of the spekelets and surface scabrosity of the glumes and lemmas.

There are no molecular studies investigating the population genetics of the hybrid with both presumed parents but we hope to persue this in the future. In our analysis, two samples of *M.coerulea* from Peru form a pair with little variation and two samples of *M.rigida*, one from Mexico and the other from Peru, form a pair with little variation in the analysis of seven DNA sequence markers (Peterson et al. 2010b). Each of these two clades of *M.coerulea* and *M.rigida* were not paired but were placed along a comb-like grade in the M.subg.Trichochloa clade. In our new analysis, there are more samples of *M.rigida* from Mexico and there is greater genetic variability among individuals of this species (Fig. 1). However, overall there appears to be low levels of divergence among species in M.subg.Trichochloa, hence the comb-like appearance suggesting rapid speciation (Peterson et al. 2010b).

##### Specimens examined.

Peru. **Ancash**: 10 km towards Casma, S.A. Renvoize & S. Lægaard 5154 (CPUN); E of Yungay, S.A. Renvoize & S. Lægaard 5058 (CPUN); Prov. Asunción, Chacas, A. Cano 14572, M. La Torre & W. Mendoza (USM); Prov. Bolognesi, above Chiquián, E. Cerrate 1549 (US, USM); Prov. Corongo, Ocshmarca, S. Leiva 51 (USM); Prov. Huaylas, Huascarán National Park, Auquispuquio ruins, D.N. Smith 11960, R. Valencia & M. Buddensiek (CPUN, USM); Huascarán National Park, Pueblo Libre, A. Cano 11619 (USM); Pueblo Libre, Antircán, A. Cano 11714 (USM). **Apurimac**: Prov. Abancay, 48 km SW of Abamcay on road towards Andahuaylas, 13°42'23.6"S, 72°57'20.3"W, 3280 m, 21 Mar 2002, P.M. Peterson & N. Refulio Rodriguez 16650 (US, USM). **Ayacucho**: Prov. Huamanga, 23 km S of Ayacucho on hwy 3 towards Abancay, 13°16'21.5"S, 74°13'46.8"W, 3575 m, 15 Mar 2007, P.M. Peterson 20490, 20496, R.J. Soreng, K. Romaschenko & D. Susanibar Cruz (US, USM); 27 km S of Ayacucho on hwy 3 towards Abancay, 13°17'9.8"S, 74°13'41.9"W, 3684 m, 16 Mar 2007, P.M. Peterson 20502, R.J. Soreng, K. Romaschenko & D. Susanibar Cruz (US, USM); Prov. Huanca Sancos, 23 km NW of Putajasa on road towards Huanca Sancos, 13°58'11.1"S, 74°18'9.0W, 3800 m, 25 Feb 2002, P.M. Peterson 16272, A. Cano, M. La Torre, A Ramirez & D. Susanibar (US, USM); Prov. Huanca Sancos, 27 km NW of Putajasa and 3 km S of Sacsamarca, 13°57'51.1"S, 74°18'41.5"W, 3650 m, 25 Feb 2002, P.M. Peterson 16273B, A. Cano, M. La Torre, A Ramirez & D. Susanibar (US, USM); Prov. Lucanas, 13 km E of Puquio at km 172 marker, 14°41'18.5S, 74°4'26.8W, 3730 m, 8 Apr 2004, P.M. Peterson & N. Refulio Rodriguez 18207 (US, USM); Ancamarca, L. Vargas & G. Mora 337 (USM). **Cajamarca**: Prov. Cajamarca, 12 km N of Cajamarca on road toward Bambamarca, 3020 m, 14 Mar 2000, P.M. Peterson & N. Refulio Rodriguez 14860 (CPUN, US, USM); Cajamarca−Bambamarca, 13−18 km from Cajamarca, D.N. Smith & R. Vasquez M. 3441 (MO, US, USM); Chotén, 4 km de la carretera Pacasmayo, I. Sánchez Vega 2570, V. Torrel & E. Medina (CPUN); Porcón, I. Sánchez Vega 51 (CPUN, US); above La Encañada ruta a Celendín, I. Sánchez Vega & W. Ruiz Vigo 1730 (CPUN); Prov. Celendín, La Tranca, I. Sánchez Vega & W. Ruiz Vigo 1815 (CPUN); Prov. Celendín, 4 air km W of Celendín, 6°54'13.5"S, 78°10'8.4"W, 2687 m, 29 Mar 2008, P.M. Peterson 21921 & 21922, R.J. Soreng & J. Montoya Quino (US, USM); 10 km from Celendín towards Rio Marañon, S.A. Renvoize & S. Lægaard 4883 (CPUN); Celendín to Cajamarca, near Quillimbas, S.A. Renvoize & S. Lægaard 4871 (CPUN); Cerro Conturami 10 km from Celendín, S.A. Renvoize & S. Lægaard 4946 (CPUN); Prov. Contumaza, Tres Cruces, A. Sagástegui & M. Fukushima 5119 (US); Celendín−Balsas road, 3−10 km from Celendín, D.N. Smith & I. Sánchez Vega 4300 (MO, US). **Cusco**: Prov. Calca, 10 km N of Calca on road towards Lares, 13°16'2.9"S, 71°55'20.1W, 3490 m, 17 Mar 2002, P.M. Peterson & N. Refulio Rodriguez 16597 (US, USM); Prov. Santo Tomas, Chumbivilcas, Apr 1982, M.E. Tapia 208 (USM). **Huancavelica**: Motcca, cerca a Conaica, 3500 m, 1 Apr, 1967, O. Tovar 5889 (USM); Prov. Huancavelica, Pararpuquio, below Conaica, O. Tovar 169 (MOL, US, USM); Sachahuajta, 7 km to Conaica, O. Tovar 950 (US, USM); near Yauli, O. Tovar 2996 (US, USM); Yacu−huanay, O. Tovar 3695 (USM, US); Prov. Tayacaja, Andaimarca, between Colcabamba and Surcubamba, O. Tovar 1821 (US, USM). **Huánuco**: Prov. de Huánuco, Mitotambo above Mito, R. Ferreyra 9477 (US, USM); Mito, J.F. Macbride 3320 (F, US). **Junín**: Prov. Huancayo, Paccba, 20 km S of Huancayo, O. Tovar 3294 (US, USM); Cerro La Libertad, O. Verlarde Nuñez 2637 (MOL). **La Libertad**: Prov. Bolivar, above Longotea, ruta Bolivar, I. Sánchez Vega 5015 (CPUN); Prov. Otuzco, Agallpampa, A. López M. 1041 (US); Trujillo−Huamachuco road, D.N. Smith & R. Vasquez 3309 (MO, USM). **Lambayeque**: Prov. Ferreñafe, Inkawasi, S. Llatas Quiroz 4114 (USM). **Lima**: Prov. Canta, 2 km SE of San Jose Canta, 11°30'2.0"S, 76°40'23.4W, 2232 m, 27 Mar 2004, P.M. Peterson & N. Refulio Rodriguez 17984 (US, USM). **Piura**: 14 km E of Sondor on road towards Tabaconas, 2540 m, 31 Mar 2000, P.M. Peterson & N. Refulio Rodriguez 15160 (CPUN, US, USM).

#### 8. Muhlenbergia diversiglumis

Taxon classificationPlantaePoalesPoaceae

Trin., Mém. Acad. Imp. Sci. Saint-Pétersbourg, Sér. 6, Sci. Math., Seconde Pt. Sci. Nat. 6,4(3–4):298. 1841.

Fig. 7A−D

##### Type.

Mexico, Porto Pedro, *Karwinsky 1393* (holotype: LE-TRIN-1497.01!; isotypes: US-84831! fragm. ex LE-TRIN-1497.02, W!).

##### Description.

Sprawling *annuals*. *Culms* 16–50 cm tall, decumbent, rooting at the lower nodes; *nodes* retrorsely pilose; *internodes* smooth or scabridulous. *Leaf sheaths* 1.5–8.5 cm long, sparsely or densely pilose, hairs to 3 mm long, papillose-based; *ligules* 0.5–0.8 mm long, membranous, apex truncate, erose; *blades* 2–6 cm long, 1.5–4 mm wide, flat, bases distinctly narrowed to the junction with the sheath, surfaces scabridulous and sparsely pilose, hairs papillose-based. *Panicles* 6–10.5 cm long, 2.0–4.5 cm wide, secund, open; *primary branches* 0.8–3.5 cm long, secund, spreading at right angles or somewhat reflexed usually lying to one side with 2–5 spikelets; *secondary branches* not developed; *pedicels* 1–5 mm long, scabrous or shortly pilose, hairs papillose-based; *disarticulation* at the base of the primary branches where there is a weak and contorted stipe. *Spikelets* 4–8 mm long, dimorphic with respect to the glumes, proximal spikelets on each branch almost sessile; *glumes of proximal spikelets* on each branch subequal, 0.2–0.7 mm long, orbicular, truncate, often erose or irregularly toothed, unawned; *glumes of distal spikelets* on each branch markedly unequal; *lower* glumes to 8 mm long, 1-veined, acute, usually awned, awns 0.5–3 mm; *upper* glumes orbicular, acute, sometimes awn-tipped; *lemmas* 4.0–7.6 mm long, linear to broadly lanceolate, light greenish, smooth or scabrous, usually with greenish veins, apices acuminate, awned, awns 6–19 mm long, usually straight, scabrous; *paleas* 3.7–6.8 mm long, narrowly lanceolate, coarsely papillate or almost smooth, 2-keeled, the veins prominent, scabrous, greenish, sometimes extending as minute awns, acuminate; *anthers* 0.4–0.8 mm long, yellowish. *Caryopses* 1.8–3 mm long, oblong-ovoid, flattened, brownish. 2*n* = 20.

**Figure 7.**
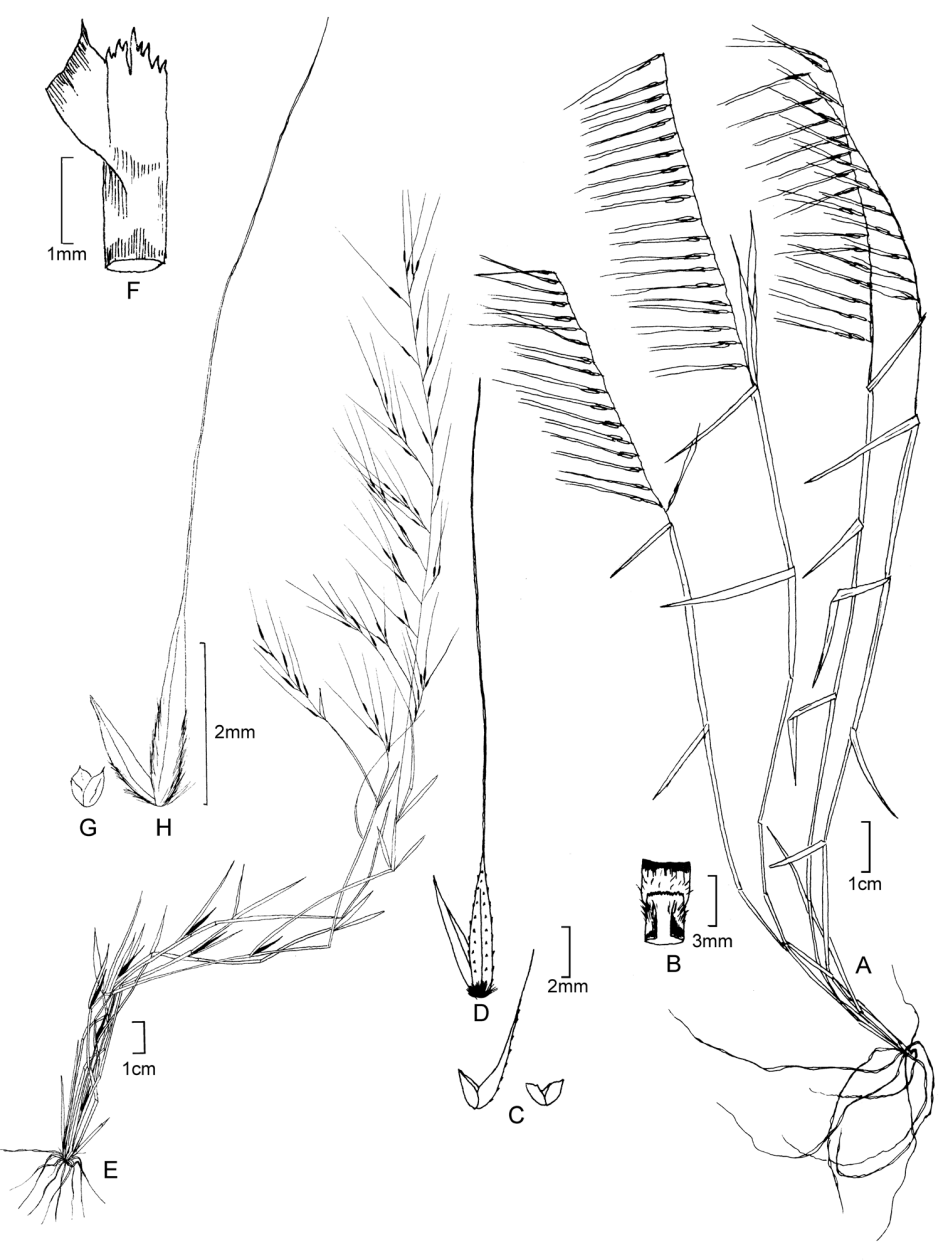
**A−D***Muhlenbergiadiversiglumis* Trin **A** habit **B** ligule **C** glumes **D** floret **E−H***Muhlenbergiamicrosperma* (DC.) Kunth **E** habit **F** ligule **G** glumes **H** lemma. Drawings from Peterson and Annable (1991)**A−D** drawn from *P.M. Peterson & C.R. Annable 4158* (WS) **F−H** drawn from *P.M. Peterson & C.R. Annable 4185* (WS).

##### Distribution.

The species is native to North America, Central America, Colombia, Venezuela, Ecuador, Peru, and Argentina.

##### Ecology.

*Muhlenbergiadiversiglumis* grows on moist cliffs, along water courses, sandy slopes, and road cuts, primarily in moist shaded environments of broadleaf evergreen forests and pine-oak forests, at elevations of 600–2500 m.

##### Comments.

*Muhlenbergiadiversiglumis* can be differentiated from *M.ciliata*, *M.microsperma*, and *M.romaschenkoi* in having secund *panicles* (versus not secund in the latter three species) with each primary branch consisting of 2–5 dimorphic spikelets where the proximal spikelets have short orbicular glumes less than 1 mm long, and the distal spikelets have glumes up to 8 mm long (Peterson and Annable 1991; Giraldo-Cañas and Peterson 2009).

*Muhlenbergiadiversiglumis* is a member of M.subg.Muhlenbergia and is sister to *M.alamosae* Vasey and together they are sister to the *M.tarahumara*−*M.cenchroides*−*M.bryophilus*−*M.uniseta* clade (Fig. 1B).

##### Specimens examined.

Peru. **Amazonas**: Dist. Vista Alegre, Puente sobre el Río Salas, 1525 m, 30 Jun 1998, I.M. Sánchez Vega, M.O. Dillon & M. Zapata 9580 (HAO, MO). **Ayacucho**: Naranjayoc, entre Ayna y el Río Apurimac, 900 m, 16 May 1969, O. Tovar 6136 (USM); Ayna, cerca entre Tambo y San Francisco, Jan 1969, L. Aucasime 428 (USM); Province La Mar, Machente, entre Yanamonte y Ayna, 1200 m, 16 May 1969, O. Tovar 6126 (USM); Province La Mar, entre Ayna & Río Apurimac, 900 m, 16 May 1969, O. Tovar 6136 (USM); Ayna, between Huanta and Río Apurimac, 750 m, 7 May 1929, E.P. Killip & A.C. Smith 23127 (US). **Cusco**: Urubamba Valley, San Miguel, 1800 m, 28 May 1913, O.F. Cook & G.B. Gilbert 958 (US); Urubamba, Ciudadela Macchu Picchu, 2300 m, 11 Apr 1963, C. Vargas C. 2159 (US), 14355 (US, USM); Urubamba, Macchu Picchu, 2040 m, Mar 1949, F. Marin 1381 (US); Urubamba, 110 km de Cusco en el camino y atrás de las aguas termales con plantaciones de palta, Macchu Picchu, 2050 m, 21 May 1988; G. Valencia & R. Ochoa 9147 (MO, US). **Huánuco**: Province Leoncio Prado, La Divisoria, carretera a Pucallpa, 1600 m, 21 Apr 1989, J. Schunke Vigo 11349 (MO, US, USM). **Piura**: Huancabamba, Porculla, 1770 m, 6 Mar 1989, S. Llatas Quiroz 2431 (CPUN, HAO, MO).

#### 9. Muhlenbergia fastigiata

Taxon classificationPlantaePoalesPoaceae

(J. Presl) Henrard, Meded. Rijks-Herb. 40: 59. 1921.

Fig. 8A−E

Sporobolus fastigiatus J. Presl, Reliq. Haenk. 1 (4–5): 241. 1830. Type: Peru, *Haenke s.n.* (holotype: PR, isotypes: PRC-450197 [image!], US-3048470! fragm. ex PR).
Muhlenbergia cleefii Lægaard, Caldasia 17 (82–85): 409–411. 1995. Type: Colombia, Boyacá, Sierra Nevada del Cocuy, Alto Valle de Lagunilla, páramo pantanoso al sur de la Laguna Cuadrada, 4060 m, 26 sep 1972, *A. Cleef & P.A. Florschutz 5578* (holotype: COL!; isotypes: U-0007997 [image!], US-2785756!).

##### Description.

Dense mat-forming *perennials* with scaly rhizomes, the acute scales 4.5−8.1 (−10) mm long, the rhizomes 1−2 (−2.5) mm in diameter. *Culms* 2−8 (−11) cm long, mostly erect, 2−3 (4) mm diameter below; *internodes* 1−10 mm long, compressed. *Leaf sheaths* 3−6 (−10) mm long, distichous, overlapping, margins hyaline; *ligules* 6−1.4 mm long, membranous, decurrent, apex acute to obtuse; *blades* 2.5−8.5 mm long, 0.6−1.1 mm wide, involute, arcuate, glabrous to finely papillose, margins scabrous, apex navicular, often pungent. *Panicles* 1−2 cm long, 1−5 mm wide, contracted, narrow, usually exserted with 3−9 nodes; *primary branches* 0.4−1 cm long, ascending, solitary at a node; *rachis* papillose-roughened just below the spikelets. Spikelets (1.8−-) 2−2.5 mm long, plumbeous; *glumes* (0.8−) 1−1.6 mm long,about ½ as long as the floret, subequal, 1-veined, rarely 2-veined, apex acute to obtuse; *lemmas* (1.7−) 1.9−2.4 mm long, 3-veined, glabrous, plumbeous, apex acute, mucronate, the mucros less than 0.3 mm long; *paleas* (1.6−) 1.8−2.4 mm long, 2-veined, glabrous; *anthers* 1−1.7 mm long, yellow or purple. *Caryopses* 1−1.3 mm long, about 0.4 mm wide, ellipsoid, dark brown.

**Figure 8.**
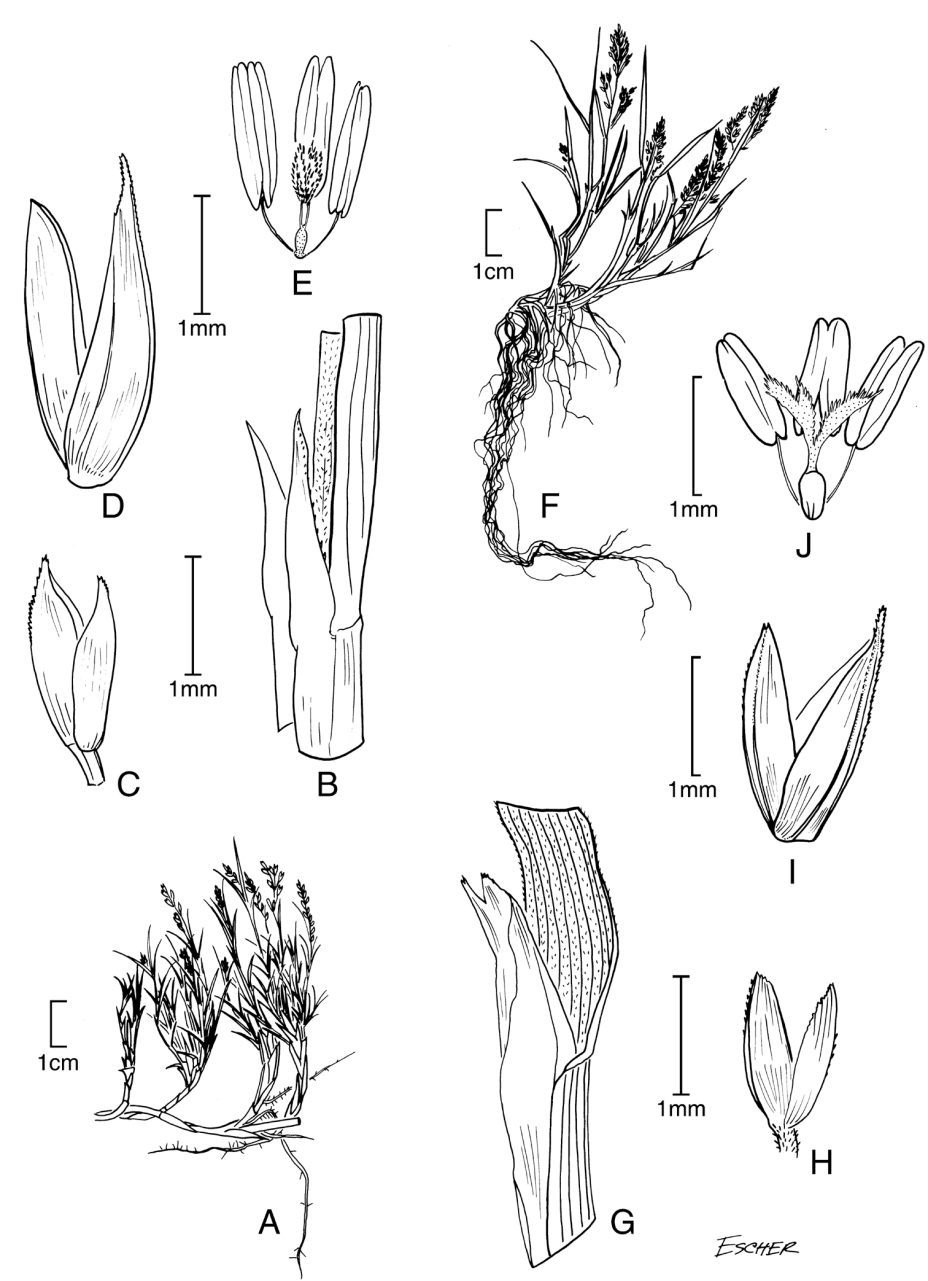
**A−E***Muhlenbergiafastigiata* (J.Presl) Henrard **A** habit **B** ligule **C** glumes **D** floret **E** stamens and pistil **F−J***Muhlenbergialigularais* (Hack.) Hitchc **F** habit **G** ligule **H** glumes **I** floret **J** stamens and pistil. Drawings from Giraldo-Cañas and Peterson (2009), Peterson and Giraldo-Cañas (2011)**A−E** drawn from *P.M. Peterson, S. Lægaard, R.J. Soreng & C.R. Annable 12709* (US) **F−J** drawn from *P.M. Peterson, S. Lægaard, R.J. Soreng & C.R. Annable 12684* (US).

##### Distribution.

*Muhlenbergiafastigiata* is found in South America in the Andean highlands of Argentina, Bolivia, Chile, Colombia, and Peru (Morden 1985; Morden and Hatch 1996).

##### Ecology.

*Muhlenbergiafastigiata* occurs on grassy flats, playas, slopes, rock outcrops, near gravelly creeks and lake margins in granitic and calcareous soils associated with *Baccharis*, *Berberis*, *Calamagrostis*, *Ephedra*, *Festuca*, *Jarava*, *Margyricarpus*, *Muhlenbergia*, *ligularis*, *M.peruviana*, and *Nassella*; 3000−4900 m.

##### Comments.

Morphologically, *M.fastigiata* can sometimes be confused with the distantly related *M.ligularis* (M.subg.Bealia; Fig. 1B). However, the former has well developed rhizomes (absent in *M.ligularis*) and tightly involute, acuate leaf blades (versus flat and straight). *Muhlenbergiarichardsonis* (Trin.) Rydb., a wide ranging North American species, is also morphologically similar to *M.fastigiata* in most characters, although *M.richardsonis* has a taller habit and differes in leaf anatomy (Morden 1985; Morden and Hatch 1996). *Aciachneacicularis* Lægaard and *A.pulvinata* Benth., two small, compact, mat-forming perennials, can also be mistaken for *M.fastigiata*. However, both species of *Aciachne* (Pooideae: Stipeae) have longer spikelets ranging from 3.8−6 mm long, glumes that are 3−5-veined, and tightly overlapping lemma margins (Renvoize 1998; Giraldo-Cañas and Peterson 2009).

*Muhlenbergiafastigiata* is sister to the North American, *M.richardsonis* in M.subg.Pseudosporobolus [Fig. 1; Peterson et al. 2010b].

##### Specimens examined.

Peru. **Ancash**: Recuay, Cordillera Blanca, 39 km S of Catac on road towards Raquia, 3980 m, 21 Mar 1997, P.M. Peterson 13825 & N. Refulio Rodriguez (US, USM); Cordillera Negra, E slope of Cerro Pullicharca (Purac), ca. 1.5 km W of hwy 109 and then 3.6 km S of Tapacocha along Rio Santa, 9°55'39.4"S, 77°23'45.3"W, 3822 m, 9 Mar 2008, P.M. Peterson 21512, R.J. Soreng, M.I. La Torre & J.V. Rojas Fox (US, USM); between Santa Rosa and Araranca 12 Apr 1915, O.F. Cook & G.B. Gilbert 168 (US). **Arequipa**: Prov. Caylloma, Valle de Colca, Chiray−Tuti, 3900 m, 12 Jan 1999, S.G. Beck 26354 (LPB); Prov. Caylloma, Pampa Cañahuas, “Grama dulce”, 4200 m, 3 Jul 2001, M. Rodríguez Diaz 1130 (USM). **Cajamarca**: Prov. Hualgayoc, 8 km NE of Hualgayoc on hwy 3N towards Bambamarca, 3080 m, 17 Mar 2000, P.M. Peterson & N. Refulio Rodriguez 14950 (CPUN, US, USM). Kanzel, above Sunchubamba, 3680 m, 6 Aug 1957, H. Ellenberg 1852 (US). **Cusco**: Chumbivilias, Rio de Velille, 3850 m, C. Vargas C. 14084 (US); Espinar, alrededores de Yauri, 3900 m, 26 Mar 1956, 26 Mar 1956, C. Vargas C. 11209 (US). **Huancavelica**: Huancavelica, Canyon of Rio Ichu, SW of Huancavelica ca. 5 km on Hwy 3 to Santa Ines and Ayacucho, 12°48'48.2"S, 75°3'24.5"W, 4894 m, 14 Mar 2007, P.M. Peterson 20463, R.J. Soreng, K. Romaschenko & D. Susanibar Cruz (US, USM); Tayacaja, Just above Chuquitambo, 4 km SW of Pazo, 3975 m, 9 Apr 1997, P.M. Peterson 14151 & O. Tovar (US, USM). **Junín**: Prov. Huancayo, Acopalca, 3900 m, 25 Jul 1945, J. Infantes Vera s.n. (LIL); Prov. Huancayo, Acopalca, 4000 m, 20 July 1947, J. Infantes Vera 446 (LIL); entre Tarma y La Oroya, 3700 m, A. Weberbauer 2525 (MOL); Hacienda Cachi-Cachi, 4000 m, 22 Oct 1950, O. Velarde Nuñez 2869 (MOL); Jauja, 34 km SE of La Oroya on road towards Jauja, 3600 m, 9 Apr 1997, P.M. Peterson 14125 & O. Tovar (US, USM); Jauja, Tarma−Jauja road, 28 km from Tarma, 11°35'S, 75°38'W, 3950 m, 10 Jan 1984, D.N. Smith 5694 (MO, US); Junín, SE shore of Lago Chinchay Cocha (Junin) and 8 km S of Huayre, 4110 m, 7 Apr 1997, P.M. Peterson 14096 & O. Tovar (US, USM); Yauli, 156 km W of Lima just above Yauli, 4120 m, 5 Apr 1997, P.M. Peterson 14040 & O. Tovar (US, USM); Yauli, La Oroya, 30 Nov 1924, F.L. Stevens 1 (US); Oroyo, 23 Oct 1923, A.S. Hitchcock 22135 (US), 21 Feb 1954, Hirsch s,n. (US), Stevens 1 (US); entre Tarma y Oroya, 3400 m, 29 Jun 1948, R. Ferreyra 3828 (MOL, US, USM); ca. 24 km W of Tarma, 4050 m, 6 May 1979, Teppner 79/387 (US). **La Libertad**: Prov. Huamachuco, Cangau Sartimbamba, 3200 m, 28 Jan 1952, J. Infantes Vera 4072 (LIL); Prov. Huamachuco, Cangau Sartimbamba, 4000 m, 29 Jan 1952, J. Infantes Vera 4045 (LIL); Prov. Huamachuco, Huailillas, 3800 m, 8 Aug 1951, J. Infantes Vera s.n. (LIL). **Lima**: Provincia Huarochiri, Chilca, Bella Vista, 3800 m, 5 Jun 1940, E. Asplund 11436 (NY, US); road from Lima to Huancayo, 3300 m, 20 May 1981, G. Sullivan, K. Young, S. Sánchez & D. Soejarto 1021 (MO, US). **Pasco**: Pasco, 8 km W of NW arm of Laguna de Junín, 10°55'51.9"S, 76°18'8.6"W, 4141 m, 5 Mar 2007, P.M. Peterson 20313, R.J. Soreng & K. Romashchenko (US, USM). **Puno**: Chucuito, 3 km NE of Zepita on road towards Copani, 3820 m, 5 Mar 1999, P.M. Peterson 14631, N. Refulio Rodriguez & F. Salvador Perez (US, USM); 20 km E of Huacullani on road towards Desaguadero, 3880 m, 5 Mar 1999, P.M. Peterson 14645, N. Refulio Rodriguez & F. Salvador Perez (US, USM); 5 km S of Kelluyo, 3860 m, 7 Mar 1999, P.M. Peterson 14686, N. Refulio Rodriguez & F. Salvador Perez (US, USM); El Collao, 11 km E of Santa Rosa and 5 km W of Mazo Cruz, 3880 m, 2 Mar 1999, P.M. Peterson 14598, N. Refulio Rodriguez & F. Salvador Perez (US, USM); 9 km N of Conduriri and 63 km S of Ilave along the Rio Huenque, 3820 m, 3 Mar 1999, P.M. Peterson 14600, N. Refulio Rodriguez & F. Salvador Perez (US, USM); Provincia Melgar, 7 km WNW of Santa. Rosa, 14°35'54.9"S, 70°51'34.7"W, 4002 m, 23 Mar 2007, P.M. Peterson 20605, R.J. Soreng & K. Romashchenko (US, USM); SW of Juliaca ca. 8 km on Hwy 30 to Arequipa, 15°32'37.2"S, 70°12'55.9"W, 3854 m, 31 Mar 2007, P.M. Peterson 20715, R.J. Soreng, K. Romaschenko & S. Gonzalez Elizondo (US, USM); Puno, 2 km N of Laraquerion road towards Puno, 16°6'59.1"S, 70°1'59.1"W, 3940 m, 18 Apr 2004, P.M. Peterson 18327 & N. Refulio Rodriguez (US, USM); San Roman, 4 km E of Santa Lucia, 15°41'4.2"S, 70°34'8.0"W, 4030 m, 19 Apr 2004, P.M. Peterson 18332 & N. Refulio Rodriguez (US, USM); 21 km W of Santa Lucia and 5 km E of Puente Cañuma (Laguna Lagunillas), 15°40'4.1"S, 70°47'4.4"W, 4260 m, 20 Apr 2004, P.M. Peterson 18344 & N. Refulio Rodriguez (US, USM); Camjata Hacienda, Capachica Peninsula, Lake Titicaca, Tutin 975 (NY); between Juliaca and Cusco, 3880 m, 27 Nov 1923, A.S. Hitchcock 22448 (US); Hacienda Pairumani, 20 mi SW of Llave, 3900 m, 21 Jul 1946, O.P. Pearson & A. Pearson 14 (US); between Puno and Laguna Umayo, N of Puno, 3970 m, 12 Sep 1959, H.G. Barclay 9265 (US). **Tacna**: Cordillera Volcan Tacora, Ancara, Werderman 1138 (NY, US); Tarata, 11 km NW of Yabroco on road towards Cano, 3680 m, 16 Mar 1999, P.M. Peterson 14814, N. Refulio Rodriguez & F. Salvador Perez (US, USM).

#### 10. Muhlenbergia flexuosa

Taxon classificationPlantaePoalesPoaceae

Hitchc., Contr. U.S. Natl. Herb. 24(8): 388. 1927.

Fig. 9A−E

##### Type.

Peru, Huacachi Estacion near Muna, summit of rocky crest, 2000 m, 20 May−1 Jun 1923, *J.F. Macbride 3874* (holotype: F-534937!; isotypes: BAA-1622!, BM-000938657 [image!], G-00099373 [image!], K!, LE!, US-1256339!).

##### Description.

Caespitose *perennials*. *Culms* 15−40 cm tall, slender, 0.3−0.4 mm diameter just below the panicle with 6−8 nodes per culm; *internodes* 1−6 cm long near base, glabrous, shiny. *Leaf sheaths* about as long as the internodes below, glabrous; *ligules* 0.3−0.5 mm long, membranous, apex truncate, lacerate; *blades* 2−5 cm long, 1−2.2 mm wide, flat, scaberulous, apex acuminate. *Panicles* 3.5−6.5 (−7) cm long, 0.5−1.5 cm wide, narrow, spiciform, sometimes interrupted below; *primary branches* 0.5−2.5 cm long, tightly apressed to loosely ascending or spreading, not more than 45° from the culm axis. *Spikelets* 2.8−4.2 mm long, yellowish to light brown, erect or drooping; *glumes* 2.8−4 mm long, including mucos or awns, subequal, hyaline to membranous, glabrous, 1-veined, scabrous along the midvein, apex acute, unawned, mucronate or awned, the awns up to 1.3 mm long, straight or loosely flexuous; *lemmas* 2.8−4.2 mm long, hyaline to membranous, 3-veined, scaberulous, lower 1/3 sparsely hairy, apically awned, the awns 14−30 mm long, flexuous, callus hairy, the hairs up to 0.4 mm long; *paleas* about as long as the lemmas, 2-veined, glabrous, apex acuminate, often mucronate; *anthers* 1.3−1.6 mm long, purple. *Caryopses* 1.4−1.6 mm long fusiform, light brown.

**Figure 9.**
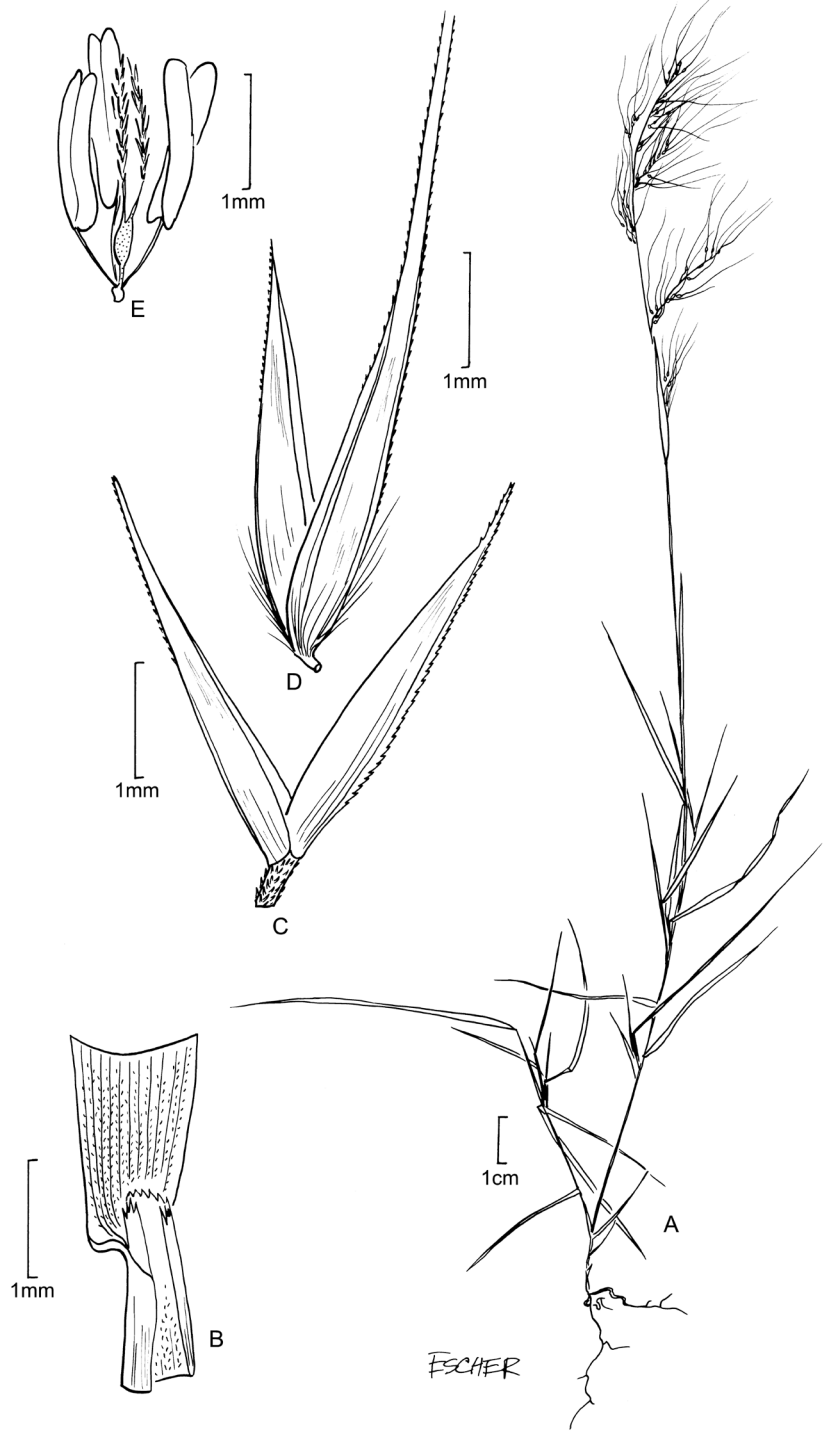
**A−E***Muhlenbergiaflexuosa* Hitchc. **A** habit **B** ligule **C** glumes **D** floret **E** stamens and pistil. Drawn from *H. Teppner 79-293* (US).

##### Distribution.

*Muhlenbergiaflexuosa* endemic to Peru and is known from only a few collections from Huánuco, Pasco and San Martín departments (Hitchcock 1927; Tovar 1993).

##### Ecology.

Steep talus slopes, rocky trail cuts and rock outcropts with *Andropogon*, *Acacia*, *Chusquea*, *Baccharis*, *Puya*, *Salvia*, *Rubus*, *Muhlenbergiaciliata*, *M.beyrichiana*, Acanthaceae and Melastomataceae; 1200−2530 m.

##### Comments.

Hitchcock (1927) suggested *Muhlenbergiaflexuosa* was allied with *M.flaviseta* Scribn. and *M.watsoniana* Hitchc. (≡ *M.scabra* S. Watson), since all have short flat blades, spike-like *panicles* and slender flexuous awns. However, *Muhlenbergiaflaviseta* and *M.watsoniana* are members of the M.subg.Clomena clade while *M.flexuosa* is sister to *M.plumiseta* Columbus−*M.pereilema* P.M. Peterson−*M.beyrichiana* clade, all of which are included in M.subg.Muhlenbergia (Fig. 1; Peterson et al. 2010b). Within *Muhlenbergia*, the derivation of a slender flexuous awn found in *M.flexuosa* has occurred in four of the five subgenera currently recognised within the genus and is only lacking in M.subg.Pseudosporobolus (Fig. 1B; Peterson et al. 2010b).

##### Specimens examined.

Peru. **Huánuco**: Rio Huallaga Cañon, below Rio Santo Domingo, 1316 m, 3 Jun 1923, J.F. Macbride 4205 (US); Pachitea, Canyon of the Rio Grande, 44 air km E of Huánuco, 2 air km ESE of Estación Huacachay (Huacachi), 9°51'34.7"S, 75°50'48.1"W, 1867 m, 7 Mar 2007, P.M. Peterson, R.J. Soreng & K. Romaschenko 20358 (MO, US, USM); 1.5 air km SE of Estación Huacachay (Huacachi), 9°51'42.8"S, 75°50'23.4"W, 2383 m, 8 Mar 2007, P.M. Peterson, R.J. Soreng & K. Romaschenko 20365 (MO, US, USM); along trail 1/3 air km NW of Estación Huacachay (Huacachi), 9°51'1.4"S, 75°49'53.4"W, 2519 m, 8 Mar 2007, P.M. Peterson, R.J. Soreng & K. Romaschenko 20373 (US, USM). **Junin**: Cerro Taldes, Rio Huancabamba, 31 km S of Pozuzo and 3 km N of Llullitunqui, 1370 m, 1 Jun 1979, H. Teppner 79-293 (US). Pasco: Oxapampa, Pozuzo, 21 Jul 1978, Ellemberg 8934 (USM). **San Martín**: Mariscal Caceres, 2350 m, 13 Aug 1986, K. Young 4234 (MO).

#### 11. Muhlenbergia ligularis

Taxon classificationPlantaePoalesPoaceae

(Hack.) Hitchc., Contr. U.S. Natl. Herb. 24(8):388. 1927.

Fig. 8F−J

Sporobolus ligularis Hack., Oesterr. Bot. Z. 52(2):57. 1902. Type: Ecuador, Pichincha, 23 Jan 1899, *Sodiro s.n.* (holotype: W; isotypes: BAA-2905! ex W, US-3274313! ex W, US-1163183!).
Muhlenbergia calcicola Swallen, Contr. U.S. Natl. Herb. 29(9):407. 1950. Type: Guatemala, Huehuetenango, Chemal, Sierra de los Chuchumatanes, 3300 m, 31 Dec 1940, *P.C. Standley 81703* (holotype: US-1910686!; isotypes: F-1200274 [image!], US-2236500US!).
Muhlenbergia breviculmis Swallen, Contr. U.S. Natl. Herb. 29(9):408. 1950. Type: Guatemala, Huehuetenango, Cerro Chemalito, Sierra de los Cuchumatanes, 3.5 mi W of Santa Eulalia, 3100−3150 m, 2 Aug 1942, *J.A. Steyermark 49905* (holotype: US-1935054!; isotypes: F, US-2208654!).
Muhlenbergia minuscula H. Scholz, Willdenowia 14:393. 1984. Type: Bolivia, Canton Ulla-Ulla, Pampa von Ulla-Ulla, Apolobamba Cordillera, 4450 m, 26 Feb 1983, *Menhofer 1974* (holotype: B!; isotype: LPB-0000293 [image!]).

##### Description.

Loosely tufted *annuals* to short-lived *perennials*. *Culms* 2−12 cm tall, 0.2−0.4 mm diameter just below the panicle, erect or decumbent, slender, glabrous, sometimes flowering the first year, up to 15 cm broad, dying in the centre, profusely branched below, a short branchlet with fascicled leaves borne at each node, with 4−6 nodes; *internodes* 2−20 mm long. *Leaf sheaths* 2−20 mm long, generally shorter than the internodes, glabrous, ridged, flattened by the densely fascicled branches; *ligules* 0.6−2.5 mm long, membranous to hyaline, apex truncate to rounded; *blades* 0.3−2.2 cm long, 0.8−1.5 mm wide, flat or folded, prominently veined, thick, firm, usually with whitish-thickened midvein and margins, conspicuously crystalline or spiculate on both surfaces, otherwise glabrous below, sparsely scaberulous above and along margins, tapering to a boat shaped tip. *Panicles* 1.0−3.0 cm long, 0.3−1.4 cm wide, long exserted or included in the uppermost sheath, loosely contracted; *branches* 5−9 mm long, one per node, appressed or reflexed at maturity up to 70° from the culm axis; *pedicels* 1−3 mm long, stiff, densely scabrous, spiculate, erect. *Spikelets* 1.5−3.0 mm long, often plumbeous to reddish-purple; *glumes* 1.0−1.9 mm long, subequal, glabrous, apex acute to obtuse, often minutely erose, greenish-grey; *lower glumes* 1.0−1.7 mm long, 1-veined; *upper glumes* 1.1−1.9 mm long, 1-veined or occasionally 3-veined; *lemmas* 1.5−3.0 mm long, lanceolate, 3-veined, keeled, glabrous, mottled with greenish-black areas or dark greenish mottles on a pale background, apex minutely scaberulous, acuminate, entire or mucronate; mucro rarely more than 1 (−1.2) mm long; *paleas* 1.4−2.9 mm long, lanceolate, glabrous; *anthers* 0.8−1.1 mm long, purplish becoming pale. *Caryopses* 0.8−1.2 mm long, elliptic to fusiform, brownish.

##### Distribution.

This species ranges from Guatemala and Costa Rica (Pohl 1980, at Chirripo Grande) to Colombia, Venezuela, Ecuador, Bolivia, Peru and Argentina.

##### Ecology.

*Muhlenbergialigularis* occurs in grassy flats, moist depressions, wet meadows, gravelly banks, ridgetops and gravelly roadsides often derived from calcarious subtrates associated with *Achnatherum*, *Aciachne*, *Agrostis*, *Alnus*, *Anatherostipa*, *Baccharis*, *Berberis*, *Bidens*, *Buddleya*, *Caiophora*, *Calamagrostis*, *Carex*, *Colletiaspinosissima* J.F. Gmel., *Eleocharis*, *Festuca*, *Gaultheria*, *Hypericum*, *Jarava*, *Juncus*, *Lepidophyllum*, *Lupinus*, *Margyricarpus*, *Muhlenbergia* spp., *Nassella*, *Cenchrusclandestinus*, *Plantago*, *Poa*, *Puya*, *Rumex*, *Salvia* and *Senecio*; 2320−4650 m. Flowering September through January in Central America and February to July in Peru or when moisture is available.

##### Comments.

*Muhlenbergialigularis* is morphologically similar to *M.fastigiata* and can be separated from the latter by possessing flat leaf blades, 0.8−1.5 mm wide and a rather loosely tufted habit without wiry creeping rootstocks and scaly rhizomes. Morphologically, *M.ligularis* differs from *M.caxamarcensis* in having glabrous lemmas (sericious hairs on lower 1/2−3/4 in the latter).

Molecular DNA sequence analysis indicates *M.ligularis* falls within M.subg.Bealia and is polyphyletic (Fig. 1A). Perhaps, cryptic speciation followed by morphological convergence has occurred within *M.ligularis*, also suggested in *Ehrharta* Thund. and *Eriochrysis* P. Beauv. (Verboom et al. 2003; Welker et al. 2016).

##### Specimens examined.

Peru. **Amazonas**: Chachapoyas, 1.5 km al SW of Chachapoyas, 2320 m, 24 May 1962, J.J. Wurdack 504 (US, USM); **Ancash**: Huaraz, Huascaran National Park, Quebrada Rajucolta, 77°22'W, 9°32'S, 4000 m, 17 Apr 1986, D.N. Smith, R. Valencia, M. Buddensiek 12192 (CPUN, MO); S of Huaraz, 28 km from Pachacoto towards La Union, 4500 m, 6 Apr 1988, S.A. Renvoize & S. Lægaard 5118 (CPUN, K); Huari, Cordillera Blanca, 24 km N of Huari on road towards San Luis, 3820 m, 22 Mar 1997, P.M. Peterson & N. Refulio Rodriguez 13871 (MO, US, USM); Huascarán. National Park, Quebrada Pachachaca, a lateral valley of Quebrada Rurichinchay, 77°16'W, 9°77'S, 3700 m, 12 Jun 1986, D.N. Smith, A. Gonzales & D. Maldonado 12562 (CPUN, MO); Prov. Pallasca, Hacienda Chalan, Mar 1949, A. Tito Ureña 7 (MOL); Recuay, Cordillera Blanca, 37 km E of Raquia on Route 02-014 on road towards Huarez, 3900 m, 20 Mar 1997, P.M. Peterson & N. Refulio Rodriguez 13816 (US, USM); 2 km SW of Conococha on Ruta 02-014. 10°8'18.2"S, 77°18'5.4"W, 4107 m, 21 Mar 2004, P.M. Peterson, N. Refulio Rodriguez, A. Cano, M. La Torre & I. Salinas 17902 (MO, US, USM); 3 km E of Conococha, 10°6'55.5"S, 77°16'20.2"W, 4050 m, 24 Mar 2004, P.M. Peterson & N. Refulio Rodriguez 17935 (MO, US, USM); Cordillera Negra, E slope of Cerro Pullicharca (Purac), ca. 1.5 km W of hwy 109 and then 3.6 km S of Tapacocha along Rio Santa, 9°55'39.4"S, 77°23'45.3"W, 3822 m, 9 Mar 2008, P.M. Peterson, R.J. Soreng, M. La Torre & J. Rojas Fox 21513 (US, USM); Cordillera Negra, 30 km from Huaraz towards Casma, Punta Callán, 4200 m, 7 Apr 1988, S.A. Renvoize & S. Lægaard 5162 (CPUN, K); Sihaus, 31 km N of Pomabamba on road towards Sihaus, 3780 m, 24 Mar 1997, P.M. Peterson & N. Refulio Rodriguez 13906 (MO, US, USM); Quebrada Huanca, 77°23'W, 9°55'S, 3950 m, 2 Jul 1985, D.N. Smith & M. Buddensiek 10970 (CPUN, MO); Yungay, W slope of Cordillera Blanca, Rio at E end of Lago Llanganuco, ca. 29 km ENE of new city of Yungay, Parque Nacional Huascaran, Sector Llanganuco, 9°4'8.9"S, 77°38'25.2"W, 3663 m, 16 Mar 2008, P.M. Peterson, R.J. Soreng, M. La Torre & J. Rojas Fox 21747 (US, USM); W slope of Cordillera Blanca on road to pass E of Lago Llanganuco and Yanama, ca. 37 km ENE of new city of Yungay, Parque Nacional Huascaran, Sector Llauganuco, 9°3'16.6"S, 77°35'57.6"W, 3931 m, 16 Mar 2008, P M. Peterson, R.J. Soreng, M. La Torre & J. Rojas Fox 21758 (US, USM); 37 km E of Yungay, 4200 m, 5 Apr 1988, S.A. Renvoize & S. Lægaard 5080 (CPUN, K). **Apurimac**: Aymaraes, 25 km NE of Chalhuanca on road towards Yanaca, at Paseo., 14°15'30.6"S, 73°12'6.9"W, 4300 m, 13 Mar 2002, P.M. Peterson & N. Refulio Rodriguez 16504 (US, USM). **Arequipa**: Caylloma, 15 km SE of Callalli on road towards Condoroma, 15°28'14"S, 71°22'26.9"W, 4610 m, 12 Apr 2004, P.M. Peterson & N. Refulio Rodriguez 18279 (MO, US, USM). **Ayacucho**: Huamanga, S of Ayacucho ca. 45 km on Hwy 3 toward Abancay, 13°20'27"S, 74°9'34.7"W, 4207 m, 16 Mar 2007, P.M. Peterson, R.J. Soreng, K. Romashchenko & D. Susanibar Cruz 20522 (US, USM); 68 km SW of Ayacucho on road towards Pisco, 13°20'5"S, 74°36'36.7"W, 4045 m, 22 Mar 2002, P.M. Peterson & N. Refulio Rodriguez 16659 (US, USM); Lucanas, 2 km SW of Putajasa, 14°8'15.9"S, 74°11'18.4"W, 3900 m, 24 Feb 2002, P.M. Peterson, A. Cano, M. La Torre, A. Ramírez & D. Susanibar Cruz 16225 (US, USM); 25 km E of Puquio on road towards Cusco, 14°37'40.3"S, 74°2'19.1"W, 4150 m, 12 Mar 2002, P.M. Peterson & N. Refulio Rodriguez 16454 (US, USM); 25 km NE of Villatambo on road towards Lucanas at Reserva “Pampa Galeras”. 14°40'46.2"S, 74°23'59.2"W, 3805 m, 8 Apr 2004, P.M. Peterson & N. Refulio Rodriguez 18179 (US, USM); 13 km E of Puquio at km 172 mark, 14°41'18.5"S, 74°4'26.8"W, 3730 m, 8 Apr 2004, P.M. Peterson & N. Refulio Rodriguez 18214 (MO, US, USM); Parinacochas, along arroyo near small rio above (north) of Laguna Parinacochas, 15°14'41"S, 73°44'20.3"W, 3300 m, 1 Mar 2002, P.M. Peterson, M. La Torre, A. Ramírez & D. Susanibar Cruz 16326 (US, USM). **Cajamarca**: Cajamarca, 21 km W of Central Plaza of Cajamarca at Cumbemayo (take Avenida Peru), 3600 m, 31 Mar 1997, P.M. Peterson & N. Refulio Rodriguez 14014 (MO, US, USM); Cumbe Mayo, subiendo el curso del canal, 3500 m, 20 Mar 1988, I. Sánchez Vega 4692 (CPUN); Cumbe Mayo, west of Cajamarca. growing in seeping water, 3600 m, 27 Mar 1988, S.A. Renvoize, S. Lægaard & I. Sánchez Vega 4997 (CPUN, K).Cordillera Comullca, 57 km. from Cajamarca on road to Celendín,“Jalca” páramo, 3600 m, 26 Mar 1988, S.A. Renvoize & S. Lægaard 4964 (CPUN, K); Pampa de Guanico, ruta a Guagal, 17 May 1973, 3500 m, I. Sánchez Vega.1145 & J. Tejada Mayta (CPUN); desvío a Guagal, ruta a Celendín, 2700 m, 17 May 1977, I. Sánchez Vega 2027, W. Ruiz V. & J. Sánchez Vega.(CPUN); Dist. La Encañada. Cerro Negro, extremo SE de las pampas de Cerro Negro, 3480 m, 19 Jul 2003, M. Cabanillas Soriano 1548, M. Cabanillas Medina & M. Sánchez Montoya (CPUN); entre Cajamarca y La Encañada, 2700 m, 22 May 1974, I. Sánchez Vega 1302 & W. Ruiz Vigo (CPUN); Dist. La Encañada, Pampas de Cerro Negro, 3540 m, 12 Jul 2003, M. Cabanillas Soriano 1501, M. Cabanillas Medina & M. Sánchez Montoya (CPUN); Cajamarca to Bambamarca, on side road to Chugur, 88 km from Cajamarca, just below first mine, 3700 m, 20 Mar 1988, S.A. Renvoize, S. Lægaard & I. Sánchez Vega 4822 (CPUN, K); Las lagunas, 50 km from Cajamarca on road to Bambamarca, 4000 m, 29 Mar 1988, S.A. Renvoize & S. Lægaard 5030 (CPUN, K); Dist Cajamarca. Pampa Larga, al N de Yancocha, 3900 m, 14 May 1994, I. Sánchez Vega 7135, Homero Bazán & Alfonso Miranda (CPUN); Micuypampa, 62 km from Cajamarca towards Celendin, 2600 m, 26 Mar 1988, S.A. Renvoize & S. Lægaard 4963 (CPUN, K);Celendin, S of Cajamarca−Celendin Hwy, E of pass between provincia borders and ca. 11 km along road to Huagal, vic. La Honda, 7°5'15.6"S, 78°11'39.9"W, 3525 m, 28 Mar 2008, P.M. Peterson, R.J. Soreng & J. Montoya Quino 21912 (US, USM); San Miguel, 61 km N of Cajamarca on Hwy 3N towards Bambamarca, 3640 m, 16 Mar 2000, P.M. Peterson & N. Refulio Rodriguez 14915 (CPUN, MO, US, USM); San Pablo, 33 km NW of Cajamarca on road towards San Pablo via Porcon, 3510 m, 14 Mar 2000, P.M. Peterson & N. Refulio Rodriguez 14876 (CPUN, MO, US, USM). **Cusco**: Calca, 26 km E of Pisac on road to Colquepata, 13°23'2.1"S, 71°44'46.9"W, 4120 m, 18 Mar 2002, P.M. Peterson & N. Refulio Rodriguez 16602 (US, USM); Canchis, Dist. Marangani, Lay Raya, 14.46666S 71.016666W, 7 May 2003, L. Valenzuela, J. Farfán, I. Huamantupa, E. Suclli, & H. Coasaca 1970 (MO); Cusco, Sacsaihuamán, 3600 m, Apr 1944, C. Vargas C. 4167 (US); Espinar, alrededores de Yauri, 3900 m, 26 Mar 1956, C. Vargas C. 11213 (US); Espinar, Hda. C’achachi, 4200 m, 20 Mar 1956, C. Vargas C. 11160 (US); Paucartambo, 21 km NE of Paucartambo on road to Tres Cruces., 13°11'54.8"S, 71°38'40.5"W, 3460 m, 18 Mar 2002, P.M. Peterson & N. Refulio Rodriguez 16621 (US, USM); 1 km E of Mirador Tres Cruces and 35 km N of Paucartambo, 13°8'11.6"S, 71°37'40.3"W, 3625 m, 19 Mar 2002, P.M. Peterson & N. Refulio Rodriguez 16639 (US, USM); Quispicanchi, 23 km up new Hwy 28 from Urcos to Ccatcca (Catsa), ESE of Cusco ca. 45 air km, 13°39'39.5"S, 71°35'29.7"W, 4080 m, 19 Mar 2007, P.M. Peterson, R.J. Soreng & K. Romashchenko 20564 (US, USM); Vilacabamba, Paltaybamba, Ayangate, 13.0494444S 72.7208334W, 2300 m, 10 Jun 2002, L. Valenzuela, E. Suclli, I. Huamantupa & F. Carazas 225 (MO). **Huancavelica**: Castrovirreyna, 30 km SW of Huancavelica along Rio Pucapampa, 12°56'10"S, 75°5'57.2"W, 4510 m, 8 Mar 2002, P.M. Peterson & O. Tovar 16399 (US, USM); Castrovirreyna, Choclococha, 4500 m, 5 May 1958, O. Tovar 2907 (US, USM); Castrovirreyna, Choclococha, 4700 m, 3 May 1958, O. Tovar 2854 (US, USM); 3 km S of Astobamba on road towards Pucapampa, 12°58'47.7"S, 75°5'27.4"W, 4540 m, 4 Apr 2004, P.M. Peterson & N. Refulio Rodriguez 18118 (MO, US, USM); 4 km S of Choclococha and 8 km N of Santa Ines, 13°10'44"S, 75°5'15.3"W, 4615 m, 5 Apr 2004, P.M. Peterson & N. Refulio Rodriguez 18145 (MO, US, USM); Huancavelica, 56 km S of Izacuchaca on road towards Huancavelica, 4020 m, 10 Apr 1997, P.M. Peterson & O. Tovar 14157 (US, USM);15 km NE of Huancavelica above Sachapite, 4100 m, 10 Apr 1997, P.M. Peterson & O. Tovar 14171 (US, USM); 12 km S of Huancavelica on road towards Santa Barbara, 4120 m, 11 Apr 1997, P.M. Peterson & O. Tovar 14191 (MO, US, USM); 13 km N of Huancavelica on road towards Palca, 12°43'9"S, 74°59'11.9"W, 4470 m, 2 Apr 2004, P.M. Peterson & N. Refulio Rodriguez 18098 (MO, US, USM); Huaytara, Rio Pampas, along Hwy 3 (old 24) 12 km NW of jct. with new Hwy 24 and 18 km SE of Santa Ines, SE of Pilpichaca, 13°19'24.2"S, 75°00'56.6"W, 4164 m, 13 Mar 2007, P.M. Peterson, R.J. Soreng, K. Romashchenko & D. Susanibar Cruz 20432 (US, USM); Occoro, entre Conaica y Tansiri, 4200 m, 2 Apr 1953, O. Tovar 1195 (US, USM); Occoro, 4200 m, 3 Apr 1953, O. Tovar 1214 (US, USM); Tayacaja, Hda. Alalay entre Mariscal Caceres y Pampas, 3900 m, 13 Apr 1953, O. Tovar 1333 (MO, US, USM); Tayacaja, 2 km E of Acostambo on road towards Izcuchaca, 12°22'3.7"S, 75°2'33.3"W, 3350 m, 1 Apr 2004, P.M. Peterson & N. Refulio Rodriguez 18087 (US, USM); Tayacaja, Patacancha, Conaica, 4100 m, 8 Apr 1961, O. Tovar 3131 (US, USM); Chascca, cerca a Conaica, 3800 m, 13 May 1956, O. Tovar 2588 (US, USM); Tayacaja, arriba de Hda. Toca, entre Calcabamba y Paucarbamba, 3800 m, 20 Apr 1954, O. Tovar 2021 (US, USM); Urubamba, Piri, 2860 m, 15 May 1948, C. Vargas C. 7208 (US). **Junin**: Junin, SE shore of Lago Chinchay Cocha (Junin) and 8 km S of Huayre, 4110 m, 7 Apr 1997, P.M. Peterson & O. Tovar 14097 (MO, US, USM); Tarma, Hacienda Casa Blanca, 18 air km SSE of Tarma, 4000 m, 27 Nov 1962, H.H. Iltis, C.M. Iltis, D. Ugent & V. Ugent s.n. (US); Yanli, La Oroya, 11°32'S, 75°53'W, 3700 m, 7 Jan 1983, D.N. Smith 2974 (MO, US); Yanli, Marcapomacocha, 4800 m, O. Velarde 8751 (US, USM). **La Libertad**: Bolivar, E side of Cerro Salumpuy, between Laplat and Unamen, WSW of Unamen, ca. 8 air km NW of Bolivar, 7°7'39.2"S, 77°46'9.9"W, 3390 m, 30 Mar 2008, P.M. Peterson, R.J. Soreng & J. Montoya Quino 21946 (US, USM); Pataz, cueva de Manachaqui, Parque Nacional Río Abiceo, 3600 m, 28 Feb 1998, B. León & K. Young 1116. (MO, USM); Santiago de Chuco, 23 km SW of Huamachuco on road towards Alto de Tamboras and Pampas, 3540 m, 29 Mar 1997, P.M. Peterson & N. Refulio Refulio 13966 (MO, US, USM). **Lambayeque**: Ferrenafe, 3300 m, 12 Sep 1985, A. Sagástegui A., D. Skillman, J. Mostacero L. & L. Ramírez V. 12812 (MO); Laguna Tembladera, Uyurpampa, 3320 m, 25 Aug 1993, S. Llatas Quiroz 3359 (MO). **Pasco**: Pasco, 4 km NE of Huayllay on road towards Canchacucho, 10°58'20.8"S, 76°19'18"W, 4160 m, 31 Mar 2004, P.M. Peterson & N. Refulio Rodriguez 18067 (MO, US, USM); plains ca. 8 km W of NW arm of Laguna de Junin, S side of Rio Colorado and E of new Hwy and railroad tracks, 12 km NE of Huayllay on Hwy 18, 10°55'51.9"S, 76°18'8.6"W, 4141 m, 5 Mar 2007, P.M. Peterson, R.J. Soreng & K. Romashchenko 20314 (US, USM). **Piura**: Ayabaca, Ruinas de Aypate, 2700 m, 21 May 1996, V. Quipuzcoa S. 544, O. Angulo Z. & R. Yahuana R. (HAO, MO); Huancabamba, 23 km E of Sondor on road towards Tabaconas, 2740 m, 31 Mar 2000, P.M. Peterson & N. Refulio Rodriguez 15133 (CPUN, MO, US, USM). **Puno**: Abra la Raya, along railroad between Santa Rosa and Marangani, 4300 m, 18 May 1985, J.C. Solomon 2920 (MO); Azangaro, Checayani, NE of Azangaro, 3900 m, 27 Mar 1957, H. Ellenberg 426 (US); Chuquibambilla, Florez 13 (WS); Granja Salcedo, Apr 1936, J. Sonkup 112 (US); Cerro Santa Barbara, 3700 m, 15 May 1958, O. Tovar 3071 (US, USM); Huerta, N of Puno, 3900 m, 22 Mar 1957, H. Ellenberg 242 (US); Pucará, Pampa, 3890 m, Apr 1964, A. Vera 105 (COL); alrededores de Puno, 3870 m, 16 Apr 1965, A. Vera 204 (COL); quebrada en los alrededores de Puno, 3850 m, 1 Oct 1965, A. Vera 340 (COL); Chucuito, 3 km NE of Zepita on road towards Copani, 3820 m, 5 Mar 1999, P.M. Peterson, N. Refulio Rodriguez & F. Salvador Perez 14633 (US, USM); 2 km S of Huacullani, 4000 m, 6 Mar 1999, P.M. Peterson, N. Refulio Rodriguez & F. Salvador Perez 14659 (MO, US, USM); Lampa, NW end of Laguna Lagunillas, 20 air km W of Santa Lucia, N and S sides of new Hwy 30 to Arequipa, 74 air km WSW of Juliaca, 15°41'1.7"S, 70°48'5.4"W, 4185 m, 31 Mar 2007, P.M. Peterson, R.J. Soreng, K. Romashchenko & M.S. Gonzalez Elizondo 20735 (US, USM); Melgar, ca. 7 km WNW of Santa Rosa on Hwy 3 and 1 km W toward Quishuara, along Rio Santa Rosa, 14°35'54.9"S, 70°51'34.7"W, 4002 m, 23 Mar 2007, P.M. Peterson, R.J. Soreng & K. Romashchenko 20606 (US, USM); Puno, 2 km N of Laraquerion road towards Puno, 16°6'59.1"S, 70°1'59.1"W, 3940 m, 18 Apr 2004, P.M. Peterson & N. Refulio Rodriguez 18328 (MO, US, USM); San Roman, 21 km W of Santa Lucia and 5 km E of Puente Cañuma (Laguna Lagunillas), 15°40'4.1"S, 70°47'4.4"W, 4260 m, 20 Apr 2004, P.M. Peterson & N. Refulio Rodriguez 18345 (MO, US, USM). **San Martín**: Mariscal Caceres, 7°00'01"S, 77°00'3"W, 3350 m, 18 Jul 1987, K. Young & B. León 4793 (MO, USM).

#### 12. Muhlenbergia maxima

Taxon classificationPlantaePoalesPoaceae

Lægaard & Sánchez Vega, Nordic J. Bot. 10: 439. 1990.

Fig. 2F−H

##### Type.

Peru, Prov. Cajamarca, Choten between Paso El Gavilán and San Juan at km 153 on the road to the coast, 2900 m, 29 May 1984, *I. Sánchez Vega & Ruiz Vigo 3561* (holotype: CPUN!; isotypes: AAU!, K!, US!, USM!).

##### Description.

Loosely caespitose *perennials*. *Culms* 100−140 cm tall, erect, rigid, terete, ca. 2−3.2 mm diameter near base with 3 or 4 glabrous to pubescent nodes; internodes terete above. *Leaf sheaths* longer than the internodes, mostly basally inserted, compressed-keeled near base, becoming fibrous with age, scaberulous, finely striate; *ligules* 3−5 mm long, apex irregularly toothed to lacinate, margins entire and extended above to form auricles; *blades* 25−45 cm long, 2.8−4 mm wide, flat to folded, striate, scabrous, apex attenuate, midvein prominent. *Panicles* 30−46 cm long, 3−6 cm wide, narrow; *branches* 6−11 cm long, scabrous, ascending, appressed to loosely spreading, mostly floriferous to base, except on lowest branches; *pedicels* mostly shorter than the spikelets. *Spikelets* 2.5−2.8 mm long, 1-flowered, greenish with reddish-purple tinting; *glumes* 2.2−2.6 mm long, almost equal or shorter than the floret, hyaline to membranous, faintly 1-veined, subequal, the lower usually slightly shorter, glabrous to scattered pubescent, scaberulous; *lemmas* 2.5−2.8 mm long, ovate, 3-veined, awned, with scattered sericeous hairs more numerous along the veins, scaberulous, the callus pilose, the flexuous awn inserted just below the obtuse to acute apex, the awn 4−9 mm long; *paleas* about as long as the lemma, faintly 2-veined, sericeous between the veins below; *lodicules* about 0.1 mm long, truncate, glabrous; *stamens* 3; anthers 1.3−1.8 mm long, purple. *Caryopses* 1.4−1.7 mm long, fusiform, brownish.

##### Distribution.

A Peruvian endemic known only from Amazonas and Cajamarca departments.

##### Ecology.

Rocky slopes near rivers and grassy slopes in disturbed ground along roads associated with *Acacia*, *Begonia*, *Cortaderiabifida*, *C.jubata*, *Desmodium*, *Dodonaeaviscosa* Jacq., *Hyptis*, *Lepechinia*, *Oxalis*, *Puya*, *Salvia* and *Schizachyrium*; 2100−2900 m.

##### Comments.

*Muhlenbergiamaxima* is a member of M.subg.Trichochloa (Peterson et al. 2010b). *Muhlenbergiainaequalis* Soderstr. endemic to Colombia and Venezuela and *M.lehmanniana* Henr. ranging from Costa Rica to Colombia (Lægaard and Sánchez Vega 1990; Giraldo-Cañas and Peterson 2009) are morphologically similar to *M.maxima*. *Muhlenbergiamaxima* differs from *M.inaequalis* and *M.lehmanniana* in having shorter lemmatal awns and a longer ligule than *M.inaequalis* (0.5−1.5 mm long) and a shorter ligule than *M.lehmanniana* (1−2.5 cm long). There is not much genetic variation among all members of M.subg.Trichochloa in Peterson et al. (2010a) and a detailed population genetic study of these three South American species is needed to interpret their evolutionary history.

##### Specimens examined.

Peru. **Amazonas**: 27 km from Balsas towards Leymabamba, 2250 m, 23 Mar 1988, S.A. Renvoize & S. Lægaard 4908 (CPUN, K); 66 km from Leymebamba towards Balsas, 2100 m, 25 Mar 1988, S.A. Renvoize & S. Lægaard 4935 (CPUN, K, MO, US). **Cajamarca**: Prov. Cajabamba, 14 air km WSW of Cajabamba and 2.5 km on road W of Araqueda, 7°39'9.1"S, 78°9'58.9"W, 2242 m, 23 Mar 2008, P.M. Peterson & R.J. Soreng 21853 (MO, US, USM); Prov. Cajamarca, S of Paso El Gavelán, ca. 10 air km S of Cajamarca, just above km post 150 on hwy 8 above San Juan, 7°15'37.2"S, 78°30'34.5"W, 2544 m, 26 Mar 2008, P.M. Peterson, R.J. Soreng & I Sánchez Vega 21884 (US, USM); Dist. San Juan, entre San Juan y Paso el Gavilán, 2650 m, 30 May 2003, I. Sánchez Vega 11965, M. Sánchez M. y R. Cueva R. (CPUN, HAO); Celendin, Marañón River Valley, Chachapoyas−Cajamarca road, 2100 m, 28 May 1984, D.N. Smith & J.M. Cabanillas S. 7271 (MO, US).

#### 13. Muhlenbergia microsperma

Taxon classificationPlantaePoalesPoaceae

(DC.) Kunth, Révis. Gramin. 1:64. 1829.

Fig. 7E−H

Trichochloa microsperma DC., Cat. Pl. Horti Monsp. 151. 1813. Muhlenbergiamicrosperma (DC.) Trin., Gram. Unifl. Sesquifl. 193. 1824, *nom. inval*. Type: Mexico, cultivated at the botanical gardern at Montpellier from seeds collected in Mexico and distributed by the Botanical Garden of Madrid, *M. Sésse & J.M. Mociño s.n.* (holotype: MPU; isotypes: G-00099434 [image!], P!, US fragm. ex P!).
Agrostis microsperma Lag., Gen. Sp. Pl. 2. 1816. Type: Mexico, plants grown at H.R. Matritensis (= Herbario del Real Jardín Botánico de Madrid, see Sutherland 1997) from seeds collected by M. Sessé & J.M. Mociño in Nueva Espania, Oct, 1806, *M. Sessé & J.M. Mociño s.n.* (lectotype, here designated: SEL-H10620 [image!]).
Podosemum debile Kunth, Nov. Gen. Sp. (quarto ed.) 1: 128. 1816. Trichochloadebilis (Kunth) Roem. & Schult., Syst. Veg. 2:385. 1817. Muhlenbergiadebilis (Kunth) Trin., Gram. Unifl. Sesquifl. 193, t. 5, f. 18. 1824. Type: Ecuador, Prov. Pichincha, Quito, *F.W.H.A. Humboldt & A.J.A. Bonpland s.n.* (holotype: P-Bonpl!; isotypes: B-W, P!, US-91924 fragm. ex P-Bonpl!).
Podosemum setosum Kunth, Nov. Gen. Sp. (quarto ed.) 1:129. 1816. Trichochloasetosa (Kunth) Roem. & Schult., Syst. Veg. 2:386. 1817. Agrostissetosa (Kunth) Spreng., Syst. Veg. 1:262. 1825. Muhlenbergiasetosa (Kunth) Trin., Gram. Unifl. Sesquifl. 193, t. 5, f. 22. 1824. Muhlenbergiasetosa (Kunth) Kunth, Révis. Gramin. 1:63. 1829, *isonym*. Type: Mexico, between Gueguetoque and Tula, Aug, *F.W.H.A. Humboldt & A.J.A. Bonpland 4174* (holotype: P-Bonpl!; isotypes: B-W, US-91917 fragm. ex P-Bonpl!).
Muhlenbergia purpurea Nutt., J. Acad. Nat. Sci. Philadelphia, ser. 2, 1:186. 1848. Type: U.S.A., California, Santa Barbara Co., Santa Barbara and Santa Catalina Island, *Gambel s.n.* (holotype: K!).
Muhlenbergia ramosissima Vasey, Bull. Torrey Bot. Club 13(12):231. 1886. Type: Mexico, Chihuahua, SW Chihuahua, Aug–Nov 1885, *E. Palmer 158* (lectotype: NY-00381467! designated by Hitchcock, N. Amer. Fl. 27:441. 1935, but without indicating the specific specimen; Peterson and Annable, Syst. Bot. Monogr. 31:61. 1991, indicated the specific specimen; isolectotypes: LE!, GH-00024043 [image!], MO-2974152!, NY-00381468 [image!], P-00644106!, PH-00018772 [image!], US-995580!, W-19160027660 [image!]).

##### Description.

Caespitose *annuals*, sometimes appearing as short-lived perennials. *Culms* 10–80 cm tall, often geniculate at the base, slender, often striate, much branched near the base, scaberulous below the nodes; *internodes* 1.8–8.6 mm long, mostly scaberulous or smooth. *Leaf sheaths* 2.2–6.6 mm long, commonly shorter than the internodes, glabrous, smooth or scaberulous; *ligules* 1–2 mm long, membranous to hyaline, decurrent, margins often extended, apex truncate to obtuse; *blades* 3–8.5(–10) cm long, 1–2.5 mm wide, flat or loosely involute, scabrous below, strigulose above, often deciduous with age. *Panicles* 6.5–13.5 cm long, 1–6.5 cm wide, open and not densely flowered, often purplish; *primary branches* 1.6–4 cm long, ascending or diverging up to 80° from the rachises, spikelet-bearing to the base; *pedicels* 2–6 mm long, appressed to divaricate, antrorsely scabrous. *Cleistogamouspanicles* with 1–3 spikelets present in the axils of the lower sheaths. *Spikelets* 2.5–5.5 mm long; *glumes* 0.4–1.3 mm long, exceeded by the florets, 1-nerved, obtuse, often minutely erose; *lower glumes* 0.4–1 mm long; *upper glumes* 0.6–1.3 mm long; *lemmas* 2.5–3.8(–5.3) mm long, narrowly lanceolate, mostly smooth, scaberulous distally, lower 1/2 of the margins and midveins, the hairs 0.2–0.5 mm long, the callus hairy, apices acuminate, often bidentate, awned, awns 10–30 mm long, straight to flexuous; *paleas* 2.2–4.8 mm long, narrowly lanceolate, acuminate; *anthers* 0.3–1.2 mm long, purplish. *Caryopses* 1.7–2.5 mm long, fusiform, reddish-brown. 2*n* = 20, 40, 60.

##### Distribution.

*Muhlenbergiamicrosperma* occurs in Hawaii, south-western U.S.A., Mexico, Guatemala, Colombia, Venezuela, Ecuador (including the Galapagos Islands), Peru and Bolivia (Peterson and Annable 1991).

##### Ecology.

Rocky slopes, rock outcrops, sandy drainages, cliffs and disturbed roadsides usually in desert scrub vegetation with *Acacia, Aristida adcensionis* L., *Baccharis*, *Bothriochloa*, Bombacaceae, Cactaceae, *Dodonaeaviscosa*, *Eragrostisnigricans*, *E.lugens* Nees, *Fucraea*, *Heliotropium*, *Heteropogoncontortus* (L.) P. Beauv. ex Roem. & Schult., *Lantana*, *Muhlenbergiarigida*, *Pitcairnia*, *Prosopis*, *Puya*, *Salvia*, *Schinusmolle* and *Schizachyrium*; 1150−3500 m.

##### Comments.

*Muhlenbergiamicrosperma* can sometimes be confused with *M.romaschenkoi* and differs from it by having cleistogamous panicles in the axils of the lower sheaths and shorter, obtuse glumes, 0.4–1.3 mm long (glumes acute to acuminate, 2–2.8 mm long in *M.romaschenkoi*).

In a molecular DNA sequence study, *M.microsperma* forms a strongly supported clade with two other annuals, *M.appressa* C.O. Godding and *M.brandegei* C.G. Reeder, all members of M.subg.Muhlenbergia (Fig. 1B; Peterson et al. 2010b). These three species produce cleistogamous spikelets in the axils of the lower culm branches, enclosed by a sheath (Peterson and Annable 1991). Cleistogamous spikelets appear to have evolved twice within *Muhlenbergia*, once in M.subg.Muhlenbergia within the *M.appressa−M.brandegei−M.microsperma* clade and once in M.sect.Pseudosporobolus in *M.cuspidata* (Torr. ex Hook.) Rydb. (Morden and Hatch 1984; Peterson et al. 2010b).

##### Specimens examined.

Peru. **Ancash**: at the Rio Yanamayo crossing, 57 km N of San Luis on road towards Pomabamba, 2540 m, 24 Mar 1997, P.M. Peterson & N. Refulio Rodriguez 13896 (US, USM); 68 km from Casma towards Huaraz, 1700 m, 4 Apr 1988, S.A. Renvoize & S. Lægaard 5047 (CPUN, K); Pallasca, divide between Rio Conchucos and Rio Plata, low end of road up to Pampas from Rio Conchucos, above Mollapata crossing of Rio Tablachaca, 8°12'9.1"S, 77°56'20.6"W, 2233 m, 21 Mar 2008, P.M. Peterson & R.J. Soreng 21833 (MO, US, USM). **Apurimac**: Aymaraes, 16 km NW of Chalhuanca, 14°10'16.4"S, 73°19'26.1"W, 2670 m, 14 Mar 2002, P.M. Peterson & N. Refulio Rodriguez 16511 (US, USM); Chincheros, canyon of Rio Pampas, between Ayacucho and Abancay on hwy 7, 7 km S of bridge over Rio Pampas, 13°28'42.1"S, 73°49'32"W, 2049 m, 17 Mar 2007, P.M. Peterson, R.J. Soreng, K. Romaschenko & D. Susanibar Cruz 20535 (MO, US, USM). **Ayacucho**: alrededores de Huanta, 2400 m, 30 Apr 1964, O. Tovar 4829 (US, USM). **Cajamarca**: Cajabamba, WSW of Cajabamba, 14 air km E of Araqueda, 7°39'9.1"S, 78°9'58.9"W, 2242 m, 23 Mar 2008, P.M. Peterson & R.J. Soreng 21855 (US, USM); Cajamarca, Cerro Huacaris, valle de Cajamarca, 1 Jun 1971, I. Sánchez Vega 726, W. Ruiz Vigo & J. Tejada (CPUN); Cajamarca, abajo de San Juan, siguiendo la carretera Cajamarca–Pacasmayo, 1900 m, 18 May 1986, I. Sánchez Vega 4072 (CPUN); Cajamarca, entre San Juan y Magdalena, 1800 m, 15 May 1971, I. Sánchez Vega 657 & W. Ruiz Vigo (CPUN); entre Chancay y Valle de Condebamba, 2600 m, 6 May 1972, I. Sánchez Vega 944, W. Ruiz Vigo & M. Malpica R. (CPUN); Dist. Ichocán, en el Arboretum Ichocán II de CICAFOR, entre la localidad de Chacay y Valle Condebamba, 2450 m, 29 Mar 1981, I. Sánchez Vega 2409, V. Torrel & E. Medina (CPUN); Celendin, Marañón River Valley, Chachapoyas−Cajamarca road, 1900 m, 28 May 1984, D.N. Smith & J. Cabanillas 7254 (MO, US); Chota, Dist. Cochabamba, a 1km sobre la carretera Cochabamba–Cutervo, 1720 m, 15 Jun 1980, I. Sánchez Vega 2288 (CPUN). **Cusco**: Anta, Sisal, Limatambo, 2300 m, 15 Mar 1963, C. Vargas C. 14327 (US, USM); Anta, Sisal−Cunyaee, 2100 m, 14 May 1965, C. Vargas C. 16316 (US, USM). **Huancavelica**: Salcabamba−Colcabamba, camino a Pampas, 2000 m, 22 Apr 1962, O. Tovar 3834 (US, USM); Tayacaja, alrededores de Mayoc, 2400 m, 9 Apr 1966, O. Tovar 5589 (US, USM). **Huánuco**: Huánuco, W side of canyon of Rio Huallaga, S of Ambo, 7 km on hwy 3 towards Cerro de Pasco, 10°11'49"S, 76°9'54"W, 2345 m, 6 Mar 2007, P.M. Peterson, R.J. Soreng & K. Romaschenko 20326 (US, USM); Huánuco, 2125 M, 25 Apr 1923, J.F. Macbride 3514 (F, US); 5 Apr 1923, J.F. Macbride 3217 (F, US). **La Libertad**: Bolivar, 4.6 air km NW of Longotea, SE of San Vicente on road from Balsas to Longotea, 7°0'38"S, 77°54'15.2"W, 2058 m, 31 Mar 2008, P.M. Peterson, R.J. Soreng & J. Montoya Quino 21970 (MO, US, USM); Otuzco, Quirrpe−Membrillar, 1150 m, 7 Dec 1952, A. López Miranda 927 (US); Santiago de Chuco, 22 km E of Huamachuco on road towards Sarin above Rio Chusgon, 2500 m, 29 Mar 1997, P.M. Peterson & N. Refulio Rodriguez 13973 (US, USM). **Lima**: Canta, 2 km SE of San Jose Canta, 11°30'2"S, 76°40'23.4"W, 2232 m, 27 Mar 2004, P.M. Peterson & N. Refulio Rodriguez 17985 (MO, US, USM); Huarochiri, Matucana, 2430 m, 12 Apr 1922, J.F. Macbride & Featherstone 392 (F, US); Huarochiri, km 70 carretera Lima−Oroya, 1800 m, 15 May 1963, R. Ferreyra 14892 (US, USM); km 68, cerca Surco, entre Chosica y Matucana, 1400 m, 27 Mar 1955, R. Ferreyra 10498 (US, USM); km 72 de la carretera Lima−Oroya, cerca de Surro, 1800 m, 14 May 1948, R. Ferreyra 3485 (MOL, US, USM); Prov. Lima, above Paya, E. Asplund 10825 (NY). **Piura**: Huancabamba, 10 km N of Sondor and 3 km S of Huancabamba, 1860 m, 1 Apr 2000, P.M. Peterson & N. Refulio Rodriguez 15167 (CPUN, MO, US, USM). **Tacna**: Zarata, Sitiqui, 3500 m, 7 Apr 1959, C. Vargas C. 12588 (US, USM).

#### 14. Muhlenbergia monandra

Taxon classificationPlantaePoalesPoaceae

Alegría & Rúgolo, Darwiniana 39(1–2): 20, 22, f. 1–3.

Fig. 10A−M

##### Type.

Peru, Depto. Lima, Prov. Canta, 5 km arriba de San José en camino a Huamantanga, frente a Apio, 2700 m, 21 May 1999, *A. Granda Paucar & J.J. Alegría Olivera 2230* (holotype: MOL; isotypes: BRIT-23827 [image!], SI! [SI-016006 image!], TEX, US-3376226! [US-00901642 image!], USM-000747 [image!]).

##### Description.

Tufted *annuals*. *Culms* 4−25 cm tall, 0.3−0.7 mm in diameter near base, erect to decumbent, branching below, glabrous, 2 or 3 glabrous nodes, scaberulous below the nodes; *internodes* 0.5−9 cm long. *Leaf sheaths* 0.5−4.7 cm long, usually shorter than the internodes, compressed, mostly glabrous, scaberulous near the summit, margins scarious; *ligules* 1−3 mm long, hyaline, decurrent, apex truncate to obtuse, lacinate to irregularly dentate; *blades* (0.5−)1−8 cm long, 0.7−3.6 mm wide, flat or loosely folded, finely pilose above and scabrous below, margins scabrous, apex mostly acuminate, rarely acute. *Panicles* (0.5−)1−7 cm long, 2−9 mm wide, tightly spiciform, often interrupted below, elliptic to oblong, terminal and axillary, often partially included in the sheath below; *primary branches* 4−15 mm long, tightly ascending-appressed, verticillate below with 5−10 per node, scabrous; *pedicles* 0.1−1.2 mm long, shorter than the spikelets, scabrous. *Spikelets* 3.4−4.6 mm long, 1-flowered, tightly carinate, cleistogamous; *glumes* 3.5−4.7 mm long, longer than the floret, nearly equal in length, membranous, linear lanceolate, 1-veined, scaberulous along the midvein, apex acuminate, mucronate, the mucro 0.2−0.7 mm long; *lemmas* 1.5−2.1 mm long, hyaline to membranous, ovate, 3-veined, the lateral veins faint, mottled with irregular plumbeous areas, sparingly appressed pilose, the hairs 0.2−0.4 mm long, apex truncate to obtuse, subapically awned, the awns 1.2−5 mm long, straight; *callus* pilose; *paleas* 1.4−2 mm long, hyaline, ovate, sparingly pilose, apex truncate, the veins extending as mucros up to 0.2 mm long; *lodicules* 0.1−0.2 mm long, truncate; *stamen* 1, anthers 0.3−0.6 mm long, whitish to yellow. *Caryopses* 1−1.6 mm long, ovoid, laterally flattened, light brown.

**Figure 10.**
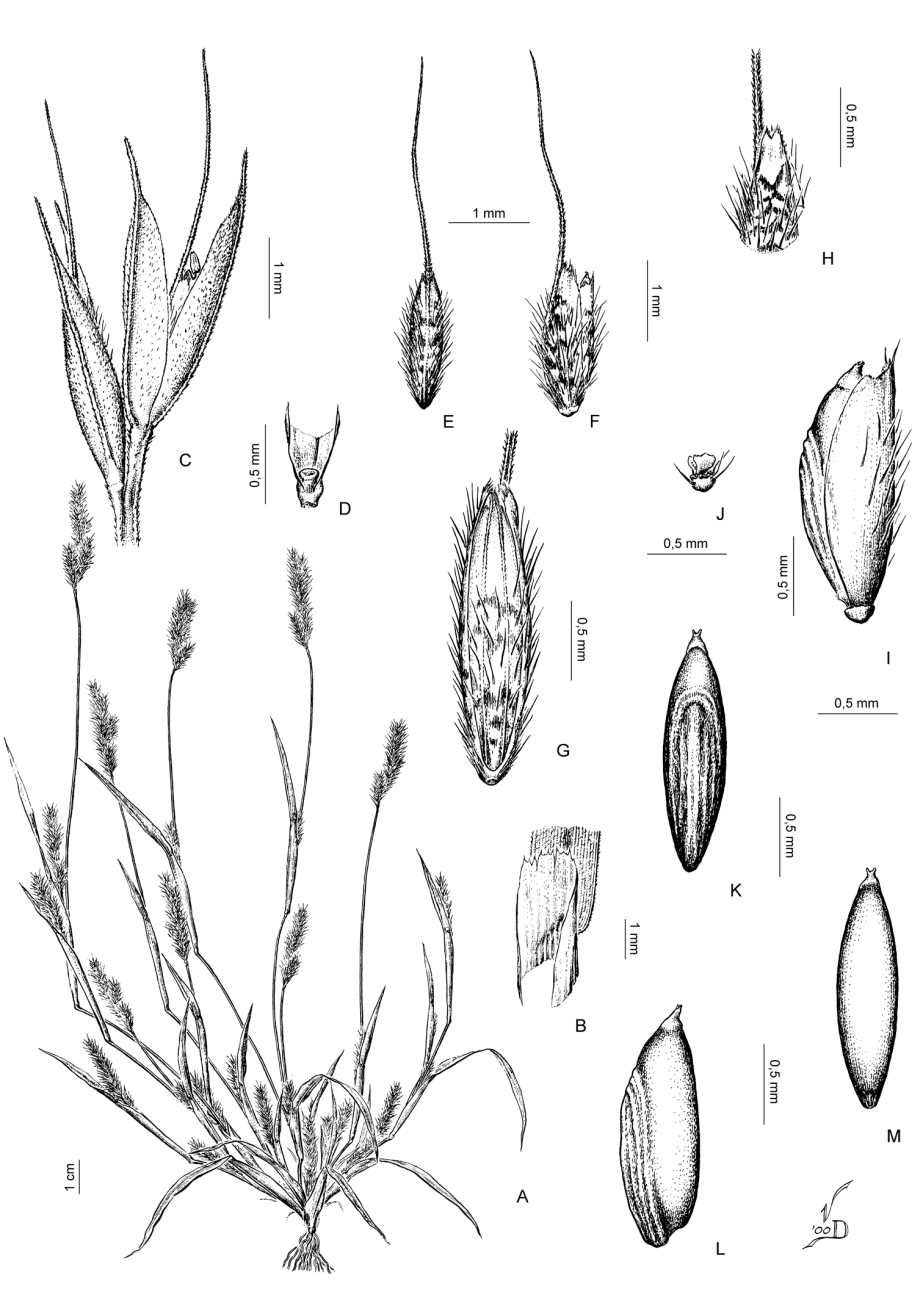
**A−M***Muhlenbergiamonandra* Alegría & Rúgolo **A** habit **B** ligule **C** two spikelets **D** glume base and rachilla **E** lemma, dorsal view **F** floret, side view **G** floret, ventral view **H** apex of lemma with two small teeth **I** palea and caryopsis, lateral view **J** lodícules **K** caryopsis, dorsal view **L** caryopsis, lateral view **M** caryopsis, ventral view. Drawings from the holotype collection *Granda Paucar & Alegría Olivera 2230*.

##### Distribution.

A Peruvian endemic known only from near San Jose de Canta, Peru.

##### Ecology.

This species occurs on gravelly slopes among xerophytic scrub vegetation with *Boutelouasimplex*, Eragrostismexicanasubsp.virescens (J. Presl) S.D. Koch & Sánchez Vega, *Muhlenbergiabryophilus*, *Urochloa* sp., *Veronica* sp. and *Festucamyruos* between 2700 and 2800 m.

##### Comments.

Molecular DNA sequence analysis indicates *Muhlenbergiamonandra* is an unsupported sister to the *M.atacamensis*-*M.ligulata*-*M.subbiflora* clade in M.subg.Pseudosporobolus (Fig. 1B).

##### Specimens examined.

Peru. **Lima**: Prov. Canta, 8 km SW of San Jose Canta towards Huamantango, 11°31'10.0"S, 76°42'16.3"W, 2770 m, 28 Mar 2004, P.M. Peterson & N. Refulio Rodriguez 17990 (MO, US, USM); entre Huamantanga y Puruchuco, 2800 m, 1 May 1994, J.J. Alegría Olivera 739 (MOL, SI).

#### 15. Muhlenbergia peruviana

Taxon classificationPlantaePoalesPoaceae

(P. Beauv.) Steud., Nomencl. Bot. (ed. 2) 1:41. 1840.

Fig. 11A−D

Clomena peruviana P. Beauv., Ess. Agrostogr. 28, t. 7, f. 10; t. 3, f. 20. 1812. Agrostisperuviana (P. Beauv.) Spreng., Syst. Veg. 1:262. 1825. Type: Peru, *M. Thibaut s.n.* (holotype: P!; isotype: E-00373717 [image!]).
Clomena peruviana var. pulvinata Nees, Gramineae 12–13. 1841. Muhlenbergiaperuvianavar.pulvinata (Nees) Nees & E. Mey. ex Kuntze, Revis. Gen. Pl. 3(3):357. 1898. Type: Peru, Lago Titicaca, Apr, *J.F.J. Meyen s.n.* (holotype: B; isotype: US-3376134 fragm. ex B!).
Muhlenbergia nana Benth., Pl. Hartw. 262. 1846. Type: Ecuador, Mt. Cotopaxi, 1843, *Hartweg 1458* (holotype: K!; isotypes: BAA-1629!, K!, LE!, P!, US-91916 fragm. ex P!, US-995896 fragm. ex P-STEUD & fragm. ex BR!).
Muhlenbergia pusilla Steud., Syn. Pl. Glumac. 1:177. 1854. Type: Mexico, México, Valley of Toluca, Oct. 1827, J.*L. Berlandier 1141* (holotype: P!; isotypes: BAA-1635!, K!, MO-2974185!, P!, US-1084517!, US-2561239!, US-91910 fragm. ex P!).
Epicampes bourgeaei E. Fourn., Mexic. Pl. 2: 88. 1886. Type: Mexico, Veracruz, Escamala, Refrou D’Orizaba, 26 Aug 1866, *E. Bourgeau 2973* (holotype: P!; isotype: US-A0865984 fragm ex P!).
Muhlenbergia bourgeaei E. Fourn., Mexic. Pl. 2:86. 1886. Epicampesbourgeaei (E. Fourn.) M.E. Jones, Contr. W. Bot. 14:7. 1912, *nom. illeg. hom.*Type: Mexico, Valle de Mexico, Desierto Viejo, 3 Nov 1865, *M. Bourgeau 1309* (lectotype: P! designated by Peterson and Annable, Syst. Bot. Monogr. 31:73. 1991; isotype: US-87243 fragm. ex P!).
Muhlenbergia pulcherrima Scribn. ex Beal, Grass. N. Amer. 2:240. 1896. Type: Mexico, Chihuahua: Sierra Madres, dry ledges of porphyry, 30 Sep 1887, *C.G. Pringle 1416* (holotype: MSC!; isotypes MO-3727978!, NY!, US-995494!, VT!).
Muhlenbergia peruviana var. elatior Kuntze, Revis. Gen. Pl. 3(2): 357. 1898. Type: Bolivia, Tunarigebirge, 3000 m, May 1892, *Kuntze s.n.* (lectotype: NY! designated by Peterson and Annable, Syst. Bot. Monogr. 31:73. 1991: isotype: fragm. & photo US!).
Muhlenbergia peruviana var. subcaespitosa Kuntze, Revis. Gen. Pl. 3(3):357. 1898. Type: Bolivia, Tunari Mts., 4600 m, 4 May 1892, *Kuntze s.n.* (lectotype: NY! designated by Peterson and Annable, Syst. Bot. Monogr. 31:73. 1991).
Muhlenbergia peruviana fo. versicolor Kuntze, Revis. Gen. Pl. 3(3):357. 1898. Type: Bolivia, Tunarigebirge, 3000 m, May 1892, *Kuntze s.n.* (lectotype: NY! designated by Peterson and Annable, Syst. Bot. Monogr. 31:73. 1991; isotype: US fragm. ex NY!).
Muhlenbergia peruviana fo. viridis Kuntze, Revis. Gen. Pl. 3(3):357. 1898. Type: Bolivia, Puna, 4000 m, 11 Mar 1892, *Kuntze s.n.* (lectotype: NY! designated by Peterson and Annable, Syst. Bot. Monogr. 31:73. 1991).
Muhlenbergia herzogiana Henrard, Meded. Rijks-Herb. 40:58. 1921. Type: Bolivia, Cordillera de Santa Bonita, Jun 1911, *T. Herzog 2226* (holotype: L!; isotypes: US-87248 fragm. ex L!, US-1161342!, W-1926-23724!).

##### Description.

Tufted *annuals*. *Culms* 3–27 cm tall, erect, glabrous. *Leaf sheaths* usually longer than the internodes, smooth or scabridulous; *ligules* 1.5–3 mm long, membranous, acute; *blades* 1–5 cm long, 0.6–1.5 mm wide, flat to involute, smooth or scabridulous abaxially, sometimes shortly pubescent adaxially. *Panicles* 2–8 cm long, 0.3–3.4 cm wide, contracted or open; *primary* branches 1–5 cm long, diverging up to 80° from the rachises; *pedicels* 0.4–5 mm long, smooth or scabrous. *Spikelets* 1.4 4.2 mm long, 1-flowered; *glumes* smooth or scabridulous; *lower glumes* 0.8–2.8 mm long, narrow to broadly lanceolate, 1-veined, acute, often awn-tipped; *upper glumes* 0.9–3 mm long, wider than the lower glumes, lanceolate, 3 (2)-veined, truncate to acute, 2- or 3-toothed; *lemmas* 1.4–4.2 mm long, ovate, widest near the base, purplish mottled with dark green areas, hairy on the calluses and lower 2/3 of the lemma bodies, hairs to 0.5 mm long, apices acuminate, usually bifid and awned from between the teeth, teeth to 0.5 mm long, awns 3–10 mm long, flexuous, purplish; *paleas* 1.3–3.8 mm, narrowly lanceolate, acuminate to subacute; *anthers* 0.5–1 mm long, purplish to yellowish. *Caryopses* 1–1.6 mm long, fusiform, brownish. 2*n* = 30.

**Figure 11.**
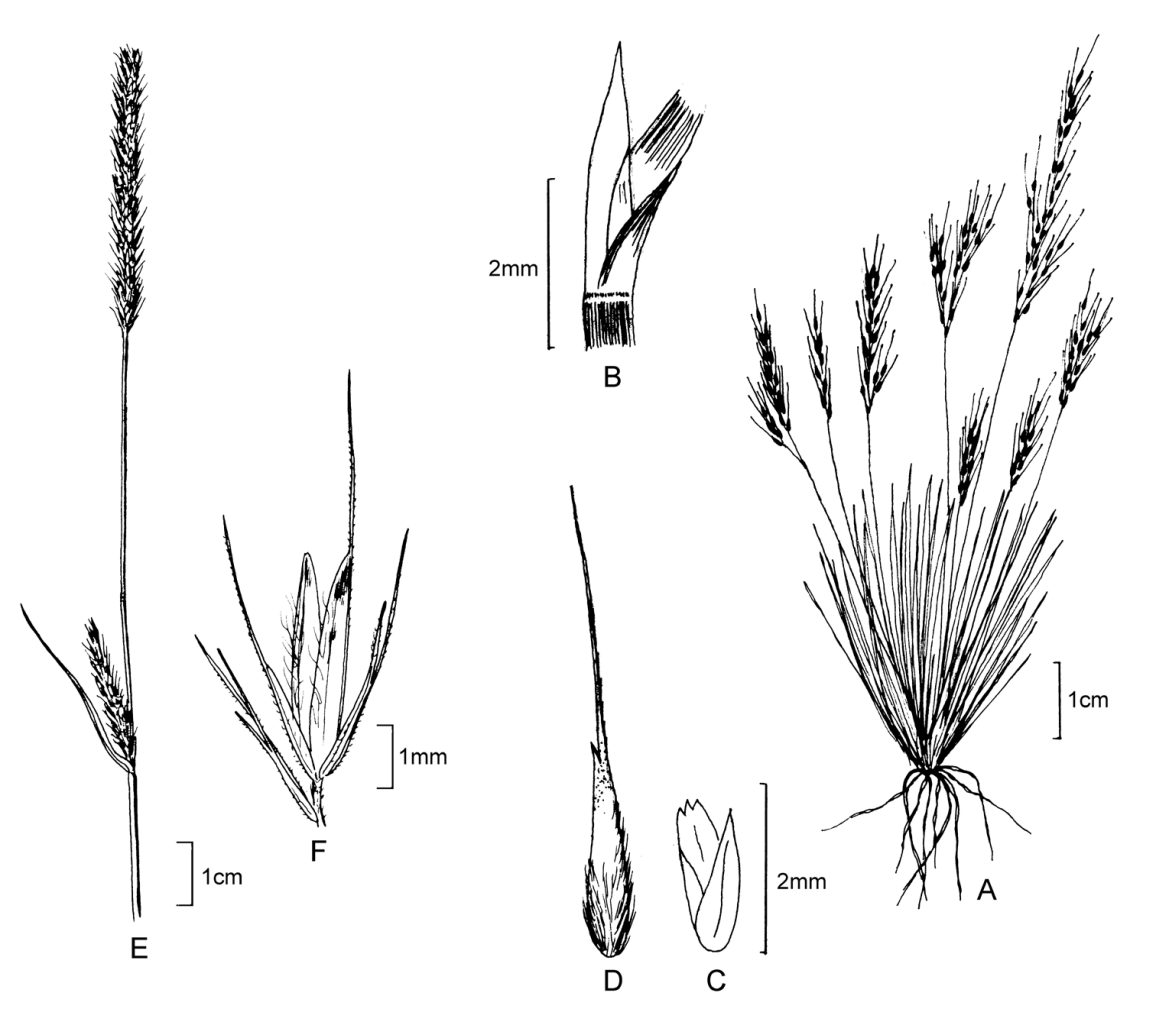
**A−D***Muhlenbergiaperuviana* (P. Beauv.) Steud **A** habit **B** ligule **C** glumes **D** floret **E, F***Muhlenbergiaphalaroides* (Kunth) P.M. Peterson **E** panicle **F** spikelet. Drawings **A−D** from Peterson and Annable (1991) drawn from *P.M. Peterson & C.R. Annable 4739* (WS) **E, F** from Giraldo-Cañas and Peterson (2009) drawn from *S. Lægaard 71419* (AAU).

##### Distribution.

*Muhlenbergiaperuviana* occurs in Arizona and New Mexico, U.S.A, throughout Mexico to Guatemala and then in Argentina, Bolivia, Chile, Ecuador and Peru (Peterson and Annable 1991).

##### Ecology.

Grassy flats, open gravelly flats, rock outcrops, sandy washes, gravelly drainages, wet or dry meadows, canyons, gravelly or sandy slopes, valleys, shores along lakes, open ridgetops and disturbed road cuts associated with *Aciachnepulvinata* Benth., *Anatherostipa*, *Berberis*, *Calamagrostis* spp., *Colletiaspinosissima*, *Ephedra*, *Festucaorthophylla* Pilg., *Festuca* spp., *Jarava*, *Juncus*, *Lepidophyllum*, *Luzula*, *Margyricarpus*, *Monnina*, *Muhlenbergia* spp., *Nassella*, *Plantago*, *Poa* spp., *Polylepis*, *Puya*, *Pycnophyllum*, *Salviaoppositiflora* Ruiz & Pav., *Stevia* and *Tagetes*; 3000−4900 m.

##### Comments.

As treated here, *Muhlenbergiaperuviana* includes (as synonyms) what was sometimes identified as *M.pulcherrima* Scribn. ex Beal (south-western U.S.A. and northern Mexico) and *M.pusilla* Steud. (central Mexico to Guatemala). There are many more morphological forms than just these and since the only chromosome count of this species suggests triploidy (2*n* = 3× =30), perhaps this species is apomictic (Reeder 1968). We believe apomixis is occurring in this species but it it not obligate and that gene flow takes place sporadically to form intermediates maintained by asexual seed formation (Peterson and Annable 1991).

In a molecular DNA sequence analysis, *Muhlenbergiaperuviana* is sister to *M.crispiseta* Hitchc., another annual known only from Texas and north-central Mexico and this pair is embedded in the strongly supported M.subg.Clomena clade (Peterson et al. 2010b; Fig. 1B). Members of M.subg.Clomena possess spikelets with upper glumes that are 3-veined and often 3-toothed and individuals with a densely caespitose habit (Peterson et al. 2010b). *Muhlenbergiaperuviana* can be separated from *M.crispiseta* in having irregularly flexuous, purplish awns (versus sinuous-wavy, crisped and curled, olive-green awns in *M.crispiseta*) and narrow, gradually acuminate lemmas (versus lemmas that are plump near middle) [Peterson and Annable 1991]. In our current DNA-derived phylogeny, *M.crispiseta* falls within the *M.peruviana* clade (Fig. 1B). However, *rps3*, the single marker we have for *M.crispiseta*, is not variable enough to distinguish these two species that are easily separated morphologically. In the future, we hope to clarify this with a full set of markers for *M.crispiseta*.

##### Specimens examined.

Peru. **Amazonas**: Prov. Chachapoyas, Cerros Calla Calla, 3100 m, 19 Jun 1964, D.C. Hutchison & J.K. Wright 5756 (LIL, MO). **Ancash**: Cordillera Blanca, 4 km north of Recuay, 3400 m, 6 Apr 1988, S.A. Renvoize & S. Lægaard 5101 (CPUN, K); Cordillera Negra, 3 km N of Punta Callán, W of Huaraz, 4400 m, 7 Apr 1988, S.A. Renvoize & S. Lægaard 5171 (CPUN, K); Cordillera Negra, 30 km from Huaraz towards Casma, Punta Callán, 4200 m, 7 April 1988, S.A. Renvoize & S. Lægaard 5166 (CPUN, K); Pariarracra, Pampa de Lampas, 4160 m, 2 May 1952, E. Cerrate 1465 (MOL); Prov. Bolognesi, Capillapunta, 3350 m, 14 Apr 1949, R. Ferreyra 5716 (MOL, USM); Prov. Bolognesi, cerros al E de Chiquián, 3500 m, 10 May 1950, R. Ferreyra 7345 (US, USM); Prov. Bolognesi, Matarragra, cerro al NO de Chiquián, 3560 m, 8 Apr 1949, R. Ferreyra 5633 (US, USM); Prov. Bolognesi, Shincush, arriba de Chiquián, 4000 m, 19 Apr 1949, R. Ferreyra 5624 (US, USM); Carhuaz, Cordillera Negra, E slope, ca. 22 km SW of Shupluy, 09°17'19.7"S, 77°44'39.3"W, 3770 m, 13 Mar 2008, P.M. Peterson, R.J. Soreng, M.I. La Torre & J.V. Rojas Fox 21655 (US, USM); Recuay, Cordillera Blanca, 37 km E of Raquia on Route 02-014 on road towards Huarez, 3900 m, 20 Mar 1997, P.M. Peterson & N. Refulio Rodriguez 13818 (MO, US, USM); Recuay, Cordillera Negra W slope N of Quebrada Parin, 23 km NE of Cotaparaco on road to pass above Lago Ututo and Utcuyacu, 09°55'10.7"S, 77°32'02.2"W, 4216 m, 10 Mar 2008, P.M. Peterson, R.J. Soreng, M.I La Torre & J.V. Rojas Fox 21564 (US, USM); Recuay, Cordillera Negra, en cima de Caraz, 4300 m, A. Weberbauer 3098 (MOL); Huaraz, Huascarán National Park, quebrada Llaca, north side of valley, 9°27'S, 77°27'W, 4350 m, 25 May 1986, D.N. Smith 12428 (CPUN, MO); Recuay, quebrada Huanca, 9°55'S, 77°23'W, 3950 m, 2 Jul 1985, D.N. Smith & M. Buddensiek 10959 (CPUN, LPB, MO); Recuay, Huascarán National Park, mouth of quebrada Quenua Ragra, 10°2'S, 77°15'W, 4200 m, 10 May 1985, D.N. Smith, R. Valencia & A. Gonzales 10624 (CPUN, MO); Huari, Huascarán National Park, south side of quebrada Carhuazcancha, 9°28'S, 77°15'W, 3790 m, 6 May 1986, D.N. Smith, R. Valencia, A. Gonzales & M. Buddensiek 12294 (CPUN, MO); Recuay, 2 km SW of Conococha on Ruta 02-014, 10°8'18.2"S, 77°18'5.4"W, 4107 m, 21 Mar 2004, P.M. Peterson, N. Refulio Rodriguez, A. Cano, M.I. La Torre & I. Salinas 17903 (US, USM); Recuay, 3 km E of Conococha, 10°6'55.5"S, 77°16'20.2"W, 4050 m, 24 Mar 2004, P.M. Peterson & N. Refulio Rodriguez 17939 (MO, US, USM); Recuay. Huascarán National Park, Sector Querococha, above guardstation, 9°44'S, 77°20'W, 3875 m, 5 Jul 1985, D.N. Smith & M. Buddensiek 11045 (CPUN, LPB, MO); Yungay, Huascarán National Park, Llanganuco sector, Maria Josefa trail between Chinancocha and Pucuyacu, 9°5'S, 77°39'W, 3700–3850 m, 7 May 1985, D.N. Smith 10548 (CPUN, MO). **Apurimac**: Aymaraes, 24 km NE of Chalhuanca on road towards Yanaca, 14°15'26.4"S, 73°12'18.8"W, 4260 m, 13 Mar 2002, P.M. Peterson & N. Refulio Rodriguez 16506 (US, USM). **Arequipa**: Arequipa,10 km W of Patahuasi on road towards Arequipa, 16°4'37.3"S, 71°29'49.8"W, 4100 m, 11 Apr 2004, P.M. Peterson & N. Refulio Rodriguez 18243 (MO, US, USM); Arequipa, Jesus−Chiqüata, 2900 m, Jun 1977, C. Vargas C. s.n. (LIL); Arequipa, cerca a Chiquata, 3100 m, 31 Mar 1949, C. Vargas C. 8092 (CUZ, US); Caylloma, 10 km SE of Callalli on road towards Condoroma, 15°29'26"S, 71°23'5.9"W, 4340 m, 12 Apr 2004, P.M. Peterson & N. Refulio Rodriguez 18270 (MO, US, USM); Prov. Caylloma, Pampa Cañahuas. “Llapa pasto”, 4200 m, 30 May 2001, M. Rodríguez Diaz 1126 (USM). **Ayacucho**: Marcahrasi, arriba de Puquio, 3400 m, 24 Apr 1950, R. Ferreyra 7215 (MOL, US, 8USM); Huamanga, S of Ayacucho ca. 23 km on Hwy 3 toward Abancay, 13°16'21.5"S, 74°13'46.8"W, 3575 m, 15 Mar 2007, P.M. Peterson, R.J. Soreng, K. Romashchenko & D. Susanibar Cruz 20497 (US, USM); Huamanga, S of Ayacucho ca. 27 km on Hwy 3 toward Abancay, 13°17'9.8"S, 74°13'41.9"W, 3684 m, 16 Mar 2007, P.M. Peterson, R.J. Soreng, K. Romashchenko & D. Susanibar Cruz 20508 (US, USM); Huanca Sancos, 16 km NW of Putajasa on road towards Huanca Sancos, 14°1'42.5"S, 74°16'16.6"W, 3900 m, 25 Feb 2002, P.M. Peterson, A. Cano, M.I. La Torre, A. Ramirez & D. Susanibar Cruz 16254 (US, USM); Lucanas, 13 km E of Puquio at km 172 mark, 14°41'18.5"S, 74°4'26.8"W, 3730 m, 8 Apr 2004, P.M. Peterson & N. Refulio Rodriguez 18213 (MO, US, USM); Lucanas, 25 km NE of Villatambo on road towards Lucanas at Reserva “Pampa Galeras”, 14°40'46.2"S, 74°23'59.2"W, 3805 m, 8 Apr 2004, P.M. Peterson & N. Refulio Rodriguez 18181 (MO, US, USM); Pampalca, between Huanta and Rio Apurimac, 3200 m, 4 May 1929, E.P. Killip & A.C. Smith 22216 (US). **Cajamarca**: Cajamarca, S of Cajamarca ca. 9 air km, 7°14'8.1"S, 78°19'17.9"W, 3270 m, 27 Mar 2008, P.M. Peterson, R.J. Soreng, I. Sánchez Vega & J. Montoya Quino 21885 (US, USM); Cajamarca, Cerro Piedras Gachas, entre Yanacocha y Llaucán, 3900 m, 23 Apr 1994, I. Sánchez Vega 6997 & M. Cabanillas S. (CPUN); Cajamarca, entre Cajamarca y Cumbe Mayo, Fundo de la Universidad, 3450 m, 22 May 1971, I. Sánchez Vega 685 & M. Vilhena (CPUN); Cajamarca, entre Cajamarca y Cumbe Mayo, km 14, en el Arboretum Cumbe Mayo CICAFOR, 3400 m, 18 Apr 1981, I. Sánchez Vega 2466, V. Torrel & E. Medina (CPUN); Cajamarca, Dist. Cajamarca. Sais Atahualpa de Porcón en el Arboretum de CICAFOR, 3400 m, 16 May 1981, I. Sánchez Vega 2524, V. Torrel & E. Medina (CPUN); Cajamarca, Dist. Cajamarca, Cerro Gavilán, 3100 m, 26 Jun 1966, I. Sánchez Vega 238 (CPUN); Cajamarca, Dist. Cajamarca, Hacienda Porcón, a 35 km de Cajamarca, 3400 m, 31 May 1969, I. Sánchez Vega 401 (CPUN); Cajamarca, Dist. Cajamarca, Cerro Minas, Granja Porcón, 35 km al N de Cajamarca, 3900 m, 22 Jun 1994, I. Sánchez Vega 7293 & G. Bazan H. (CPUN); Cajamarca. Dist. Cajamarca, Tamiacocha, Cerro Negro, Gavilán, distancia 5 km en línea recta, queda a la vertiente occidental, 7°15'S, 78°28'W, 3558 m, 26 May 2001, I. Sánchez Vega 10618 (CPUN); Cajamarca, ladera que coverge a la Colpa, 2900 m, 22 Apr 1975, I. Sánchez Vega 1419, P. Brandelard, J. Sanabria & W. Ruiz Vigo (CPUN); Cajamarca, Corisorgona, al Norte de la ciudad de Cajamarca, carretera a Chamis, 3050 m, 25 Apr 1991, I. Sánchez Vega 5585 & A. Briones (CPUN); San Miguel, 61 km N of Cajamarca on hwy 3N towards Bambamarca, 3640 m, 16 Mar 2000, P.M. Peterson & N. Refulio Rodriguez 14913 (CPUN, MO, US, USM). **Cusco**: Prov. Calea, Uchumuca−Pisae, Apr 1994, F. Marin 386 (LIL); Calca, 20 km N of Calca on road towards Lares, 13°13'52.9"S, 71°54'26.5"W, 4060 m, 16 Mar 2002, P.M. Peterson & N. Refulio Rodriguez 16546 (US, USM); Prov. Canchis, temple of Viracocha near Tinta, 3500 m, 15 Apr 1913, O.F. Cook & G.B. Gilbert 211 (US); Prov. Cusco, Ruinas Sacsahramán, 3500 m, 6 May 1983, S.G. Beck 8366 (LPB, MO); Prov. Cusco, Tambomachay, 3700 m, Apr 1949, F. Marin 1420 (US, LIL); Prov. Cusco, Cusco–Huancar, 3500 m, 18 Jun 1982, Gutte and Miller 9424 (USM); Prov. Espinar, alrrededores de Youri, 3900 m, 26 Mar 1956, C. Vargas C. 11210, 11211 (CUZ, US); Prov. Espinar, Hacienda C’uyo, 4200 m, 24 Mar 1956, C. Vargas C. 11180 (CUZ, US); Prov. Espinar, Hacienda C’achachi, 4200 m, 20 Mar 1956, C. Vargas C. 11151, 11163 (CUZ, US); Prov. Paucartambo, 1936, J. Soukup s.n. (US); Prov. Paucartambo, Acjanacu, 3600 m, 6 Jul 1948, C. Vargas C. 7299 (CUZ, US); Prov. Paucartambo, Yanamayo, 9 Jul 1946, C. Vargas C. 609 (CUZ, US); Quispicanchi, 23 km up new hwy 28 from Urcos to Ccatcca (Catsa), ESE of Cusco ca. 45 air km, 13°39'39"S, 71°35'29.7"W, 4080 m, 19 Mar 2007, P.M. Peterson, R.J. Soreng & K. Romashchenko 20563 (MO, US, USM); Prov. Urubamba, quebrada Pumahuanca, 3200 m, 31 Dec 1962, H.H. Iltis & C.M. Iltis 1014 (LIL). **Huancavelica**: Castrovirreyna, 4 km S of Choclococha and 8 km N of Santa Ines, 13°10'44"S, 75°5'15.3"W, 4615 m, 5 Apr 2004, P.M. Peterson & N. Refulio Rodriguez 18147 (MO, US, USM); Huancavelica, Huancavelica, 4000 m, 8 Apr 1961, O. Tovar 3171 (MO, USM); Huancavelica, 56 km S of Izacuchaca on road towards Huancavelica, 4020 m, 10 Apr 1997, P.M. Peterson & O. Tovar 14160 (MO, US, USM); Huancavelica, 12 km S of Huancavelica on road towards Santa Barbara, 4120 m, 11 Apr 1997, P.M. Peterson & O. Tovar 14189 (MO, US, USM); Huancavelica, Sachahuajta, a 6 km de Conaica, 3600 m, 3 Apr 1952, O. Tovar 944 (US, USM); Huancavelica, Huando, 3600 m, 6 Apr 1953, O. Tovar 1277 (US, USM); Huancavelica, Canyon of Rio Ichu, SW of Huancavelica ca. 5 km on hwy 3 to Santa Ines and Ayacucho, 12°48'48.2"S, 75°3'24.5"W, 4894 m, 14 Mar 2007, P.M. Peterson, R.J. Soreng, K. Romashchenko & D. Susanibar Cruz 20464 (MO, US, USM); Huaytara, along Rio Pampas, along hwy 3 (old 24), 12 km NW of jct with new hwy 24 and 18 km SE of Santa Ines, SE of Pilpichaca, 13°19'24.2"S, 75°0'56.6"W, 4164 m, 13 Mar 2007, P.M. Peterson, R.J. Soreng, K. Romashchenko & D. Susanibar Cruz 20438 (US, USM); Huaytara, 7 km W of Pilpichaca on road towards Huancavelica. 13°18'46.5"S, 75°2'7"W, 4250 m, 5 Apr 2004, P.M. Peterson & N. Refulio Rodriguez 18157 (MO, US, USM); Tayacaja, just above Chuquitambo, 4 km SW of Pazos, 3975 m, 9 Apr 1997, P.M. Peterson & O. Tovar 14150 (MO, US, USM); Tayacaja, arriba de Hacienda Tocos, entre Colcabamba y Paucarbamba, 3700 m, 20 Apr 1954, O. Tovar 1961, 2013 (US, USM); Tayacaja, Hacienda Huari, 3700 m, 10 Aug 1949, O. Velarde Nuñez 2038 (US, USM). **Huanuco**: 18 mi SE of Huanuco, 31 May 1922, J.F. Macbride & W. Featherstone 2114 (F, US); **Junín**: Prov. Cerro, Goyllarisquisca, 4100 m, 22 Jun 1940, E. Asplund (US); Prov. Huancayo, Acopalca, 3800 m, 20 Jul 1943, J. Infantes Vera 412 (LIL); Prov. Concepción, Cani, 7 mi NE of Mito, 2800 m, 16 Apr 1923, J.F. Macbride 3401 (F, US); Prov. Huancayo, Laive, 3600 m, 15 Mar 1947, J. Infantes Vera 920 (LIL); Prov. Jaupa, 6 km a Concepción, quebrada de Iscos, 3300 m, Mar 1947, C. Ochoa 81 (LIL, MOL, USM); Prov. Yauli, La Oroya, 3950 m, 27 May 1922, J.F. Macbride & W. Featherstone 984 (F, US); Prov. Yauli, Yauli, 4450 m, 25 May 1922, J.F. Macbride & W. Featherstone 916 (F, US). **La Libertad**: Prov. Otuzco, Purrupumpa, 3585 m, 15 May 1991, S. Leiva, P. Leiva & E. Zavaleta 273 (HAO); Prov. Stgo. Chuco, Cerro La Botica, arriba de Cachicadán, 3050 m, 10 Jun 2001, A. Sagastegui, S. Leiva & M. Zapata 16685 (HAO). **Lambayeque**: Ferrenafe, 3300 m, 29 Jun 1996, S. Llatas Quiroz 4121 (MO). **Lima**: arriba de Sucro, 3000 m, 1 May 1948, R. Ferreyra 3445 (MOL, US, USM); Prov. Huarochiri, Matucana, 13 Apr 1922, J.F. Macbride & W. Featherstone 450 (F, US). **Moquegua**: Carumas, 3200 m, 21 Feb 1925, A. Weberbauer 7295 (US); 1 km S of Puentes Vizcachas, 32 km NE of Humajalso and 18 km S of Titiri, 16°37'14.8"S, 70°24'50.2"W, 4310 m, 18 Apr 2004, P.M. Peterson & N. Refulio Rodriguez 18317 (MO, US, USM). **Pasco**: Pasco, 4 km NE of Huayllay on road towards Canchacucho at “Bosque de Piedras”, 10°58'20.8"S, 76°19'18"W, 4160 m, 31 Mar 2004, P.M. Peterson & N. Refulio Rodriguez 18068 (MO, US, USM). **Piura**: Huancabamba, abajo del Tabo, 3 Jul 1961, C. Acleto 313 (USM). **Puno**: Araranca, 4100 m, 21 Apr 1925, F.W. Pennell 13466 (US); W of Llave, 3900 m, 21 Jul 1946, O.P. Pearson & A. Pearson 8 (US); Prov. Carabaya, Ockopuno, 4800 m, Apr 1949, C. Vargas C. 8142 (CUZ, MO, US); Cerro Orco Pata, 4000 m, 17 Jan 1964, R.A. Arevalo 27 (LIL); Prov. Carabaya, Fauchinta Allinccapac, 4600 m, 1 Apr 1948, C. Vargas C. 7157 (CUZ, US); Provincia Lampa, distrito Pucará, inmediaciones del pueblo de Chijnaya, en sustratos arenoso-rocosos en los bordes de la carretera, 4000 m, 14 Jul 2013, D. Giraldo-Cañas 5532 (COL); Pucará, lugar Chijnaya, 3990 m, Mar 1964, A. Vera 108 (COL); Hacienda Iriapata, estación Pucará, 3990 m, Oct 1963, A. Vera s.n. (COL); Chuquibambilla, 3900 m, 19 Apr 1925, F.W. Pennell 13375-1 (US); Chucuito, 2 km S of Huacullani, 4000 m, 6 Mar 1999, P.M. Peterson, N. Refulio Rodriguez & F. Salvador Perez 14660 (MO, US, USM); Chucuito, 5 km S of Huacullani on road towards Kelluyo, 4250 m, 6 Mar 1999, P.M. Peterson, N. Refulio Rodriguez & F. Salvador Perez 14679 (MO, US, USM); Chucuito, 27 km NW of San Jose Ancomarca, 4280 m, 8 Mar 1999, P.M. Peterson, N. Refulio Rodriguez & F. Salvador Perez 14702 (MO, US, USM); El Collao, 34 km E of El Cruce and 24 km W of Mazo Cruz, 4180 m, 2 Mar 1999, P.M. Peterson, N. Refulio Rodriguez & F. Salvador Perez 14595 (MO, US, USM); Lampa, NW end of Laguna Lagunillas, 20 air km W of Santa Lucia, 74 air km WSW of Juliaca, 15°41'1.7"S, 70°48'5.4"W, 4185 m, 31 Mar 2007, P.M. Peterson, R.J. Soreng, K. Romashchenko & M.S. Gonzalez Elizondo 20738 (US, USM); Prov. Lampa, Pucará, A. Weberbauer 415 (US); Melgar, ca. 7 km WNW of Santa Rosa on hwy 3 and 1 km W towards Quishuara, along Rio Santa Rosa, 14°35'54.9"S, 70°51'34.7"W, 4002 m, 23 Mar 2007, P.M. Peterson, R.J. Soreng & K. Romashchenko 20604 (MO, US, USM); Puno, 2 km N of Laraquerion on road towards Puno, 16°6'59.1"S, 70°1'59.1"W, 3940 m, 18 Apr 2004, P.M. Peterson & N. Refulio Rodriguez 18329 (MO, US, USM); San Roman, 4 km E of Santa Lucia, along Rio Cabanillas, 15°41'4.2"S, 70°34'8.8"W, 4030 m, 19 Apr 2004, P.M. Peterson & N. Refulio Rodriguez 18334 (MO, US, USM); San Roman, 21 km W of Santa Lucia and 5 km E of Puente Cañuma (Laguna Lagunillas), 15°40'4.1"S, 70°47'4.4"W, 4260 m, 20 Apr 2004, P.M. Peterson & N. Refulio Rodriguez 18341 (MO, US, USM).

#### 16. Muhlenbergia phalaroides

Taxon classificationPlantaePoalesPoaceae

(Kunth) P.M. Peterson, Caldasia 31(2): 294–296, f. 7 A–B. 2009.

Fig. 11E, F

Lycurus phalaroides Kunth, Nov. Gen. Sp. (quarto ed.) 1: 142. 1815 (1816). Type: Mexico, Michoacán, near Valladolid, Alberca de Palangeo and Patzcuaro, Sep, *F.W.H.A. Humboldt & A.J.A. Bonpland s.n.* (holotype: P-00669405 [image!]; isotypes: B-W-1630, BM!, BAA-1530!, US-91988 fragm. ex P-BONPL!, US-610837 fragm. ex LE-TRIN!).
Muhlenbergia lycuroides Vasey ex Beal, Grass. N. Amer. 2: 239. 1896. Type: Mexico, Jalisco, Guadalajara, Jul−Oct 1886, *E. Palmer 489* (holotype: MSC; isotypes: GH-00023916 [image!], LE, MEXU, MO-2972929!, NDG-07247 [image!], NY, P-00644181 [image!], P-00644182 [image!], S14-29628 [image!], US-822925!, US-81642!, YU-000898 [image!]).
Lycurus phleoides var. brevifolius Scribn. ex Beal, Grass. N. Amer. 2: 271. 1896. Type: Mexico, Jalisco, plains of Guadalajara, 23 Oct 1889, *C.G. Pringle 2470* (lectotype: MSC, designated by C. Reeder, Phytologia 57(4): 288. 1985; isolectotypes: BAA!, GH, MEXU, MO-2972926!, NY!, P-00644183 [image!], P-00644184 [image!], US-996049!, W-18900000580 [image!], W-19160029092 [image!]).

##### Description.

*Perennials*, intricately branched near base. *Culms* 10−30 cm tall, erect, mostly glabrous, usually decumbent and sprawling below, bent at the pubescent to short pilose nodes; *internodes* 0.4−10 (−15) cm long, pubescent to short pilose. *Leaf sheaths* much shorter than the internodes above, hyaline near the margins, pilose near summit; *ligules* 0.4−1 mm long, membranous, apex truncate to deltoid, often erose and lacerate; *blades* 0.5−6.5 cm long, 0.5−1.2 mm wide, shorter near the base of culms, flat, folded or loosely involute, lanate above and glabrous or with scattered, short appressed hairs below, margins whitish-thickened, apex navicular, occasionally with a short seta, seta usually less than 2 mm long. *Panicles* 1.5−6.5 cm long, 3−8 mm wide, spiciform and spike-like, densely flowered, often interrupted below with only a few spikelets, terminal or axillary; *rachis* lanate to hispid, the short hairs antrose or appressed; *branches* 1.5−7 mm long, very short, the spiklets usually in pairs, rarely 1 or 3 per terminal branch, when in pairs the lower short-pedicelled spikelet perfect, staminate or sterile and the upper longer-pedicelled spikelet usually perfect; *pedicels* 0.3−1.4 mm long; *disarticulation* usually at the base of the pedicel, each spikelet falling as a unit leaving a small cup-like tip. *Spikelets* 3−4 mm long, stramineous with plumbeous mottles, sometimes additionally with purplish mottles; *glumes* 1−2.1 mm long, shorter than the lemma, subequal, 1−3-veined; *lower glumes* commonly 2 or 3-veined, usually 2-awned, ocassionally 1 or 3 awned, the awns 1−3 mm long, equal or subequal, scabrous, recurved; *upper glumes* commonly 1-veined, usually 1-awned, the awns 1−2.5 mm long; *lemmas* 3−4 mm long, narrowly lanceolate, 3-veined, margins hirsute to lanate and occasionally the lower ½ sparsely hairy, the hairs 0.1−0.3 mm long, apex usually awned, occasionally unawned or mucronate, the awns 1−3 mm long; *paleas* 2.8−3.8 mm long, hairy between the veins, the veins occasionally extending as mucros; anthers 1.3−2 mm long, yellowish. *Caryopses* 1.7−2 mm long, fusiform, brownish.

##### Distribution.

*Muhlenbergiaphalaroides* ranges from Mexico to South America where it is found in Argentina, Bolivia, Columbia, Ecuador and Peru (Reeder 1985; Sánchez and Rúgolo de Agrasar 1986; Davidse and Pohl 1994).

##### Ecology.

This species occurs in open grasslands and savannahs on steep rocky slopes flats and along disturbed irrigation canals in deep clayish-loam to sandy soils associated with *Baccharis*, *Berberis*, *Cheilanthes*, *Condalia*, *Dodonaeaviscosa*, *Eragrostis*, *JaravaichuOpuntia*, *Muhlenbergiacenchroides*, *M.rigida*, *Nassella*, *Plantago*, *Puya* and *Sporobolusindicus* (L.) R. Br.; 2800−3500 m.

##### Comments.

*Muhlenbergiaphalaroides* is morphologically similar to *M.phleoides* (Kunth) Columbus, known in south-western U.S.A. and Mexico and *M.alopecuroides* found in south-western U.S.A., Mexico and disjunct in Argentina and Bolivia (Davidse and Pohl 1994, Renvoize 1998, Reeder 2003; Peterson and Giraldo-Cañas 2012). *Muhlenbergiaalopecuroides* differs from *M.phalaroides* in having leaf blades with terminal seta (3−)4−7(−12) mm long and ligules(2−)3−12 mm long whereas *M.phleoides* differs in having auriculate ligules 1−2 mm long (Reeder 1985, 2003). These morphological differences are perhaps better recognised at the subspecific level but there have been no population studies comparing these three species, other than Peterson and Morrone (1997) who investigated populations of only the amphitropical, *M.alocpecuroides* [as *Lycurussetosus*].

*Muhlenbergiaphalaroides* lies within M.subg.Pseudosporobolus, aligning with *M.alopecuroides* and *M.phleoides* (Peterson and Romaschenko, in prep.). Many members of this subgenus have narrow *panicles*, plumbeous spikelets with unawned, mucronate or short-awned lemmas (Peterson et al. 2010b).

##### Specimens examined.

Peru. **Ancash**: 15 Mar 1947, J. Infantes Vera 1170 (LIL). **Ayacucho**: Huanca Sancos, 27 km NW of Putajasa and 3 km S of Sacsamarca, 13°57'51.1"S, 74°18'41.5"W, 3650 m, 25 Feb 2002, P.M. Peterson 16277, A.A. Cano, M.I. LaTorre, A. Ramirez & D. Susanibar (US, USM). **Cusco**: Canchis, Hacienda Occobamba, 3450 m, 4 Apr 1950, C. Vargas C. 9410 (CUZ, US); Prov. Quispicanchis, Pikillacta, 3250 m, 28 Apr 1946, C. Vargas C. 6033 (CUZ, US). **Huancavelica**: near Huancayo, 3270 m, 1 Apr 1952, O. Tovar 344 (US, USM); Sacxhahuajta, a 6 km de Conaica, 3600 m, 3 Apr 1952, O. Tovar 939 (US, USM). **Junín**: Huancayo, 3317 m, Mar 1943, J. Soukup 1900 (COL, LIL, US); Prov. Huancayo, Agua de las Virgenes, 3270 m, 1 Apr 1951, O. Tovar 344 (MOL, USM); Tarma, between La Oroya and La Merced, 3000 m, 24 Oct 1923, A.S. Hitchcock 22169 (US).

#### 17. Muhlenbergia rigida

Taxon classificationPlantaePoalesPoaceae

(Kunth) Kunth, Révis. Gramin. 1: 63. 1829.

Fig. 6D−F

Podosemum rigidum Kunth, Nov. Gen. Sp. (quarto ed.) 1: 129. 1816. Trichochloarigida (Kunth) Roem. & Schult., Syst. Veg. 2: 386. 1817. Agrostisrigida (Kunth) Spreng., Syst. Veg. 1: 262. 1825. Type: Mexico, Guanajuato, near Guanajuato, Sep, *F.W.H.A. Humboldt & A.J.A. Bonpland s.n.* (holotype: P!; isotypes: BAA!, US-91920 fragm. ex P!).
Podosemum elegans Kunth, Nov. Gen. Sp. (quarto ed.) 1: 130. 1816. Trichochloaelegans (Kunth) Roem. & Schult., Syst. Veg. 2: 387. 1817. Agrostisquitensis Spreng., Syst. Veg. 1: 262. 1825. Type: Ecuador, Chimborazo, Paramo de las Puntas & Pomallacta, Jun, *F.W.H.A. Humboldt & A.J.A. Bonpland s.n.* (holotype: P!; isotype: BAA!).
Podosemum glabratum Kunth, Nov. Gen. Sp. (quarto ed.) 1: 130. 1816. Trichochloaglabrata (Kunth) Roem. & Schult., Syst. Veg. 2: 387. 1817. Agrostisglabrata (Kunth) Spreng., Syst. Veg. 1: 262. 1825. Type: Mexico, Santa Rosa de la Sierra and Cañada de Acabuca, Sep, *F.W.H.A. Humboldt & A.J.A. Bonpland s.n.* (holotype: P-Bonpl!; isotype: US-91921 fragm. ex P-Bonpl!).
Muhlenbergia berlandieri Trin., Mém. Acad. Imp. Sci. Saint-Pétersbourg, Sér. 6, Sci. Math., Seconde Pt. Sci. Nat. 6,4(3–4): 299. 1841. Type: Mexico, Distrito Federal, Mountains near México, Aug 1827, *J.L. Berlandier 676, 684* (lectotype, designated here: LE-TRIN-1487.01!, both collection numbers appear on the label with a single specimen and figure); México, 26 Aug 1827, *J.L. Berlandier 676* (isolectotypes: COL-000006382 [image!]; P-00644117 [image!], P-00644119 [image!], US-2557457!, US-87241 fragm!, W-239604!, W-0029177 [image!]).
Muhlenbergia affinis Trin., Mém. Acad. Imp. Sci. Saint-Pétersbourg, Sér. 6, Sci. Math., Seconde Pt. Sci. Nat. 6,4(3–4): 301. 1841. Podosemumaffine (Trin.) Bush, Amer. Midl. Naturalist 7(2):40. 1921. Type: Mexico, México, Toluca, *J.L. Berlandier 1083* (lectotype, designated here: P-00644141 [image!]; isolectotypes: G-00099411 [image!], G-00099410 [image!], G-00099409 [image!], LE-TRIN-1485.01 fragm.!, P-00644142 [image!], US-87237 fragm.!).
Muhlenbergia phragmitoides Griseb., Abh. Königl. Ges. Wiss. Göttingen 19: 255. 1874. Type: Argentina, Tucumán: Cuesta de Anfama, Sierra de Tucumán, 23 Mar 1872, *P.G. Lorentz 79* (lectotype, designated here: GOET-006649 [image!]; isolectotypes: BAA-00002225 [image!], CORD-00004622 [image!], GOET-006648 [image!], SI-002780 [image!], US-91911 fragm. ex GOET!).
Muhlenbergia elegans var. atroviolacea Kuntze, Revis. Gen. Pl. 3(3): 357. 1898. Type: Bolivia, Cochabamba, 3000 m, 26 Mar 1892, *O. Kuntze s.n.* (lectotype, designated here: NY-00381485 [image!].
Muhlenbergia elegans var. subviridis Kuntze, Revis. Gen. Pl. 3(3): 357. 1898. Type: Bolivia, Tunari Mts, 1600 m, *O. Kuntze* (lectotype, designated here: NY-00381486 [image!]).
Muhlenbergia metcalfei M.E. Jones, Contr. W. Bot. 14: 12. 1912. Type: U.S.A. New Mexico: Grant Co., Santa Rita Mountains, in and around S end of the Black Range, 7000 ft alt., 9 Oct 1904, *O.B. Metcalf 1485* (holotype: POM-116640!; isotypes: GH-00023980 [image!], MO!, US!).
Muhlenbergia holwayorum Hitchc., Contr. U.S. Natl. Herb. 24(8): 389. 1927. Type: Bolivia, Sorata, 16 Apr 1920, *E.W.D. Holway & M.M. Holway 530* (holotype: US-1108445!).

##### Description.

Densely caespitose *perennials*. *Culms* 40–100 cm tall, stiffly erect, glabrous to scaberulous below the basal, terete nodes, usually 1 node per culm; *internodes* mostly glabrous. *Leaf sheaths* 2–30 cm long, longer than the internodes, glabrous to scaberulous, rounded near base; *ligules* (1–)3–6(−8) mm long, often lacerate, firmer below, strongly decurrent, apex obtuse to acute; *blades* 12–35 cm long, 1–3 mm wide, flat or involute, glabrous to scaberulous below and scaberulous to hirsutulous above. *Panicles* (4–)10–35 cm long, (2–)3–5(–15) cm wide, loosely contracted to open and lax, reddish-purple; *primary branches* 0.4–10 cm long, sometimes capillary, ascending and spreading up to 80° from the rachises; *pedicels* 1–10 mm long, mostly longer than the spikelets. *Spikelets* 3.5–5 mm long, reddish-purple; *glumes* 1–1.7(–2) mm long, much shorter than the floret, about equal, 1-nerved, unawned, apex obtuse to subacute, sometimes hirsutulous, rarely mucronate; *lemmas* 3.5–5 mm long, narrow lanceolate, scaberulous to scabrous, purple, awned, callus with hairs up to 0.5 mm long, apex acuminate, the awns (8–)10–22 mm long, flexuous; *paleas* 3.5–5 mm long, narrow lanceolate, purple, scaberulous, apex acuminate; *anthers* 1.7–2.3 mm long, reddish-purple. *Caryopses* 2–3.5 mm long, fusiform, brownish. 2*n* = 40, 44.

##### Distribution.

*Muhlenbergiarigida* ranges from Arizona, New Mexico and south-western Texas, throughout México and Central America to South America where it occurs along the Andes from Columbia, Venezuela, Ecuador, Bolivia, Peru and Argentina.

##### Ecology.

This species occurs on rocky slopes, ravines and sandy, gravelly slopes derived from granitic and calcareous substrates associated with *Acacia*, *Agave*, *Aristida*, *A.adscensionis*, *Baccharis*, *Berberis*, *Bidens*, *Boutelouacurtipendula* (Michx.) Torr., *Caesalpinia*, *Calamagrostis*, *Colletiaspinosissima*, *Cortaderiabifida*, *C.jubata*, *Desmodium*, *Dodonaeaviscosa*, *Ephedra*, *Eragrostis*, *Eupatorium*, *Festuca*, *Fucaria*, *Hypericum*, *Jarava*, *Krameria*, *Lepechinia*, *Lupinus*, *Lycium*, *Melinusminutiflora*, *Mirabilis*, *Opuntia*, *Paspalum*, *Cenchrusclandestinus*, *Peperomia*, *Puya*, *Salvia*, *Schinusmolle*, *Schizachyrium*, *Sporobolus*, *Tillandsia* and *Trichocereus*; 2000–3650 m.

##### Comments.

This species is highly variable and is one of the most common upland bunchgrasses forming almost pure stands in northern México, less common in Peru and South America where it is usually found in smaller populations. *Muhlenbergiarigida* can be distinguished morphologically from *M.coerulea* and M.coerulea×rigida in having shorter lemmas 3.5−5 mm long with long flexuous awns (8–)10–22 mm long and shorter glumes 1–1.7(–2) mm long.

Molecular DNA sequence analysis indicates *M.rigida* lies within Muhlenbergiasubg.Trichochloa and genetically is highly variable (Fig. 1A; Peterson et al. 2010b).

##### Specimens examined.

Peru. **Amazonas**: 66 km from Leymebamba towards Balsas, S.A. Renvoize & S. Lægaard 4934 (CPUN); Prov. Chachapoyas, Cerro Puma Urco S of Chachapoyas, C.P. Cowen 4308, J. Canne & V. Torrel (USM). **Ancash**: E of Yungay, S.A. Renvoize & S. Lægaard 5053 (CPUN); 10 km from Huaraz towards Casma, S.A. Renvoize & S. Lægaard 5153 (CPUN); Prov. Bolognesi, Tanás, E. Cerrate 1381 (US, USM); Aynín, E. Cerrate 1341 (USM); Casca, below Chiquián, R. Ferreyra 7285 (US, USM); above Chiquián, E. Cerrate 372, 480 (US, USM); Shapash, near Chiquián, F. Ferreyra 5696 (US); Yungay, Prov. Huaraz, 23 Apr 1995, S. Llatas Quiroz 3630 & J. Campos de la Cruz (USM); Prov. Huaraz, W side of Cordillera Blanca, S of quebrada Ishinca, 9°24'55.8"S, 77°32'45.8"W, 2885 m, 12 Mar 2008, P.M. Peterson 21610, R.J. Soreng, M. La Torre & J.V. Rojas Fox (US, USM); Corongo, N end of Cordillera Blanca, 12 km W of Tarica towards Yuacmarca, 8°35'55.6"S, 77°50'4.8"W, 2849 m, 18 Mar 2008, P.M. Peterson 21782 & R.J. Soreng (US, USM); Cordillera Negra E slope, S side of quebrada W of Shupluy, 9°13'30.0"S, 77°42'6.6"W, 2476 m, 13 Mar 2008, P.M. Peterson 21628, R.J. Soreng, M. La Torre & J.V. Rojas Fox (US, USM); Prov. Huaraz-Recuay border, W side of Rio Santa Canyon, 9°40'58.1"S, 77°28'29.7"W, 3202 m, 11 Mar 2008, P.M. Peterson 21605, R.J. Soreng, M. La Torre & J.V. Rojas Fox (US, USM); Prov. Huari, Cordillera Blanca, 21 km S of Huari on road towards San Marcos, 2850 m, 22 Mar 1997, P.M. Peterson & N. Refulio Rodriguez 13862 (US, USM); Uco near Rio Puchca, A. Cano 14317, M.I. La Torre, W. Mendoza & C. Mendoza (USM); San Marcos, A. Cano 13395, W. Mendoza, I. Salinas & A. Ramirez (USM); Prov. Huaylas, below Huaylas, E. Carrillo 1297, W. Medina & P. Huamán (USM); Pueblo Libre, A. Cano 11616 (USM); Prov. Pallasca, 3.4 km N of Huandoval towards Huascaschuque and Pallasca, 8°18'59.2"S, 77°58'9.5"W, 2949 m, 21 Mar 2008, P.M. Peterson & R.J. Soreng 21818 (US, USM); divide between Rio Conchucos and Rio Plata, 8°12'9.1"S, 77°56'20.6"W, 2233 m, 21 Mar 2008, P.M. Peterson & R.J. Soreng 21834 (US, USM); Prov. Recuay, Catac, L.I. Masias 47 (USM); Prov. Yungay, Cordillera Negra, 18 km SW of Shuplay and Rio Santa, 9°14'52.7"S, 77°43'44.1"W, 2901 m, 13 Mar 2008, P.M. Peterson 21637, R.J. Soreng, M. La Torre & J.V. Rojas Fox (US, USM); Cordillera Blanca W slope, 5 km E of Yungay, below Shillcop, 9°9'11.7"S, 77°43'27.6"W, 2597 m, 15 Mar 2008, P.M. Peterson 21687, R.J. Soreng, M. La Torre & J.V. Rojas Fox (US, USM); Kuisho, M. La Torre 419 (USM); Shupluy, A. Cano 11354 (USM); Shilco, E. Cerrate 7753 (USM). **Apurimac**: Prov. Grau, Cotobambas−Cochapata, C. Vargas C. 5789 (US). **Ayacucho**: A. Weberbauer 5525 (US); Prov. Lucanas, Quebrada Chuela, 3 Apr 1942, R.D. Metcalf 30315 (LIL); Prov. Huamanga, Quellagocha, 4000 m, 5 Apr 1967, V. Palomino 242 (USM); Prov. Huamanga, 4 km S of Ayacucho on hwy 3 towards Abancay, 13°11'33.8"S, 74°12'2.1"W, 2952 m, 15 Mar 2007, P.M. Peterson 20475, R.J. Soreng, K. Romaschenko & D. Susanibar Cruz (US, USM); 21 km S of Ayacucho on hwy 3 towards Abancay, 13°16'21.5"S, 74°13'46.8"W, 3575 m, 15 Mar 2007, P.M. Peterson 20487, R.J. Soreng, K. Romaschenko & D. Susanibar Cruz (US, USM); Cerro Acuchimay above Ayacucho, O. Tovar 5399 (USM); Huatalás, V. Palomino 34 (USM); Prov. Huanca Sancos, 27 km NW of Putajasa and 3 km S of Sacsamarca, 13°57'51.1"S, 74°18'41.5"W, 3650 m, 25 Feb 2002, P.M. Peterson 16273A, A. Cano, M. La Torre, A Ramirez & D. Susanibar (US, USM); Prov. Huata, Rio Cachi below Huanta, O. Tovar 4946 (US, USM); Prov. Lucanas, near Puquio, R. Ferreyra 7187 (MOL,US, USM); vicinity of Puquio, O. Tovar 9 (USM). **Cajamarca**: near side road to Chugur, 95 km from Cajamarca, S.A. Renvoize 4847, S. Lægaard & I. Sánchez Vega (CPUN); above Celendín, S.A. Renvoize & S. Lægaard 4877 (CPUN); Prov. Cajabamba, 14 km WSW of Cajabamba, E of Araqueda, 7°39'9.1"S, 78°9'58.9"W, 2242 m, 23 Mar 2008, P.M. Peterson & R.J. Soreng 21854 (US, USM); Prov. Cajamarca, 6 km NW of San Marcos on road towards Cajamarca, 2570 m, 30 Mar 1997, P.M. Peterson & N. Refulio Rodriguez 14009 (US, USM); 1 km S of Huambocancha on road towards Cajamarca, 2770 m, 14 Mar 2000, P.M. Peterson & N. Refulio Rodriguez 14853 (CPUN, US, USM); Parque forestall Aylambo, 4 km de Cajamarca, I. Sánchez Vega 987 (CPUN); 4 km S of Jesús, I. Sánchez Vega 2376 (CPUN); Tres Molinos, T. Tejada 46 (CPUN); S of Paso El Gavelán, 10 air km S of Cajamarca, 7°15'37.2"S, 78°30'34.5"W, 2544 m, 26 Mar 2008, P.M. Peterson 21878, R.J. Soreng & I. Sánchez Vega (US, USM); near Gavilán and San Juan, I. Sánchez Vega & W. Ruiz Vigo 650 (CPUN); bajando Paso El Gavilán a San Juan, I. Sánchez Vega & W. Ruiz Vigo (CPUN); near Paso El Gavilán, I. Sánchez Vega 1400, P. Brandelard & J. Sanabria (CPUN); bajadas de Chancay a Valle de Condebamba, I. Sánchez Vega 1112 (CPUN); above San Pablo, I. Sánchez Vega 1495 (CPUN); near Jesús, I. Sánchez Vega 1066 (CPUN); S of Jesús Tabada, I. Sánchez Vega 2376 (CPUN, US); between Cajamarca and Celendín, R. Ferreyra 15004 (USM); 20 km from Celendín, P. Gutti & G. Müller s.n. (USM); Chotén, 4 km de la carretera Pacasmayo, I. Sánchez Vega 2569, V. Torrel & E. Medina (CPUN); Bellavista, I. Sánchez Vega 1842, W. Ruiz Vigo & J. Sánchez Vega (CPUN); Chotén Bajo, J. Cabanillas S. & J. Guevara B. 665 (CPUN); Cerro Rumicucho, km 11 de la carretera Cajamarca Cajabamba, I. Sánchez Vega 2492, V. Torrel & E. Medina (CPUN); near Chancay and Valle de Condebamba, I. Sánchez Vega 2412, V. Torrel & E. Medina (CPUN); Cerro Huacarís, Valle de Cajamarca, I. Sánchez Vega 444 (CPUN); Prov. Celendín, above Limon, 27 km E of Celendín, 6°53'15.2"S, 78°5'30.5"W, 2215 m, 29 Mar 2008, P.M. Peterson 21927, R.J. Soreng & J. Montoya Quino (US, USM); Marañón river Valley, Chachapoyas−Cajamarca road, D.N. Smith & J. Cabanillas 7272 (MO, US); Prov. Chota, 5 km de Cochabamba a Cutervo, I. Sánchez Vega 4845 (CPUN); 6 km de Cochabamba, I. Sánchez Vega 2334 (CPUN); Prov. Contumaza, Hacienda San Lorenzo, San Benito, A. Sagástegui 3784 (US), Cerro Campanillas, A. Sagástegui 2992 (US); Shamon, A. Sagástegui 14305 (HAO); Prov. Cutervo, 13 km W of Cutervo on road towards Socoto, 2160 m, 20 Mar 2000, P.M. Peterson & N. Refulio Rodriguez 15008 (CPUN, US, USM). **Cusco**: 2 km S of San Jeronimo and 10 km SE of Cusco, J.C. Soloman 2985 (USM); Prov. Anta, Mollepata, C. Vargas C. 19049 (USM); Prov. Calea, Pisai, F. Marin 512a (LIL); Prov. Cusco, Cusco−Huancar, P. Gutti & G. Müller 94269b (USM); Prov. Urubamba, Cerro Chicón, P. Nuñez V. 8895 (MO, US); Chincheros, just below Perga Kachun, S. King 316, E. Franquemont, C. Franquemont & C. Sperling (USM); lower end of quebrada Pumahuanca, H.H. Iltis 855, C.M. Iltis & C. Vargas C. (LIL, USM); Machu Pichu, I. Grignon 2305 (US); ESE of Cusco, H. Ellenberg 1018 (US); Prov. Yaurisque, Paruro, SW of Cusco, P. Nuñez 7383 (USM). **Huancavelica**: Prov. Huancavelica, Mariscal Cáceres, O. Tovar 1405 (US, USM); Prov. Tayacaja, near Huachocolpa, O. Tovar (US, USM); near Surcubamba, O. Tovar 4240 (US, USM); Mejorada, O. Tovar 2454 (US, USM); Mamacha-rumi, O. Tovar 2439 (US, USM); Prov. Taycaya, Valle del Rio Mantero, A. Weberbauer 6448 (MOL). **Huánuco**: Prov. Ambo, above Hacienda Quicacán, near Huánuco and Ambo, R. Ferreyra 9228 (US, USM); Huarmiragra, A. Granda Paucar 1791 (USM); Prov. Huánuco, 7 km S of Ambo on hwy 3 towards Cerro de Pasco, 10°11'49"S, 76°9'54"W, 2345 m, 6 Mar 2007, P.M. Peterson 20322, R.J. Soreng & K. Romaschenko (US, USM); Cerro San Cristobal, P. Aguilar 808 (USM); Prov. Pachitea, 21 air km NE of Huánuco, 7 km E of Puerto Rancho junction, 9°49'45.9"S, 76°3'12.9"W, 2094 m, 6 Mar 2007, P.M. Peterson 20334, R.J. Soreng & K. Romaschenko (US, USM). **Junín**: Chorrillos, B. Maass 487 (USM); Prov. Huancayo, near Huancayo, O. Tovar 2747 (US, USM); Prov. Huancayo, Acopalca, 20 Jan 1952, J. Infantes Vera 1586 (LIL); Huancayo, 3317 m, Mar 1943, J. Soukup 1899 (COL, US); Montaro Valley W of Huancayo, 3800 m, 14 Jul 1982, S.A. Renvoize 4328 (USM); below Pariahuana, O. Tovar 7899, 7944 (USM); Prov. Tarma, 1 km up road to Hacienda Maraynioc out of Palca, 2800 m, 6 Apr 1997, P.M. Peterson & O. Tovar 14055 (US, USM); Tarma, E.P. Killip & A.C. Smith 21802 (US); A.S. Hitchcock 22165 (US). **La Libertad**: Usquil, E. Anderson 1293 (US); Prov. Bolivar, 0.5 km W of Longotea towards San Vicente, 7°2'35.6"S, 77°52'39.8"W, 2487 m, 31 Mar 2008, P.M. Peterson 21968, R.J. Soreng & J. Montoya Quino (US, USM); 4.6 km NW of Longotea, SE of San Vicente, 7°00'38.0"S, 77°54'15.2"W, 2058 m, 31 Mar 2008, P.M. Peterson 21969, R.J. Soreng & J. Montoya Quino (US, USM); Prov. Huamachuco, Llautobamba, J. Infautes 5509 (US); Prov. Santiago de Chuco, 22 km E of Huamachuco on road towards Sarin, 2500 m, 29 Mar 1997, P.M. Peterson & N. Refulio Rodriguez 13984 (US, USM). **Lima**: Prov. Huarochiri, Tambo de Viso, J.F. Macbride & Featherstone 765 (F, US); Matucana, J.F. Macbride & Featherstone 358 (F, US). **Pasco**: 10 km unterhalb Tarma, Ellenberg 8787 (USM). **Piura**: Prov. Huancabamba, 7 km E of Sondor on road towards Tabaconas, 2200 m, 31 Mar 2000, P.M. Peterson & N. Refulio Rodriguez 15162 (CPUN, US, USM); Abra de Porculla, R. Ferreyra 13734 (US, USM); 11 km Huancabamba−Salalá, I. Sánchez Vega 5160 (CPUN).

#### 18. Muhlenbergia romaschenkoi

Taxon classificationPlantaePoalesPoaceae

P.M. Peterson sp. nov.

urn:lsid:ipni.org:names:77192895-1

Fig. 12A−L

##### Type.

Peru. Depto. Huánuco, Pachitea, 21 air km NE of Huánuco, 7 km E of Puerto Rancho jtn on road to Panao, along Río Huallaga, 9°49'45.9"S, 76°3'12.9"W, 2094 m, 6 Mar 2007, *P.M. Peterson 20331, R.J. Soreng & K. Romaschenko* (holotype: US-3730646!; isotypes: MO!, USM!).

##### Description.

Loosely caespitose to densely tufted, *annuals* to short-lived *perennials* with delicate bases, flowering the first year. *Culms* 20–40 cm tall, erect or decumbent at the base, branching at the lower and middle nodes, scaberulous below the terete nodes; *internodes* generally 2.0–9.5 cm long. *Leaf sheaths* 4.0–7.5 cm long, glabrous or scaberulous, commonly shorter than the internodes; *ligules* 1.2–3.0(–5.0) mm long, hyaline, apex acute to obtuse, often lacerate with age; *blades* 3–12 cm long, 1.2–2.5 mm wide, flat or loosely involute, scaberulous to glabrous below and scabrous above. *Panicles* 7–15 cm long, 0.6−2 cm wide, narrow and contracted to loosely spreading, interrupted below, terminal and axillary, 15–23 nodes per panicle; *primary branches* 3.5–7.5 cm long, usually one per node, when immature the branches mostly appressed and ascending, when mature the branches sometimes widely spreading up to 50° from the rachises; *pedicels* 1–3 mm long, usually shorter than the spikelets, antrorsely scabrous, stout, appressed. *Spikelets* 2–4 mm long, erect, stramineous to purplish; *glumes* 1–2.8 mm long, unequal, 1-nerved, scabrous along the nerves, apex acute to acuminate, often mucronate or erose, the mucro up to 0.5 mm long; *lower glumes* 1–2.0 mm long; *upper glumes* 1.5–2.8 mm long, more than ½ as long as the lemma; *lemmas* 2.0–3.5(–4.0) mm long, lanceolate, widest near base, awned, scaberulous above and villous on proximal 1/2 along the margins and the midvein, the hairs 0.5–1.5 mm long, callus short-pubescent, the awn 10–30 mm long, scabrous, flexuous; *paleas* 1.8–3.4(–3.8) mm long, lanceolate, sparsely appressed pubescent between the nerves on the proximal ½; *anthers* 0.3−0.4 mm long, purplish, yellow with age. *Caryopses* 1.0–2.2 mm long, narrowly fusiform, terete, brownish.

**Figure 12.**
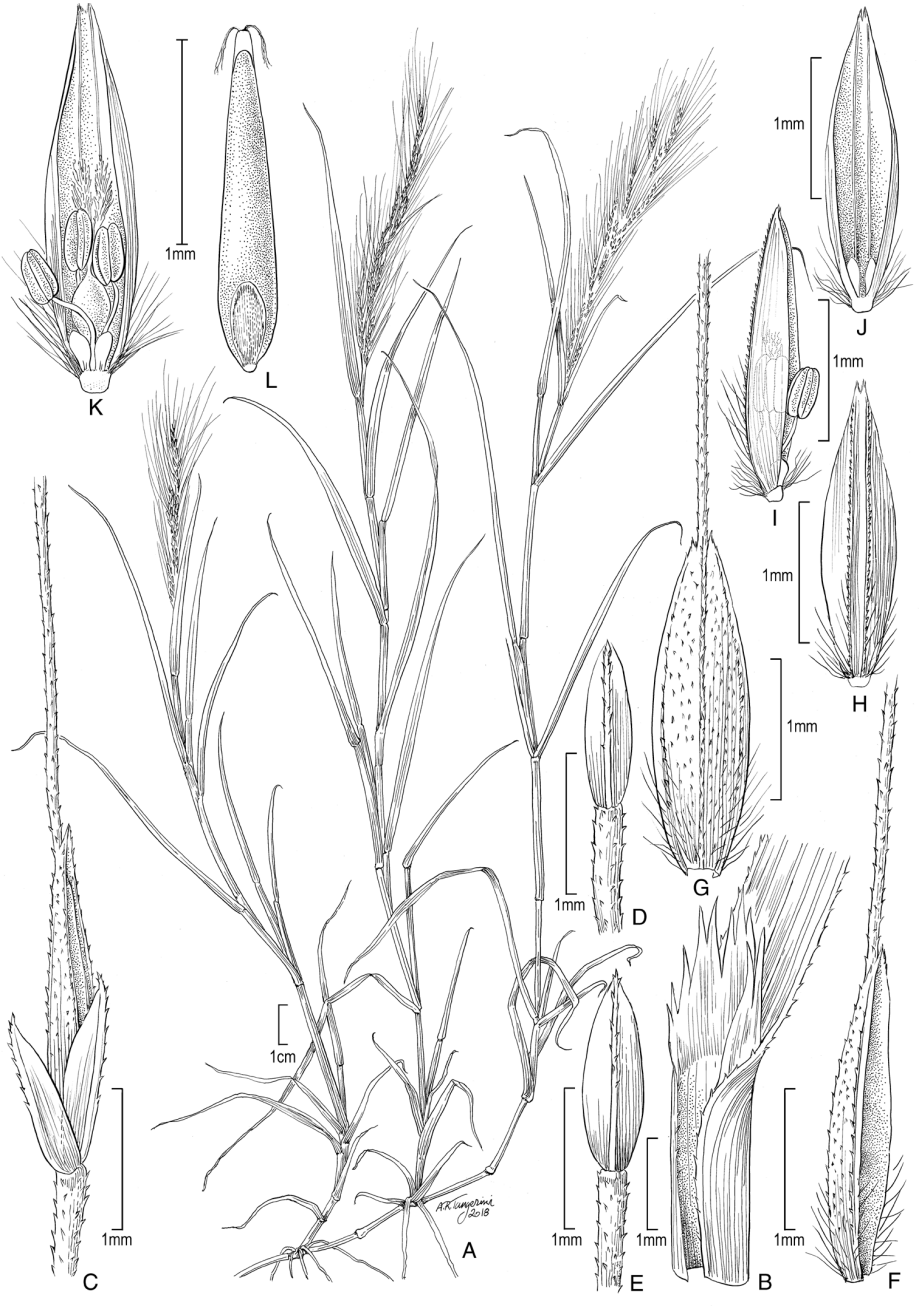
*Muhlenbergiaromaschenkoi* P.M. Peterson **A** habit **B** ligule **C** spikelet **D** lower glume **E** upper glume **F** lemma, lateral view **G** lemma, dorsal view **H** palea, dorsal view **I** palea, lateral view **J** palea, ventral view **K** lodicules, stamens, and pistil **L** caryopsis. Drawings from the holotype collection *P.M. Peterson, R.J. Soreng & K. Romaschenko 20331*.

##### Distribution.

Endemic to Peru and known only from Huánuco.

##### Ecology.

*Muhlenbergiaromaschenkoi* grows on rocky slopes and limestone rock outcrops in grasslands with *Aristidaadscensionis*, *Bidens*, *Bothriochloa*, *Eragrostisnigricans* (Kunth) Steud., *Eupatorium*, *Melinusminutiflora*, *Microchloakunthii* Desv., *Muhlenbergiaciliata*, *M.rigida and Nassella*; 1800–2500 m.

##### Etymology.

We honour Dr. Konstantin Romaschenko (born 1969) who accompanied PMP and Robert J. Soreng on the collecting trip in which material of the species was gathered.

##### Comments.

*Muhlenbergiaromaschenkoi* can be separated from *M.microsperma* in having longer acute to acuminate glumes (1–2.8 mm long) and by lacking cleistogamous panicles present in the axils of the lower sheaths. *Muhlenbergiatenuifolia* (Kunth) Kunth, a species ranging from southern Arizona, New Mexico and Texas, U.S.A., throughout México and then again in Venezuela, Bolivia and Argentina, is morphologically similar to *M.romaschenkoi* but differs in having yellowish anthers 0.9−1.5 mm long (Peterson and Annable 1991; Peterson 2003; Peterson et al. 2007b; Peterson and Giraldo-Cañas 2011, 2012). Initially we thought the new species was *M.tenuiofolia* with immature anthers 0.3−0.4 mm long. However, our collection of *M.romaschenkoi* includes well-developed florets with mature caryopses and anthers. Therefore, the observed small anthers in *M.romaschenkoi* are not a result of immaturity but represent a genetic difference, from *M.tenuifolia*, which is not known in Peru.

*Muhlenbergiaromaschenkoi*, a member of M.subg.Muhlenbergia, is sister to *M.spiciformis* Trin. (plastid marker), a species known from south-western United States and throughout Mexico whereas ITS marker aligns it within the *M.tenuifolia* clade (Fig. 1B). *Muhlenbergiaspiciformis* differs from the new species in having shorter glumes 0.3−1 mm long and larger anthers 0.9−1.6 mm long (Peterson 2003; Peterson et al. 2007b). These results suggest multiple origins for *M.romaschenkoi* from North and South American progenitors. Similar to *M.caxamarcenis*, this scenario appears to be a common pattern of speciation for South American species of *Muhlenbergia*, needing further study.

##### Specimen examined.

Peru. **Huánuco**: between Huánuco and Tingo María, vereda Taruka, 17 km NE of Huánuco in valley of Río Huallaga, T. Croat 57781 (MO).

#### Infrageneric classification of the species of *Muhlenbergia* in Peru

Muhlenbergiasubg.Bealia (Scribn.) P.M. Peterson: *M.caxamarcensis, M.ligularis*.

Muhlenbergiasubg.Clomena (P. Beauv.) Hack.: *M.peruviana*.

Muhlenbergiasubg.Muhlenbergia: *M.beyrichiana, M.bryophilus, M.cenchroides, M.ciliata, M.diversiglumis, M.flexuosa, M.microsperma, M.romaschenkoi*.

Muhlenbergiasubg.Pseudosporobolus (Parodi) P.M. Peterson: *M.fastigiata, M.monandra, M.phalaroides*.

Muhlenbergiasubg.Trichochloa (P. Beauv.) A. Gray: *M.coerulea, M.coerulea × M.rigida, M.maxima, M.rigida*.

#### Species excluded

##### Muhlenbergia tenuissima

Taxon classificationPlantaePoalesPoaceae

(Presl) Kunth, Enum. Pl. 1: 198. 1833.

###### Discussion.

This taxon is a distinctive species which seems to be restricted to chalky limestone flats usually occurring as a dense carpet on clay-like soils in open savannahs; known from North America and Central America (Peterson and Annable 1991; Brako and Zarucchi 1993). Although previously included for Peru in Hitchcock (1927), Standley (1936) and Tovar (1993), the specimens cited are *M.ciliata* without prominent cilia along the lateral veins (see discussion of *M.ciliata*).

##### Muhlenbergia tenuifolia

Taxon classificationPlantaePoalesPoaceae

(Kunth) Kunth, Révis. Gramin. 1(4):63. 1829.

###### Discussion.

This taxon was reported for Peru in Brako and Zarucchi (1993) who cited *Croat 57781* which we place in *M.romaschenkoi*.

## Supplementary Material

XML Treatment for Muhlenbergia

XML Treatment for Muhlenbergia beyrichiana

XML Treatment for Muhlenbergia bryophilus

XML Treatment for Muhlenbergia caxamarcensis

XML Treatment for Muhlenbergia cenchroides

XML Treatment for Muhlenbergia ciliata

XML Treatment for Muhlenbergia coerulea

XML Treatment for Muhlenbergia coerulea

XML Treatment for Muhlenbergia diversiglumis

XML Treatment for Muhlenbergia fastigiata

XML Treatment for Muhlenbergia flexuosa

XML Treatment for Muhlenbergia ligularis

XML Treatment for Muhlenbergia maxima

XML Treatment for Muhlenbergia microsperma

XML Treatment for Muhlenbergia monandra

XML Treatment for Muhlenbergia peruviana

XML Treatment for Muhlenbergia phalaroides

XML Treatment for Muhlenbergia rigida

XML Treatment for Muhlenbergia romaschenkoi

XML Treatment for Muhlenbergia tenuissima

XML Treatment for Muhlenbergia tenuifolia
